# Comparative effectiveness of aerobic training intensities in chronic stroke: a network meta-analysis of randomized controlled trials

**DOI:** 10.3389/fneur.2026.1808908

**Published:** 2026-07-06

**Authors:** Qiu Peng, Yidong Xia, Haoyuan Hei

**Affiliations:** 1 School of Physical Education, Jilin University, Changchun, China; 2 School of Philosophy and Sociology, Jilin University, Changchun, China

**Keywords:** aerobic exercise, cardiorespiratory fitness, chronic stroke, high-intensity interval training, mobility, moderate-intensity continuous training, network meta-analysis

## Abstract

**Objective:**

This study aims to conduct a systematic review and network meta-analysis of randomized controlled trials (RCTs) to assess the effects of different aerobic exercise modalities on cardiopulmonary fitness and functional recovery outcomes [including VO_2_peak, 6-Minute Walk Test (6MWT), 10-Meter Walk Test (10MWT), Berg Balance Scale (BBS), and Time Up and Go (TUG)] in chronic stroke patients (≥3 months post-stroke).

**Methods:**

This study employed a network meta-analysis (NMA) based on 33 RCTs involving 1,665 participants. The analysis followed the Preferred Reporting Items for Systematic Reviews and Meta-Analyses extension for Network Meta-Analyses (PRISMA-NMA) guidelines. The primary outcomes assessed included VO_2_peak, 6MWT, 10MWT, BBS, and TUG. A consistency-based random-effects model was used to evaluate the effects of different exercise intensities on these outcomes. To assess the robustness of the findings, sensitivity analysis was performed by sequentially excluding each study and re-estimating the effect size of each intervention relative to Standard Of Care (SOC). This ensured that the overall results were not influenced by any single study. Additionally, linear regression analysis was conducted to evaluate the potential modifying effects of baseline gait speed, total intervention duration, baseline VO_2_peak and age on the therapeutic effects of the interventions.

**Results:**

A total of 33 studies were included in the analysis, with 20 studies (n = 1,040) assessing VO_2_peak, 20 studies (*n* = 1,113) on the 6MWT, 13 studies (*n* = 566) on the 10MWT, and 12 studies (*n* = 504) on the BBS and 7 studies (*n* = 297) on the TUG. Both High-Intensity Interval Training (HIIT) and Moderate-Intensity Continuous Training (MICT) demonstrated the most significant effects on VO_2_peak, 6MWT, and 10MWT. For BBS or TUG, there are no statistically significant differences between the vast majority of interventions. Surface under the cumulative ranking curve (SUCRA) rankings tended to favor HIIT and MICT for VO_2_peak and walking outcomes, but confidence in comparative effects was limited. Evidence certainty assessed with CINeMA was predominantly low to very low across comparisons, particularly for BBS and TUG.

**Conclusion:**

HIIT and MICT may improve cardiorespiratory fitness and walking performance after chronic stroke; however, the certainty of evidence is largely low or very low, and results should be interpreted cautiously. Effects on balance and TUG remain inconclusive.

**Systematic review registration:**

https://www.crd.york.ac.uk/PROSPERO/view/CRD420251171092, Identifier: CRD420251171092.

## 1 Introduction

Stroke is a critical public health concern, ranking as the second leading cause of death worldwide and the third primary contributor to disability (1). With the incidence of stroke increasing from 1.1 million/year in 2000 to 1.5 million/year in 2025 (2). Patients following a stroke often face motor impairments, muscle weakness, compromised coordination, fatigue, and cognitive dysfunction. These factors significantly hinder their ability to engage in physical activity. Over time, this can lead to cardiovascular decline and muscle atrophy, further exacerbating the overall deterioration of functional capacity. Such a vicious cycle ultimately contributes to a continuous decline in physical condition (3, 4). On the other hand, the recovery process following a stroke is often slow, with more than 50% of chronic stroke patients still experiencing hemiparesis, significantly impairing their quality of life (5).

Regular physical exercise is a key intervention for alleviating the decline in physical function following a stroke and for improving daily living activities (6). Aerobic exercise has been shown to increase mitochondrial density and oxidative metabolism efficiency, enhance V̇O_2_peak, and improve muscle endurance, thereby promoting cardiovascular adaptation (7), which widely regarded as an ideal choice for rehabilitation following a stroke (8). Exercise intensity is considered a key factor in the implementation of aerobic intervention programs. The impact of different intensities varies in terms of maintaining and enhancing the aerobic capacity of peripheral muscles and cardiovascular performance (9, 10). Moderate-Intensity Continuous Training (MICT) can enhance neuroplasticity in stroke patients (11). As highlighted in several previous reviews, MICT significantly improves V̇O_2_peak, mobility (6-Minute Walk Test distance, 10-Meter Walk Test, and Time Up and Go) (12) or balance ability (Berg Balance Scale) (13) compared to Standard Of Care (SOC) or Non-training in stroke patients. As exercise intensity gradually increases, the training benefits also correspondingly improve (14). An increasing body of research suggests that High-Intensity Interval Training (HIIT) may significantly enhance post-stroke functional recovery and cardiovascular function through the activation of various neural mechanisms (15–17). Therefore, Kevin M et al. (18) systematically analyzed aerobic exercise intensity after stroke and suggested that higher intensity may yield greater improvements. However, most previous studies included participants across all post-stroke stages (acute, subacute, and chronic) rather than focusing specifically on chronic stroke. Because substantial spontaneous recovery typically occurs within the first 3 months after stroke (19) and varies considerably across individuals (3), isolating the treatment effect of exercise during earlier phases can be challenging. In contrast, neurological recovery tends to plateau after approximately 3 months post-stroke (20). Therefore, evaluating exercise therapy in the chronic phase is particularly important, as it reduces confounding from spontaneous recovery.

Existing reviews on chronic stroke primarily focus on discussing the effectiveness of aerobic exercise (13, 21) or compare the impact of different exercise modalities on walking ability in chronic patients (22, 23). Thus, systematic comparisons of the effects of different aerobic exercise intensities on chronic stroke patients remain a gap in the literature. Based on this, the aim of the present study is to conduct a systematic review and network meta-analysis of Randomized Controlled Trials (RCTs) to explore the effects of different exercise modalities on outcomes such as V̇O_2_peak, 6-Minute Walk Test distance (6MWT), 10-Meter Walk Test (10MWT), Berg Balance Scale (BBS), and Time Up and Go (TUG) in chronic stroke patients.

## 2 Materials and methods

This network meta-analysis (NMA) was conducted in accordance with the Preferred Reporting Items for Systematic Reviews and Meta-Analyses extension for Network Meta-Analyses [PRISMA-NMA; see Supplementary Table 1, (24)]. Given the limited number of RCTs that directly compare the therapeutic effects of aerobic exercise prescriptions of varying intensities in patients with chronic stroke, NMA provides a valuable methodological framework for enabling indirect comparisons and probabilistic ranking of different aerobic interventions according to their effects on cardiopulmonary fitness and functional outcomes (25). To ensure transparency, methodological rigor, and novelty, the study protocol was prospectively registered in the International Prospective Register of Systematic Reviews (PROSPERO; CRD420251171092).

### 2.1 Data sources and search strategy

A systematic search was performed across six major electronic databases: PubMed, EMBASE, the Cochrane Library, Scopus, Web Of Science, and EBSCOhost. The search strategy combined free-text terms and controlled vocabulary (MeSH/Emtree) using the following keywords: “Cerebrovascular Accident,” “Stroke,” “Vascular Accident, Brain,” “Cerebrovascular Apoplexy,” “Cerebrovascular Accident, Acute,” “Aerobic Exercise,” “Physical Activity,” “Exercise Training,” “Acute Exercise,” and “Randomized Controlled Trial” (see Supplementary Table 2 for the full search strategy). The search covered all available records from the inception of each database through September 18, 2025. No language restrictions were applied.

### 2.2 Selection criteria

#### 2.2.1 Inclusion criteria

(1) The included intervention population consists of stroke patients (ischemic or hemorrhagic) who have been diagnosed through imaging or clinical assessment, are aged ≥18 years, and are in the chronic phase (with a duration of ≥3 months from onset to the start of intervention). Articles that explicitly identify the participants as chronic stroke patients but do not specify the exact duration of the condition are also considered to meet the inclusion criteria for this study.
(2) RCTs included in this study must have at least one intervention group that uses aerobic exercise as the intervention modality, and related studies (8, 26) suggested that aerobic exercise intensity be categorized based on the percentage of the patient-reported maximum heart rate, heart rate reserve, or subjective rating of perceived exertion: low (<54% HRmax, <40% HRR, <11/20 RPE), moderate (55–69% HRmax, 40–59% HRR, 12–15/20 RPE) or high (>70% HRmax, >60% HRR, >15/20RPE). Therefore, this study categorizes the aerobic exercise interventions in each group as follows: High-Intensity Interval Training (HIIT), High-Intensity Continuous Training (HICT), Moderate-Intensity Continuous Training (MICT), and Low-Intensity Continuous Training (LICT). HIIT refers to exercise that is characterized by relatively short bursts of vigorous activity, interspersed by periods of rest or low-intensity exercise for recovery (27). Additionally, if there is overlap in exercise intensity across certain exercise programs, the researchers, for the sake of conservatism, categorized them according to the reported minimum intensity.
(3) The control group can be classified into any of the four categories mentioned above or as Standard Of Care (SOC; eg, usual stroke rehabilitation typically includes gait training, active range of motion, stretching, light resistance training, balance or functional training) or Non-training.
(4) Included studies must report at least one of the following indicators: 6MWT, 10MWT, V̇O_2_peak, BBS, or TUG. The 6MWT is a widely used assessment tool for measuring walking endurance and functional walking ability in stroke rehabilitation. It quantifies the total distance a patient can walk in 6 min, reflecting both cardiovascular and neuromotor function (28). 10MWT test measures gait speed over a short distance, providing insight into a patient’s ability to walk efficiently and independently (29). The VO_2_peak is a critical measure of cardiorespiratory fitness. It reflects the maximal amount of oxygen the body can utilize during intense exercise. This measure was used to assess the efficacy of aerobic exercise interventions in improving cardiovascular health and physical function in chronic stroke patients (30). And, the BBS is a clinical tool used to assess balance and fall risk in stroke patients. Improvements in BBS scores can indicate significant recovery in postural control (31), which is critical for reducing fall risk post-stroke. Lastly, the TUG test is sensitive to improvements in mobility and reflects the combined influence of walking speed, balance, and strength (32).
(5) The studies must be RCTs.

#### 2.2.2 Exclusion criteria

(1) Participants with severe psychiatric disorders, acute complications, or serious health issues (e.g., severe heart disease, cancer) that could affect exercise outcomes will be excluded.
(2) Studies that include exercise but lack a clear aerobic exercise component, or studies that include aerobic exercise but do not specify its frequency, intensity, time, or type, will be excluded.
(3) RCTs that do not report the outcome measures required for this study, or studies with incomplete full text lacking the statistical data necessary to calculate effect sizes, will be excluded.
(4) Non-RCTs, such as reviews or case reports, will be excluded.

### 2.3 Data extraction and quality assessment

Data from the included RCTs were independently extracted by two reviewers in accordance with the Preferred Reporting Items for Systematic Reviews and Meta-Analyses (PRISMA) guidelines. Disagreements were adjudicated by consulting a third reviewer to ensure both accuracy and consistency. For every included trial, we extracted the following study- and participant-level characteristics: name of first author, publication year, sample size, age and sex distribution, body composition, time elapsed since stroke onset, side of hemiparesis, baseline Gait Speed, baseline VO_2_peak and details of the aerobic intervention including intensity, frequency and total duration.

The risk of bias for each included RCT was assessed using the Cochrane Risk of Bias Tool (Version 2.0) (33). This instrument evaluates methodological quality across five domains: (1) bias arising from the randomization process; (2) bias due to deviations from intended interventions; (3) bias due to missing outcome data; (4) bias in outcome measurement; and (5) bias in the selection of reported results. Each domain was rated as having a low risk of bias, some concerns, or a high risk of bias.

### 2.4 Grading of recommendations assessment, development, and evaluation

The certainty of evidence in this network meta-analysis was evaluated according to the GRADE framework using the CINeMA platform. RCTs were considered the highest initial level of evidence. The risk of bias for individual studies was assessed across domains using the RoB 2.0 tool and synthesized at the comparison level through weighted contributions within the CINeMA contribution matrix, yielding an overall judgment of within-study bias. Indirectness was assessed based on the assumptions of transitivity and exchangeability. Predefined effect modifiers (e.g., baseline disease severity, intervention intensity, and duration of follow-up) were examined to ensure consistency of populations, interventions, outcomes, and study settings between direct and indirect evidence. Imprecision was assessed by comparing 95% Confidence Interval (CI) against predefined minimally important differences (MIDs) to determine whether the CI crossed the line of no effect or clinically relevant decision thresholds; heterogeneity was assessed by evaluating the agreement between confidence intervals and prediction intervals in relation to the MID, supplemented by statistical indicators such as *τ*^2^ and *I*^2^; incoherence was examined in the presence of closed loops using CINeMA’s built-in consistency assessments, including the SIDE (Separate Indirect from Direct Evidence) approach and design-by-treatment interaction models, to evaluate the agreement between direct and indirect estimates. Across-study bias (e.g., reporting bias) was evaluated by triangulating evidence from trial registries, and small-study effects, using comparison-adjusted funnel plots and regression-based asymmetry tests. For each domain, the risk assessment was categorized as indicating either no concerns, some concerns, or major concerns. The overall certainty of the evidence was subsequently downgraded in accordance with these judgments, by one level when some concerns were identified and by two levels when major concerns were present. The final confidence in the evidence at the comparison level was then classified as high, moderate, low, or very low.

### 2.5 Statistical methods

Network meta-analysis was conducted using the network suite in Stata/MP 17.0. For continuous outcomes, effect sizes were expressed as mean differences (MDs). When outcomes were measured using different scales across studies, standardized mean differences (SMDs) were calculated to ensure comparability. All effect estimates are reported with 95% CI.

Network plots were generated to describe the geometry of the evidence. Node size was proportional to the total sample size receiving each intervention, and edge thickness reflected the number of direct head-to-head RCTs comparing the corresponding interventions. For networks without closed loops, a consistency model was applied by default. For networks containing closed loops, global inconsistency was assessed using the design-by-treatment interaction model (and/or the global Q statistic, as appropriate). Local inconsistency was evaluated using node-splitting. A *p*-value > 0.05 was considered to indicate no evidence of inconsistency between direct and indirect comparisons, supporting the use of a consistency model. In addition, loop-specific inconsistency was examined; if the 95% CI of the inconsistency factor included zero, consistency within that loop was assumed. Where inconsistency or substantial heterogeneity was detected, prespecified subgroup analyses and network meta-regression were conducted to explore potential sources and to assess the robustness of the findings. To rank the relative effectiveness of interventions, surface under the cumulative ranking curve (SUCRA) values were calculated, and cumulative ranking probability plots were produced. Potential publication bias and small-study effects were explored using comparison-adjusted funnel plots. All statistical tests were two-sided, and *p* < 0.05 was considered statistically significant.

Robustness of the main findings was further examined using a leave-one-out sensitivity analysis. Each included study was removed sequentially, the network was re-estimated using a random-effects NMA model under the consistency assumption, and pooled effects of each intervention versus SOC were recalculated. Iterative estimates were compared with the primary results to identify any reversal in effect direction or meaningful changes in magnitude or statistical significance. If omitting any single study did not materially alter the relative effects versus SOC, the network findings were considered robust.

We additionally performed a sensitivity analysis excluding studies that enrolled participants with a post-stroke duration of ≤6 months, to evaluate whether conclusions changed after removing these studies. Finally, we conducted study-level univariable network meta-regression to explore potential effect modification by candidate covariates. Each model included a single covariate as a predictor of the relative treatment effects. Random-effects structures were used to account for residual heterogeneity across studies, and the between-study variance (*τ*^2^) was estimated using the restricted maximum likelihood (REML) method. For each model, we report the regression coefficient with its 95% CI and the corresponding Wald test *p*-value. A two-sided *p* < 0.05 was considered evidence of a statistically significant association between the covariate and treatment effect.

## 3 Results

### 3.1 Characteristics of included studies

Detailed characteristics of the included studies are presented in Tables 1, 2.

**Table 1:** Baseline characteristics of studies included in the network meta-analysis.

First author (year)	Sample size		Age		Sex (male/female)		Body mass index (BMI)		Time since stroke onset (years)		Hemiparetic side (left/right/bilateral)	
	Intervention	Control	Intervention	Control	Intervention	Control	Intervention	Control	Intervention	Control	Intervention	Control
Boyne P. (2016) (16)	11	5	59 ± 9	57 ± 12	7/4	2/3	28.5 ± 5.2	26.4 ± 4.8	3.8 ± 2.9	6.3 ± 2.0	3/8	4/1
Boyne P. (2023) (87)	27	28	63.8 ± 9.9	61.5 ± 9.9	16/11	20/8	28.9 ± 5.3	28.7 ± 4.6	2.7 ± 1.4	2.2 ± 1.2	14/13	14/14
Hong J. (2013) (43)	65	63	57.6 ± 6.6	56.3 ± 6.5	46/19	45/18	25.6 ± 3.5	25.6 ± 3.6	1.6 ± 0.4	1.5 ± 0.4	33/32	37/26
Lapointe T. (2023) (88)	arm1:19 arm2:16	17	arm1:71.8 ± 9.9 arm2:65.6 ± 11.3	69.6 ± 10.7	arm1:13/6 arm2:10/6	10/7	arm1:27.9 ± 2.4 arm2:28.4 ± 5.4	28.3 ± 5.9	arm1:3.1 ± 5.1 arm2:4.3 ± 6.6	2.4 ± 3.3	Na	Na
Munari D. (2018) (89)	8	7	61 ± 5.77	62 ± 11.27	7/1	7/0	28.7 ± 3.3	25.8 ± 1.9	5.2 ± 2.9	6.4 ± 3.8	5/3	4/3
Tang A. (2014) (44)	25	25	65.9 ± 6.4	66.9 ± 7.8	14/11	15/10	28.0 ± 3.5	27.1 ± 5.7	4.3 ± 2.9	4.0 ± 3.0	15/10	16/8/1
Globas C. (2012) (54)	18	18	68.6 ± 6.7	68.7 ± 6.1	14/4	15/3	25.9 ± 3.4	26.8 ± 3.4	5 ± 3.9	5.8 ± 5.6	4/14	9/9
Gordon C. D. (2013) (34)	64	64	63.4 ± 9.4	64.9 ± 11.1	29/35	29/35	Na	Na	1.01 ± 0.3	1 ± 0.3	22/42	28/36
Hornby T. G. (2019) (90)	28	32	59 ± 9.5	56 ± 11.5	23/5	18/14	Na	Na	5 ± 10.4	2.3 ± 2.2	16/12	19/13
Ivey F. M. (2010) (35)	29	24	62 ± 8	60 ± 8	18/11	11/13	28.4 ± 6	26.1 ± 5	Na	Na	Na	Na
Ivey F. M. (2007) (36)	26	20	63 ± 9	62 ± 10	13/13	13/7	27 ± 4	29 ± 6	Na	Na	Na	Na
Lee M. J. (2008) (45)	arm1:12 arm2:12	12	arm1:62.9 ± 9.3 arm2:60.5 ± 10.6	65.3 ± 6.0	arm1:8/4 arm2:8/4	6/6	Na	Na	arm1:3.7 ± 5.3 arm2:5.3 ± 3.4	5.5 ± 3.5	arm1:5/7 arm2:5/7	6/6
Macko R. F. (2005) (37)	32	29	63 ± 10	64 ± 8	22/10	21/8	Na	Na	2.9 ± 2.4	3.3 ± 5	14/18	16/13
Quaney B. M. (2009) (38)	19	19	64.10 ± 12.30	58.96 ± 14.68	10/9	7/12	Na	Na	4.62 ± 3.21	5.11 ± 3.53	Na	Na
Serra M. C. (2019) (39)	17	8	58.1 ± 1.2	61.5 ± 1.3	14/3	6/2	33.4 ± 1.7	28.7 ± 1.6	Na	Na	Na	Na
Moore S. A. (2015) (40)	20	20	68 ± 8	70 ± 11	18/2	16/4	26 ± 4	26 ± 4	1.8 ± 2.8	1.3 ± 1	9/10/1	7/11/2
Kim J. (2017) (41)	14	15	50.7 ± 14.8	51.9 ± 17.4	9/5	10/5	24.5 ± 3.5	23.0 ± 4.3	1 ± 0.6	1 ± 0.7	Na	Na
Liu-Ambrose T. (2015) (42)	11	14	62.9 ± 12.1	66.9 ± 9.0	4/7	11/3	28.3 ± 7.0	26.9 ± 6.2	2.4 ± 1.0	2.9 ± 1.1	7/4	7/7
Yeh T. T. (2019) (55)	15	15	50.63 ± 3.99	60.21 ± 3.1	8/7	13/2	Na	Na	4 ± 1	7.9 ± 2.6	Na	Na
Yeh T. T. (2022) (91)	18	18	57.36 ± 12.17	60.17 ± 12.13	13/5	13/5	Na	Na	2.9 ± 2.6	4.2 ± 3.8	9/9	11/7
Doğan Duran Ü. (2023) (92)	13	13	54.1 ± 18.9	57.9 ± 10.9	Na	Na	29.2 ± 4.0	27.6 ± 3.1	1 ± 0.3	0.8 ± 0.4	Na	Na
Mustafaoğlu R. (2018) (46)	15	15	52.8 ± 13.8	52.6 ± 14.7	10/5	11/4	26.5 ± 3.6	26.5 ± 4.1	1.3 ± 0.7	1.1 ± 0.5	9/6	10/5
Kim S. J. (2015) (93)	16	16	65.2 ± 6.4	61.7 ± 6.1	12/4	13/3	25.5 ± 4.5	23.4 ± 3.9	Na	Na	9/7	6/10
Au-Yeung S. S. Y. (2009) (47)	59	55	61.7 ± 10.5	65.9 ± 10.7	33/26	33/22	24.7 ± 5.6	24.4 ± 5.2	4.5 ± 6.6	5.4 ± 8.9	31/28	25/30
Mberti N. L. A. (2017) (48)	17	18	67 ± 10	69 ± 9	13/4	14/4	26 ± 4	27 ± 5	3.3 ± 4.3	2.8 ± 3.8	Na	Na
Rimmer J. H. (2009) (49)	arm1:18 arm2:19	18	arm1:55.7 ± 12.6 arm2:59.4 ± 7.1	63.7 ± 9.1	arm1:6/12 arm2:8/11	8/10	arm1:33.8 ± 9.0 arm2:33.0 ± 8.2	28.9 ± 5.0	Na	Na	arm1: 10/7 arm2: 5/11	13/5
Thompson E. D. (2024) (53)	80	81	62 ± 1.46	62 ± 1.44	44/36	42/39	30.13 ± 0.61	31.58 ± 0.78	3.5 ± 0.5	3.8 ± 0.7	39/37/4	42/34/5
Moncion K. (2024) (56)	42	40	65.4 ± 8.9	64.4 ± 9.7	25/17	23/17	28.0 ± 5.2	29.2 ± 8.2	1.9 ± 1.3	1.7 ± 1.3	Na	Na
Linder S. M. (2024) (50)	25	25	60.1 ± 12.0	59.6 ± 9.6	15/10	14/11	28.3 ± 4.9	28.4 ± 5.8	2.4 ± 3.2	3 ± 3	Na	Na
Boyne P. (2025) (94)	27	28	63.8 ± 9.9	61.5 ± 9.9	16/11	20/8	28.9 ± 5.3	28.7 ± 4.6	2.77 ± 1.78	2.10 ± 1.33	Na	Na
Do J. (2024) (51)	22	22	59.22 ± 14.0	64.0 ± 10.2	16/6	11/11	22.1 ± 6.5	24.9 ± 4.8	2.8 ± 2.8	2.3 ± 1.4	Na	Na
Wu C. (2025) (95)	20	20	52.40 ± 14.45	56.20 ± 14.51	14/6	14/6	24.2 ± 5.3	24.3 ± 5.6	0.35 ± 0.3	0.4 ± 0.3	10/10	12/8
Palmcrantz S. (2021) (52)	16	15	62.25 ± 7.9	60.00 ± 7.29	11/5	13/2	Na	Na	1.8 ± 1.5	2.3 ± 3	10/6	9/6

**Table 2:** Summary of intervention and control conditions.

First author (year)	Rehabilitation intervention	
	Intervention group	Control group
Boyne P. (2016) (16)	Interval treadmill walking: 30-s bouts at maximal tolerable (safe) speed, interspersed with 30–60 s rest (treadmill stopped), for a total of 20 min/session; average HR increased from 53% HRR (week 1) to 72% HRR (week 4); RPE rose from 13.5 to 14.6.	Continuous treadmill walking: 20-min continuous walking/session, speed adjusted to maintain 45% ± 5% HRR in weeks 1–2, then 50% ± 5% HRR thereafter.
Boyne P. (2023) (87)	Interval fast-walking training: 30-s fast walking at maximal safe speed, alternated with 30–60 s standing/seated rest, at >60% HRR; each session included 10 min treadmill + 20 min overground + 10 min treadmill walking; 45 min/session, 3 sessions/week, for 12 weeks.	Continuous walking training: continuous walking at 40% ± 5% HRR initially, increased by 5% HRR every 2 weeks up to 60% HRR; 40 min/session.
Hong J. (2013) (43)	Cycle ergometer aerobic training: initiated at 40–50% HRR for 10–20 min, with ~5 min duration and 5% HRR intensity increase every 2 weeks until 50–70% HRR; 40 min/session, 5 sessions/week, for 12 weeks.	Conventional low-intensity activity: 35 min of supervised stretching exercises plus 5 min of low-intensity overground walking at 20–30% HRR.
Lapointe T. (2023) (88)	arm1: Week 1: 3 supervised MICT sessions (adaptation); Weeks 2–8: 3 supervised HIIT sessions/week; Weeks 9–16: 2 supervised HIIT + 1 home-based MICT/week; Weeks 17–24: 1 supervised HIIT + 2 home-based MICT/week. HIIT: upright cycle ergometer, high-intensity bouts at 95% PPO (GXT-derived), initially 30 s then extended to 60 s; recovery bouts fixed at 60 s—passive rest during month 1, then active recovery at 40% PPO; total 20–40 min/session (progressively increased as tolerated). MICT: 30 min moderate-intensity aerobic exercise (RPE 4–6/10) such as walking, swimming, dancing, or cycling, in bouts ≥10 min. arm2:1 supervised MICT session/week (cycle ergometer at 50% PPO) + 2 home-based MICT sessions/week (30 min at moderate intensity, RPE 4–6/10), with session duration progressively increased from 20 min to 40 min.	Standard physician health advice only, without additional exercise prescription or interaction with study staff.
Munari D. (2018) (89)	High-intensity interval treadmill training: 5 × 5-min bouts at 85–95% VO₂peak, primarily achieved by increasing treadmill incline (average 17%), interspersed with 3-min active walking recovery at ~50% VO₂peak; total 50–60 min/session, 3 sessions/week, for 3 months.	Continuous treadmill walking: 40-min walking at ~80% of self-selected speed, corresponding to ~60% VO₂peak.
Tang A. (2014) (44)	Combined aerobic and functional training: initiated at 40% HRR, increased by 10% every 4 weeks up to 70–80% HRR; 30–40 min/session of level and inclined treadmill walking, upright and recumbent cycling, and functional activities (e.g., marching in place, sit-to-stand, step-ups); 60 min/session, 3 sessions/week, for 6 months.	Low-intensity usual care: exercise intensity limited to <40% HRR, avoiding sustained aerobic loading, including stretching, light resistance, or balance exercises.
Globas C. (2012) (54)	Progressive treadmill training: session duration increased from 16.1 ± 4.4 min to 39.4 ± 8.6 min; walking speed progressed from 0.52 ± 0.22 m/s to 1.01 ± 0.38 m/s; average intensity increased from 48.9 ± 11.8% HRR to 76.7 ± 13.9% HRR over the 3-month program; 3 sessions/week.	Conventional rehabilitation: passive or tone-modulating exercises and balance training, ~60 min/session, without an aerobic component.
Gordon C. D. (2013) (34)	Home- or community-based brisk overground walking: starting at 15 min/session, increased by 5 min/week up to 30 min/session; target heart rate 60–85% HRmax; 3 sessions/week for 12 weeks	Light massage of the affected limb: 25 min/session, 3 sessions/week, for 12 weeks.
Hornby T. G. (2019) (90)	Task-oriented gait training: 3–5 sessions/week, 40 min/session, for 2 months; including treadmill walking (high-speed/skill-dependent), overground walking, multidirectional walking, stair climbing, and walking on unstable or obstacle-laden surfaces; target HR 70–80% HRR.	Gait training in variable environments: similar walking tasks as the intervention group but at lower intensity; target HR 30–40% HRR.
Ivey F. M. (2010) (35)	Progressive treadmill aerobic training: 6 months, 3 sessions/week, 40 min/session; initial intensity 40–50% HRR, progressively increased to 60–70% HRR.	Conventional stroke rehabilitation: 13 commonly used active or passive stretching exercises for upper and lower limbs, 30–40 min/session.
Ivey F. M. (2007) (36)	Progressive treadmill aerobic training: 6 months, 3 sessions/week, 40 min/session; initial intensity 40–50% HRR, progressively increased to 60–70% HRR; early sessions in an intermittent format with training duration increased by 5 min every 2 weeks.	Conventional stroke rehabilitation: 13 commonly used active or passive stretching exercises for upper and lower limbs, 30–40 min/session.
Lee M. J. (2008) (45)	arm1: Sham cycling for 30 min followed by PRT: resistance training using a Keiser pneumatic machine for lower-limb extensors, knee extensors/flexors, and ankle plantar−/dorsiflexors; hip abductors and ankle dorsiflexors trained with free weights or isometric exercises; unilateral, 2 sets × 8 reps per exercise; load started at 50% 1RM and increased to 80% 1RM within 2 weeks. arm2: Recumbent motor-driven isokinetic cycling: initial aerobic cycling at 50% VO₂peak, increased to 70% after 2 weeks, followed by 30 min of the same PRT protocol as intervention group 1.	Control intervention: 30 min of sham cycling (passive motor-driven cycling) followed by sham PRT (only overcoming the device’s inertia without applied resistance).
Macko R. F. (2005) (37)	Progressive treadmill aerobic training: 3 sessions/week; initial intensity 40–50% HRR with 10–20 min/session; duration increased by ~5 min every 2 weeks until 40 min; target intensity increased by ~5% HRR every 2 weeks, reaching 60–70% HRR	Conventional rehabilitation: 13 commonly used stretching and flexibility exercises for upper and lower limbs (~35 min) plus low-intensity treadmill walking (~5 min) at 30–40% HRR.
Quaney B. M. (2009) (38)	Stationary cycle aerobic training: 8 weeks, 3 sessions/week; week 1 tolerance training at 40–50% HRmax for 10–20 min; from week 2 intensity progressively increased to 70% HRmax; thereafter 45 min/session.	Conventional rehabilitation: stretching exercises for the upper and lower limbs without an aerobic component.
Serra M. C. (2019) (39)	Treadmill walking training: 24 weeks, 3 sessions/week; initial duration 15 min at 40–50% HRR, progressively increased weekly to 50 min/session with intensity raised to 60–70% HRR.	Whole-body stretching and balance training: 2 sessions/week, 50 min/session.
Moore S. A. (2015) (40)	Structured community-based exercise intervention: including stretching, strength, balance, and aerobic fitness training; 45–60 min/session, 3 sessions/week, for 19 weeks; initial intensity 40–50% HRmax, increased by ~10% every 4 weeks to reach 70–80% HRmax.	Static stretching exercises for major muscle groups.
Kim J. (2017) (41)	Conventional neurodevelopmental therapy (NDT) plus handgrip training (15 min/session) and treadmill training (15 min/session) with a 5% body-weight sandbag attached to the unaffected limb; 5 sessions/week for 6 weeks.	Conventional neurodevelopmental therapy (NDT).
Liu-Ambrose T. (2015) (42)	Combined exercise intervention: 2 sessions/week of exercise training (including resistance, balance, and aerobic training) plus 1 session/week of recreational activity; 60 min/session; for 6 months.	Received usual care
Yeh T. T. (2019) (55)	30 min stationary cycle aerobic training + 30 min cognitive training; aerobic exercise at a target HR of 40–70% HRmax; 2–3 sessions/week, 60 min/session, for 12–18 weeks, totaling 36 sessions.	30 min flexibility, strength, and balance training + 30 min non-specific cognitive activities; 2–3 sessions/week, 60 min/session, for 12–18 weeks, totaling 36 sessions.
Yeh T. T. (2022) (91)	Progressive resistance stationary cycling: initial intensity 40–50% HRmax, progressively increased to 60–70% HRmax; 3 sessions/week, 60 min/session, for 12 weeks.	Computer-based cognitive training using the BrainHQ software.
Doğan Duran Ü. (2023) (92)	Antigravity treadmill training: 3 sessions/week for a total of 12 sessions; 45 min/session at 2.0 mph, with 65% body-weight support and 0% incline.	Conventional rehabilitation therapy without additional aerobic training.
Mustafaoğlu R. (2018) (46)	Body-weight–supported treadmill training: initial support 30–40%, reduced by ~10% each session; treadmill speed 1.2–2.6 km/h, adjusted to maximal tolerable speed; 45 min/session, 2 sessions/week, plus 5 sessions/week of conventional rehabilitation.	Conventional rehabilitation: trunk stabilization, affected-side weight-shift training, parallel-bar or overground walking, coordinated upper-limb movements with multidirectional stepping, and verbal/tactile cues to promote symmetrical weight bearing; 45 min/session, 5 sessions/week.
Kim S. J. (2015) (93)	Stationary cycle training: 30 min/session, 5 sessions/week, for 4 weeks.	Conventional rehabilitation therapy.
Au-Yeung S. S. Y. (2009) (47)	Short-form Tai Chi: 4 h/week for 12 weeks	Breathing and stretching exercises, active range-of-motion exercises for the limbs and trunk, and seated and walking practice: 4 h/week.
Mberti N. L. A. (2017) (48)	Walking training: starting at 40% HRR and progressively increased to 60–70% HRR, maintained for ≥20 min within each session after an initial 5-min low-intensity walking phase; 3 sessions/week for 8 weeks.	Ground-based intermittent walking: 2 bouts of 10-min walking per session with an average training intensity of ~36% HRR.
Rimmer J. H. (2009) (49)	arm1: Aerobic training on a stationary cycle or recumbent stepper: 30 min/session with a 2-week adaptation period, progressively increasing intensity from 40% HRR to 70% HRR.arm2: Low-intensity aerobic training: intensity maintained at <50% HRR; 30 min/session during weeks 3–6, 45 min/session during weeks 7–10, and 60 min/session during weeks 11–14; 3 sessions/week.	Conventional rehabilitation exercises: including gait, balance, muscle-strengthening, and range-of-motion training; 60 min/session.
Thompson E. D. (2024) (53)	Combined walking training: ~30 min treadmill walking followed by 10 min overground walking per session, with step-count monitoring.	Behavioral intervention: step-count tracking with Fitbit plus goal setting and motivational interviewing–based counseling.
Moncion K. (2024) (56)	Adaptive recumbent stepper HIIT: 10 × 1-min high-intensity bouts (initially 80% HRR, RPE 14–17/20, increased by 10% every 4 weeks up to 100% HRR) interspersed with 9 × 1-min low-intensity recovery bouts (30% HRR); total core training duration 19 min; 3 sessions/week for 12 weeks.	Adaptive recumbent stepper MICT: initially 20 min at 40% HRR (RPE 9–11), increased by 10% HRR and extended by 5 min every 4 weeks to 30 min at 60% HRR (RPE 13–14); 3 sessions/week for 12 weeks.
Linder S. M. (2024) (50)	Motor-assisted recumbent cycle training plus RTP: 35 min of aerobic exercise at 60–80% HRR followed by 45 min of RTP for the upper limb.	RTP alone: 90 min/session without a systematic aerobic component.
Boyne P. (2025) (94)	Short-duration high-speed interval walking: 30-s bouts at maximal safe tolerable speed alternated with 30–60-s passive recovery; average training HR > 60% HRR.	Traditional moderate-intensity continuous walking: intensity maintained at 40–60% HRR.
Do J. (2024) (51)	End-effector robotic gait training: alternating 2 min of moderate-intensity walking (RPE 10–12) with 3 min of high-intensity walking (RPE > 14); 30 min/session, 3 sessions/week, for 8 weeks.	Conventional treadmill walking rehabilitation: progressive increase in walking speed with a target intensity of Borg RPE 13–15.
Wu C. (2025) (95)	Traditional step training based on the FITT principle: Borg RPE 6–9, 15 min/session, 5 sessions/week, for 4 weeks, in addition to the same conventional rehabilitation as the control group.	Conventional rehabilitation: comprehensive limb training, balance and weight-shift training, and ADL training; 2 sessions/day, 40 min/session, 5 days/week, for 4 weeks.
Palmcrantz S. (2021) (52)	Treadmill walking with body-weight support: 3 sessions/week, ≤1 h/session, including ≤30 min of conventional walking and mobility training.	No intervention.

**Figure 1:**
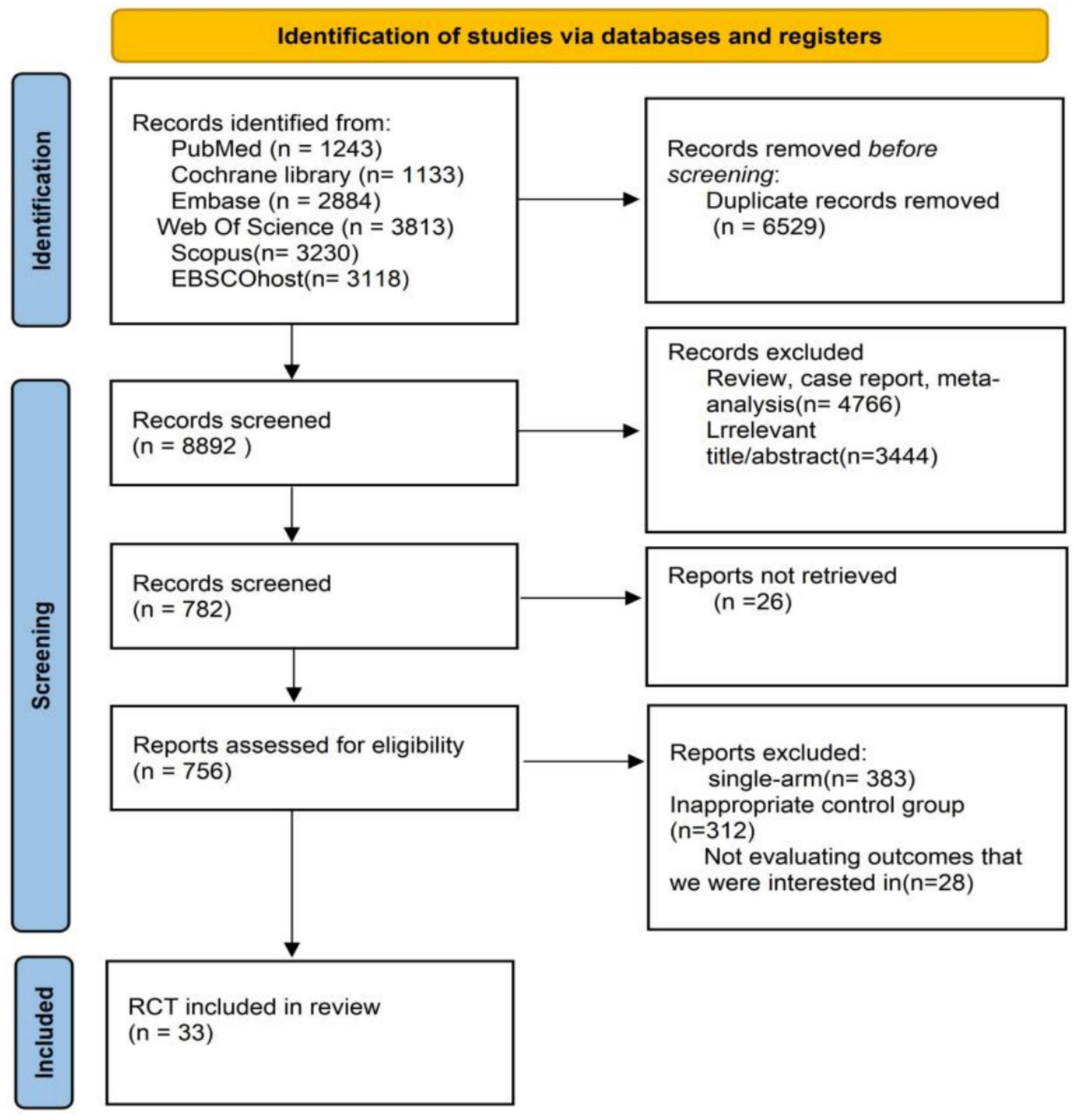
PRISMA 2020 flow diagram of study selection.

A total of 15,421 records were initially identified through database searches. Following removal of duplicates and screening of titles and abstracts, 8,892 records were retained for full-text evaluation. Of these, 33 studies satisfied all eligibility criteria and were included in the quantitative synthesis (Figure 1). In total, 1,665 participants were randomized into six categories of aerobic exercise conditions defined by training intensity: HIIT (6 arms), HICT (11 arms), MICT (19 arms), LICT (9 arms), Non-training control (4 arms), and SOC (20 arms). SOC protocols typically consisted of active or passive stretching, low-load resistance exercise, balance training, or gait practice.

In summary, individual trials enrolled between 5 and 81 participants, and the mean age of enrolled samples ranged from 50.6 to 70 years. Across all studies combined, 633 women were included, accounting for roughly 38 percent of the total cohort.

The methodological quality of the included studies was evaluated using the Cochrane RoB 2.0 tool. Among the 33 trials, seven were judged to have an overall low risk of bias, whereas the remaining 26 were rated as having some concerns (see Supplementary Figure 1). Several studies did not provide adequate information on allocation concealment or baseline comparability between groups during randomization, particularly in small-sample pilot trials where transparency of the randomization procedures was limited (16, 34–42). Given the nature of exercise interventions, blinding of participants and personnel was inherently difficult. In some studies, deviations from the intended intervention protocols may have occurred during implementation, potentially increasing the risk of performance bias (16, 34, 35, 43–52). With respect to missing outcome data, several studies reported participant attrition during follow-up or did not adequately explain the reasons for dropout, introducing uncertainty regarding the robustness of the final outcome estimates (16, 35–37, 39, 43, 47, 50, 52, 53). Although most studies employed objective outcome measures, such as the 6MWT, 10MWT, and VO_2_peak to evaluate efficacy, several did not clearly report whether outcome assessors were blinded, raising concerns about a potential risk of detection bias (41, 48, 51, 54, 55). In addition, several studies did not publicly register a study protocol or prespecified statistical analysis plan, which may have increased the risk of selective outcome reporting (16, 34, 36, 43, 46, 49).

### 3.2 Network meta-analyses

#### 3.2.1 Network geometry plot

**Figure 2:**
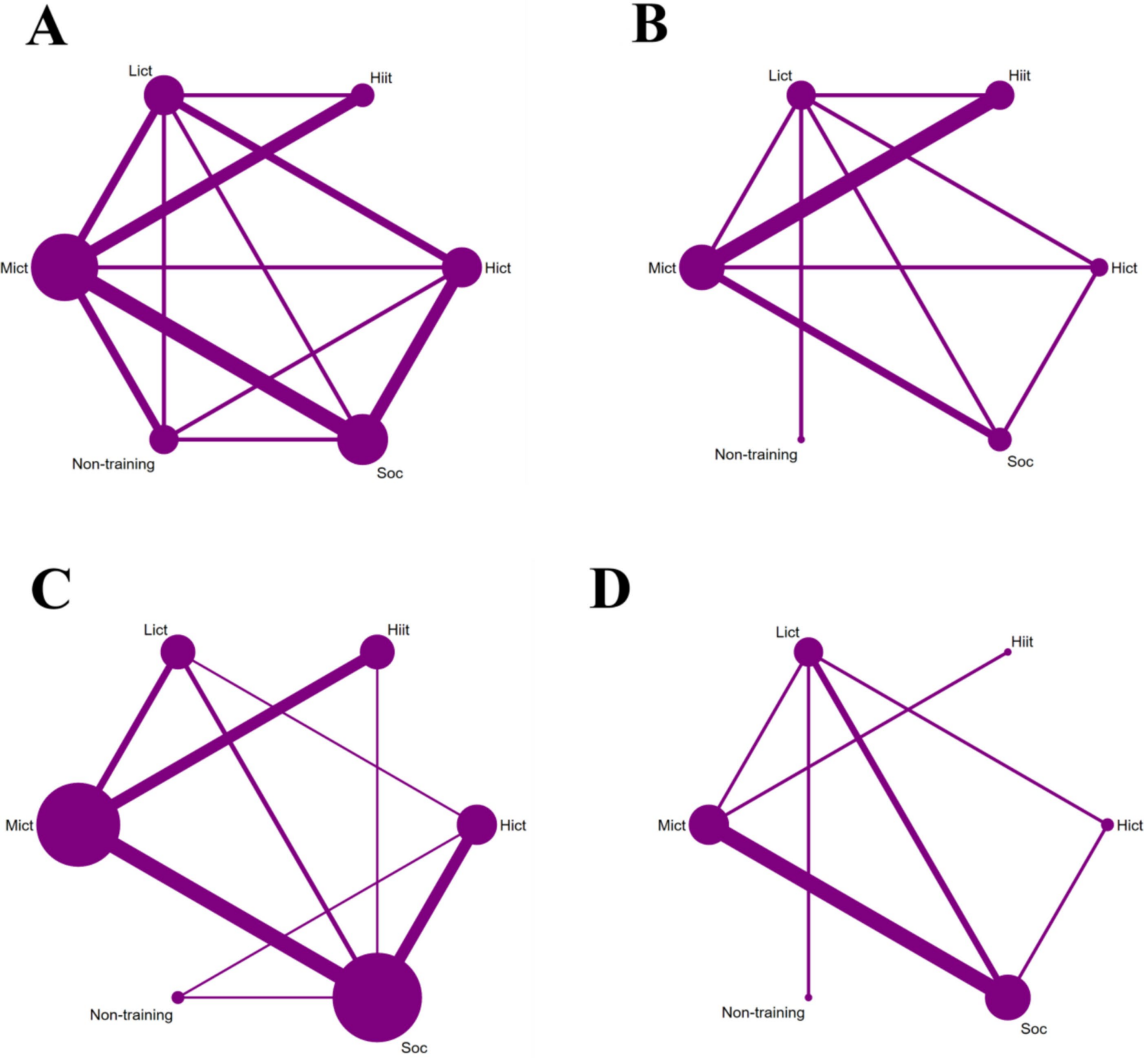
Evidence network for 6MWT **(A)** and 10MWT **(B)**. Evidence network for VO_2_peak **(C)** and BBS **(D)**.

**Figure 3:**
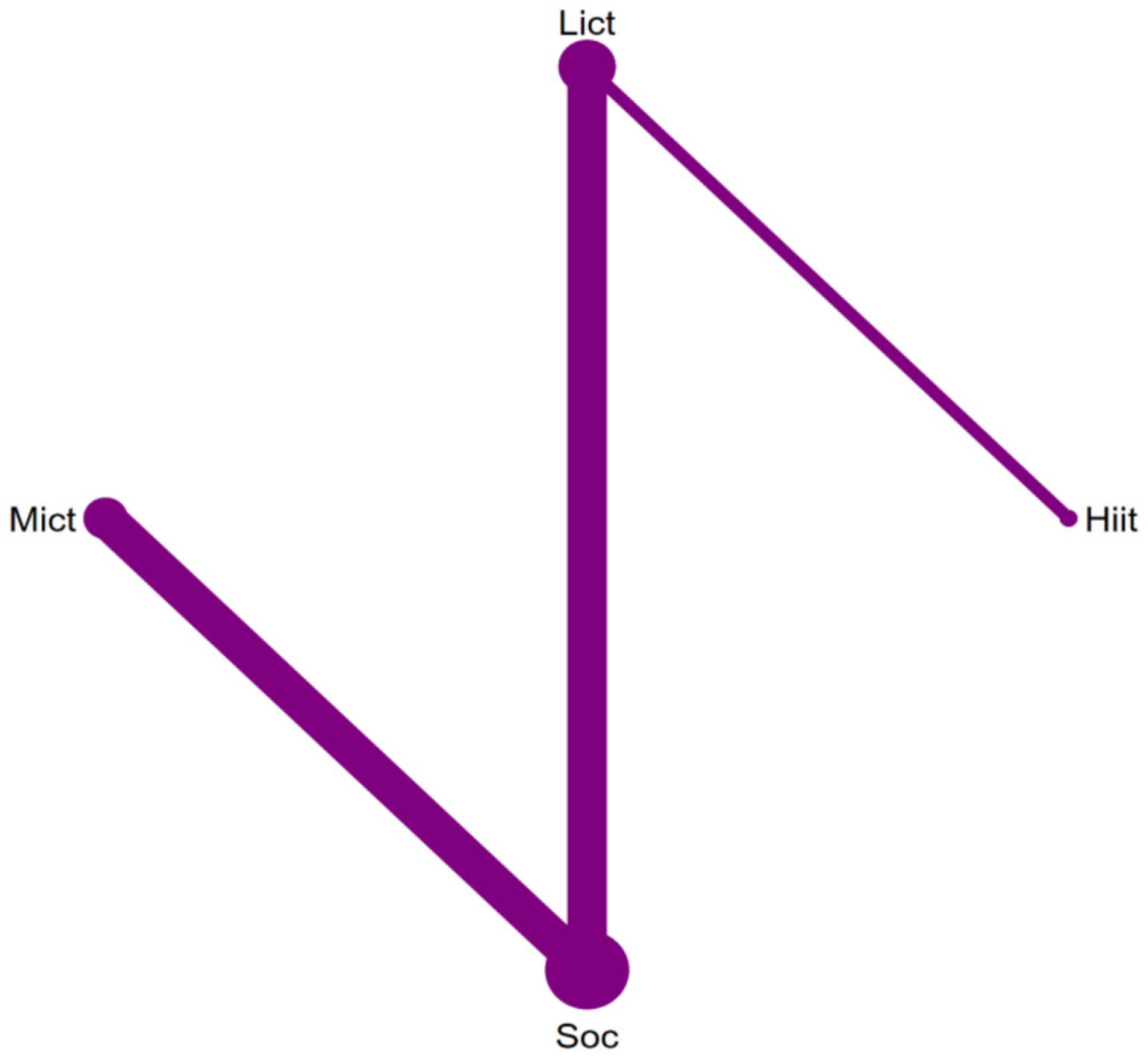
Evidence network for TUG. Each node represents an aerobic exercise intervention and is weighted based on the number of participants who underwent the intervention. The lines connecting the nodes are weighted according to the number of studies that include pairwise comparisons.

Figure 2 panels A through D, and Figure 3 illustrate the geometry of the treatment networks for the primary outcomes: 6MWT (A), 10MWT (B), VO_2_peak (C), BBS (D) and TUG. The network for the 6MWT comprised 20 RCTs with a total sample of 1,113 participants distributed among six intervention nodes. For gait speed assessed by the 10MWT, 13 studies including 566 individuals informed a network of six treatment options. With respect to VO_2_peak, 20 trials involving 1,040 participants contributed comparisons across six intervention groups. The BBS network was based on 12 studies including 504 participants and six intervention groups. The TUG outcome was informed by seven studies (*n* = 297) comparing four different interventions.

#### 3.2.2 6MWT

For the 6MWT, the network contained one or more closed loops, enabling assessment of consistency. Global inconsistency was examined using the design-by-treatment interaction model, which yielded a non-significant result (*p* = 0.9922 > 0.05), indicating no evidence of inconsistency across the network. Local inconsistency was further assessed through node-splitting analysis, with all corresponding *p*-values exceeding 0.05, supporting agreement between direct and indirect comparisons. Loop-specific inconsistency tests showed that the 95% CI of inconsistency factors included zero, with small point estimates, suggesting good homogeneity and coherence within the network. Accordingly, a consistency model was adopted for effect estimation (see Supplementary Figure 3). Compared with the SOC group, significant improvements in 6MWT were observed in the HIIT group (MD = 63.04, 95% CI [14.38, 111.69]) and the MICT group (MD = 39.23, 95% CI [4.13, 74.34]). Although the HICT group also showed improvement (MD = 27.93, 95% CI [−7.39, 63.25]), the difference did not reach statistical significance. In contrast, participants in the SOC and LICT groups demonstrated comparable changes in 6MWT (MD = 4.58, 95% CI [−36.16, 45.32]).

#### 3.2.3 10MWT

**Figure 4:**
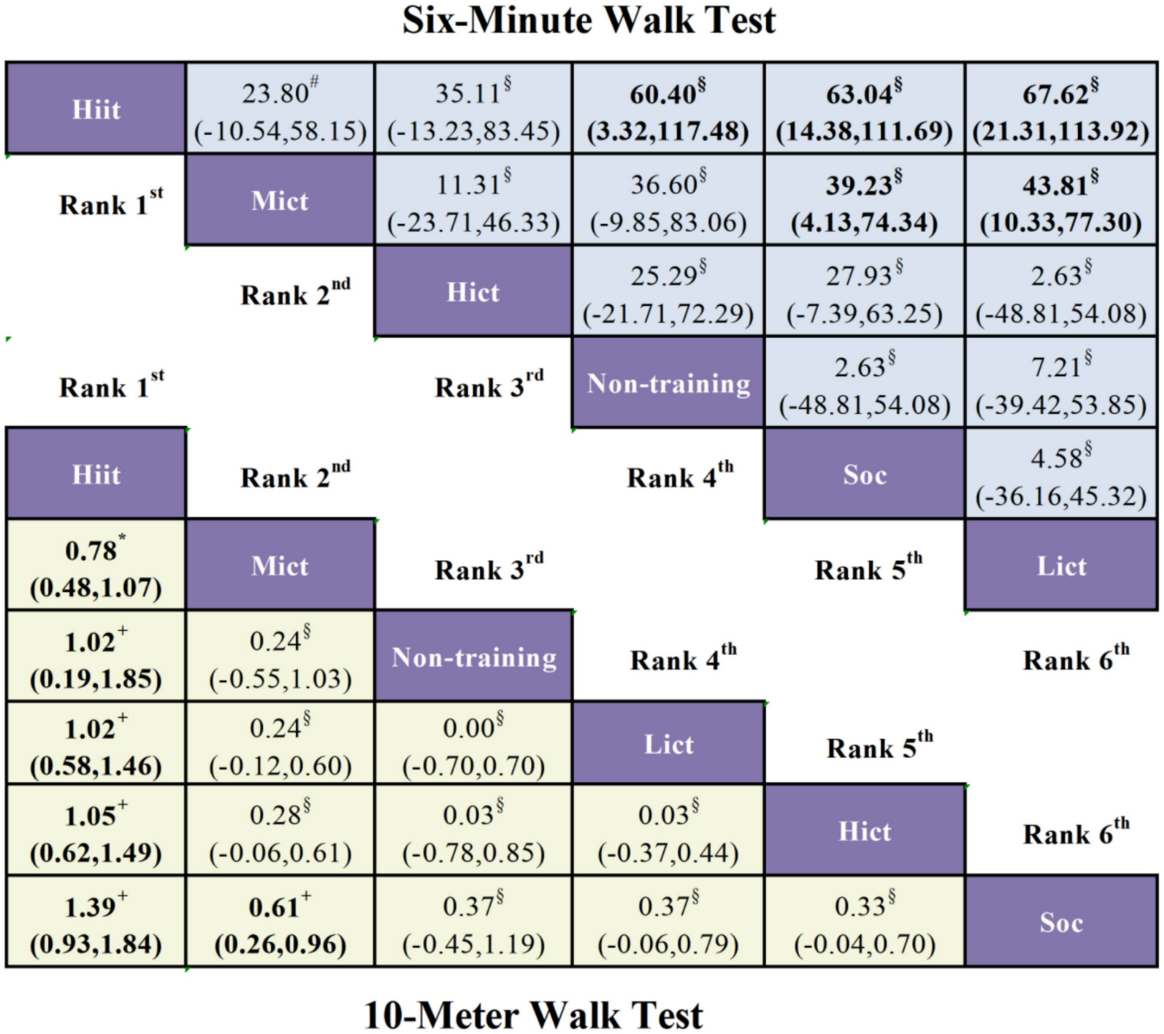
League tables of treatment effects from the network meta-analysis across aerobic training intensities in chronic stroke: 6MWT (upper) and 10MWT (lower). Data are displayed as posterior mean difference (95% CI). Values in bold indicate statistically significant results. Symbol legend for GRADE certainty of evidence: * = high; + = moderate; # = low; § = very low.

For the 10MWT, the network structure formed closed loops, permitting formal inconsistency testing. Global inconsistency assessed via the design-by-treatment interaction model was not statistically significant (*p* = 0.8657 > 0.05). Local inconsistency was examined using node-splitting, and all *p*-values were >0.05, indicating concordance between direct and indirect estimates. Loop-specific inconsistency tests showed that the 95% Cl for inconsistency factors contained zero, with small point estimates, suggesting good homogeneity within the network. Consequently, a consistency model was used for subsequent analysis (see Supplementary Figure 4). Compared with the SOC group, significant improvements in 10MWT performance were observed in both the HIIT group (SMD = 1.39, 95% CI [0.93, 1.84]) and the MICT group (SMD = 0.61, 95% CI [0.26, 0.96]). Although slight improvements were noted in the LICT group (SMD = 0.37, 95% CI [−0.06, 0.79]) and the HICT group (SMD = 0.33, 95% CI [−0.04, 0.70]), these did not reach statistical significance. When compared directly with the LICT group, only the HIIT group (SMD = 1.02, 95% CI [0.58, 1.46]) achieved a statistically significant improvement in walking speed over 10 meters (Figure 4).

#### 3.2.4 VO2peak

For the outcome of VO_2_peak, the network structure contained closed loops, allowing for the assessment of inconsistency. The global inconsistency test using the design-by-treatment interaction model yielded a non-significant result (*p* = 0.9277 > 0.05), indicating no evidence of inconsistency across the network. Local inconsistency, examined through node-splitting analysis, also produced *p*-values greater than 0.05, confirming agreement between direct and indirect evidence. Loop-specific inconsistency analysis demonstrated that the 95% Cl for inconsistency factors included zero, with small point estimates, suggesting good homogeneity within the network. Consequently, a consistency model was employed for the estimation of treatment effects (see Supplementary Figure 2). Using the Non-training group as the reference, both the HIIT group (MD = 6.18, 95% CI [1.80, 10.57]) and the MICT group (MD = 4.15, 95% CI [0.21, 8.08]) showed statistically significant improvements in VO_2_peak. Moreover, compared with the SOC group, VO_2_peak significantly increased in the HIIT group (MD = 5.50, 95% CI [3.15, 7.86]), the MICT group (MD = 3.47, 95% CI [2.13, 4.80]), and the HICT group (MD = 1.82, 95% CI [0.11, 3.52]; Table 3).

**Table 3:** Baseline VO_2peak_, gait speed, and Intervene in the single time, frequency and total duration.

First author (year)	VO_₂peak_ (mL/kg/min)		Gait speed (m/s)		Intervene in the single time, frequency and total duration
	Intervention	Control	Intervention	Control	
Boyne P. (2016) (16)	16 ± 4	21.6 ± 4	0.63 ± 0.48	0.76 ± 0.36	20 min/session, 3 sessions/week, for 4 weeks.
Boyne P. (2023) (87)	14.3 ± 4.4	14 ± 4.8	0.65 ± 0.29	0.62 ± 0.33	45 min/session, 3 sessions/week, for 12 weeks.
Hong J. (2013) (43)	13.2 ± 0.9	13.2 ± 0.9	Na	Na	40 min/session, 5 sessions/week, for 12 weeks.
Lapointe T. (2023) (88)	arm1:18.9 ± 5.5 arm2:21.1 ± 4.5	18.7 ± 4.5	Na	Na	35 min/session, 3 sessions/week, for 12 weeks.
Munari D. (2018) (89)	20.88 ± 5.28	20.49 ± 5.58	0.91 ± 0.4	0.83 ± 0.4	55 min/session, 3 sessions/week, for 12 weeks.
Tang A. (2014) (44)	16.9 ± 7.1	16.9 ± 7.1	Na	Na	60 min/session, 3 sessions/week, for 24 weeks.
Globas C. (2012) (54)	18.9 ± 5	21.7 ± 5	0.91 ± 0.34	0.88 ± 0.56	40 min/session, 3 sessions/week, for 12 weeks.
Gordon C. D. (2013) (34)	Na	Na	Na	Na	25 min/session, 3 sessions/week, for 12 weeks.
Hornby T. G. (2019) (90)	14 ± 3.8	14 ± 3.8	0.52 ± 0.46	0.52 ± 0.46	40 min/session, 4 sessions/week, for 8 weeks.
Ivey F. M. (2010) (35)	14.1 ± 4.0	13.5 ± 3.6	0.58 ± 0.5	0.58 ± 0.5	40 min/session, 3 sessions/week, for 24 weeks.
Ivey F. M. (2007) (36)	13.7 ± 0.9	14.8 ± 0.9	Na	Na	40 min/session, 3 sessions/week, for 24 weeks.
Lee M. J. (2008) (45)	14.0 ± 3.3	14.4 ± 3.1	arm1:0.74 ± 0.3 arm2:0.71 ± 0.42	0.65 ± 0.36	30 min/session, 3 sessions/week, for 11 weeks.
Macko R. F. (2005) (37)	14.9 ± 0.9	14.9 ± 0.9	0.82 ± 0.2	0.90 ± 0.10	40 min/session, 3 sessions/week, for 24 weeks.
Quaney B. M. (2009) (38)	14.76 ± 4.23	14.76 ± 4.23	Na	Na	45 min/session,3 sessions/week, for 8 weeks
Serra M. C. (2019) (39)	16.9 ± 1.1	16.9 ± 1.1	Na	Na	35 min/session, 3 sessions/week, for 24 weeks
Moore S. A. (2015) (40)	18 ± 5	18 ± 5	1.2 ± 0.3	1.2 ± 0.3	45–60 min/session, 3 sessions/week, for 19 weeks.
Kim J. (2017) (41)	Na	Na	0.26 ± 0.3	0.22 ± 0.12	30 min/session, 5 sessions/week, for 6 weeks.
Liu-Ambrose T. (2015) (42)	Na	Na	Na	Na	60 min/session, 3 session/week, for 24 weeks.
Yeh T. T. (2019) (55)	Na	Na	Na	Na	60 min/session, 3 sessions/week, for 12–18 weeks, totaling 36 sessions.
Yeh T. T. (2022) (91)	Na	Na	Na	Na	60 min/session, 3 sessions/week, for 12 weeks.
Doğan Duran Ü. (2023) (92)	19.9 ± 4.5	19.9 ± 4.5	Na	Na	45 min/session, 3 sessions/week for 12 weeks.
Mustafaoğlu R. (2018) (46)	Na	Na	0.68 ± 0.1	0.5 ± 0.6	45 min/session, 2 sessions/week for 6 weeks.
Kim S. J. (2015) (93)	Na	Na	0.26 ± 0.15	0.23 ± 0.12	30 min/session, 5 sessions/week, for 4 weeks.
Au-Yeung S. S. Y. (2009) (47)	Na	Na	Na	Na	240 min/week for 12 weeks.
Mberti N. L. A. (2017) (48)	Na	Na	0.98 ± 0.41	0.98 ± 0.41	20 min/session, 3 sessions/week, for 8 weeks.
Rimmer J. H. (2009) (49)	15.06 ± 7.4	13.27 ± 3.6	Na	Na	30 min/session 3 sessions/week, for 14 weeks.
Thompson E. D. (2024) (53)	Na	Na	0.68 ± 0.02	0.68 ± 0.02	30 min/session, 3 sessions/week, for 12 weeks.
Moncion K. (2024) (56)	17.3 ± 5.9	17.2 ± 6.0	1.07 ± 0.35	1.05 ± 0.42	20 min/session; 3 sessions/week for 12 weeks.
Linder S. M. (2024) (50)	16.7 ± 5.6	16.7 ± 5.6	Na	Na	45 min/session, 3 sessions/week, for 8 weeks.
Boyne P. (2025) (94)	14.3 ± 4.4	14 ± 4.8	0.65 ± 0.29	0.62 ± 0.33	45 min/session, 3 sessions/week, for 12 weeks.
Do J. (2024) (51)	17.97 ± 11.39	14.65 ± 5.62	0.50 ± 0.22	0.41 ± 0.23	30 min/session, 3 sessions/week, for 8 weeks.
Wu C. (2025) (95)	Na	Na	Na	Na	15 min/session, 5 sessions/week, for 4 weeks.
Palmcrantz S. (2021) (52)	Na	Na	0.27 ± 0.16	0.33 ± 0.22	60 min/session, 3 sessions/week, for 6 weeks.

#### 3.2.5 BBS

**Figure 5:**
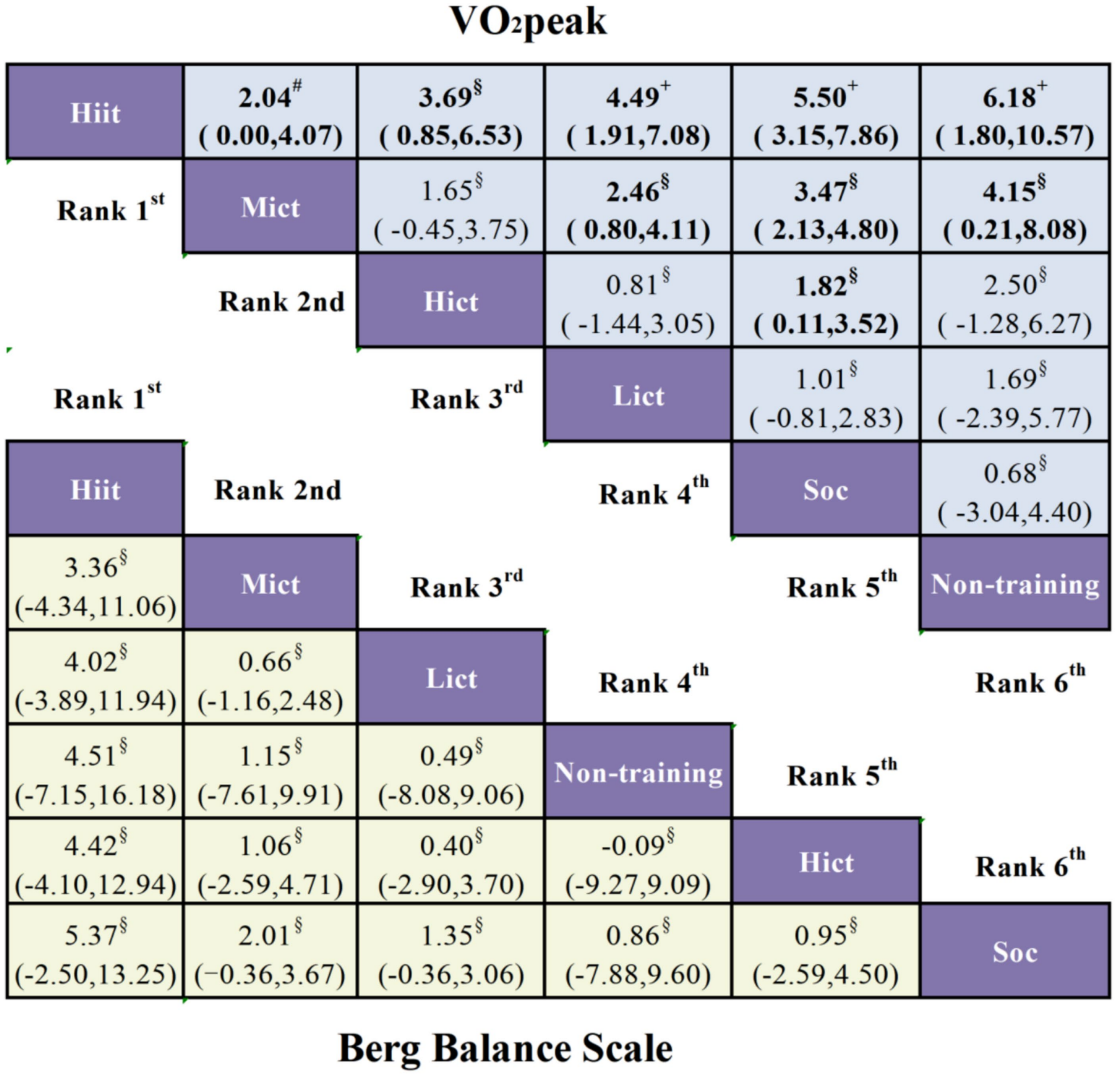
League tables of treatment effects from the network meta-analysis across aerobic training intensities in chronic stroke: VO_2_peak (upper) and BBS (lower). Data are displayed as posterior mean difference (95% CI). Values in bold indicate statistically significant results. Symbol legend for GRADE certainty of evidence: * = high; + = moderate; # = low; § = very low.

For the BBS, the network structure included closed loops, allowing assessment of both global and local inconsistency. The global inconsistency test based on the design-by-treatment interaction model revealed no significant inconsistency (*p* = 0.5917 > 0.05). Similarly, local inconsistency evaluated using the node-splitting method indicated that all *p*-values exceeded 0.05, demonstrating good agreement between direct and indirect evidence. Loop-specific inconsistency analysis further showed that the 95% Cl for inconsistency factors contained zero, with small point estimates, suggesting good homogeneity across comparisons. Therefore, a consistency model was adopted for subsequent analysis (see Supplementary Figure 5). Compared with the SOC group, only the MICT group showed a statistically significant improvement in BBS scores (MD = 2.01, 95% CI [0.36, 3.67]). Although both the HIIT group (MD = 5.37, 95% CI [−2.50, 13.25]) and the LICT group (MD = 1.35, 95% CI [−0.36, 3.06]) demonstrated numerical improvements, these changes did not reach statistical significance. The HICT group (MD = 0.95, 95% CI [−2.59, 4.50]) showed performance comparable to that of the SOC group, with no significant differences observed (Figure 5).

#### 3.2.6 Timed up and go

**Figure 6:**
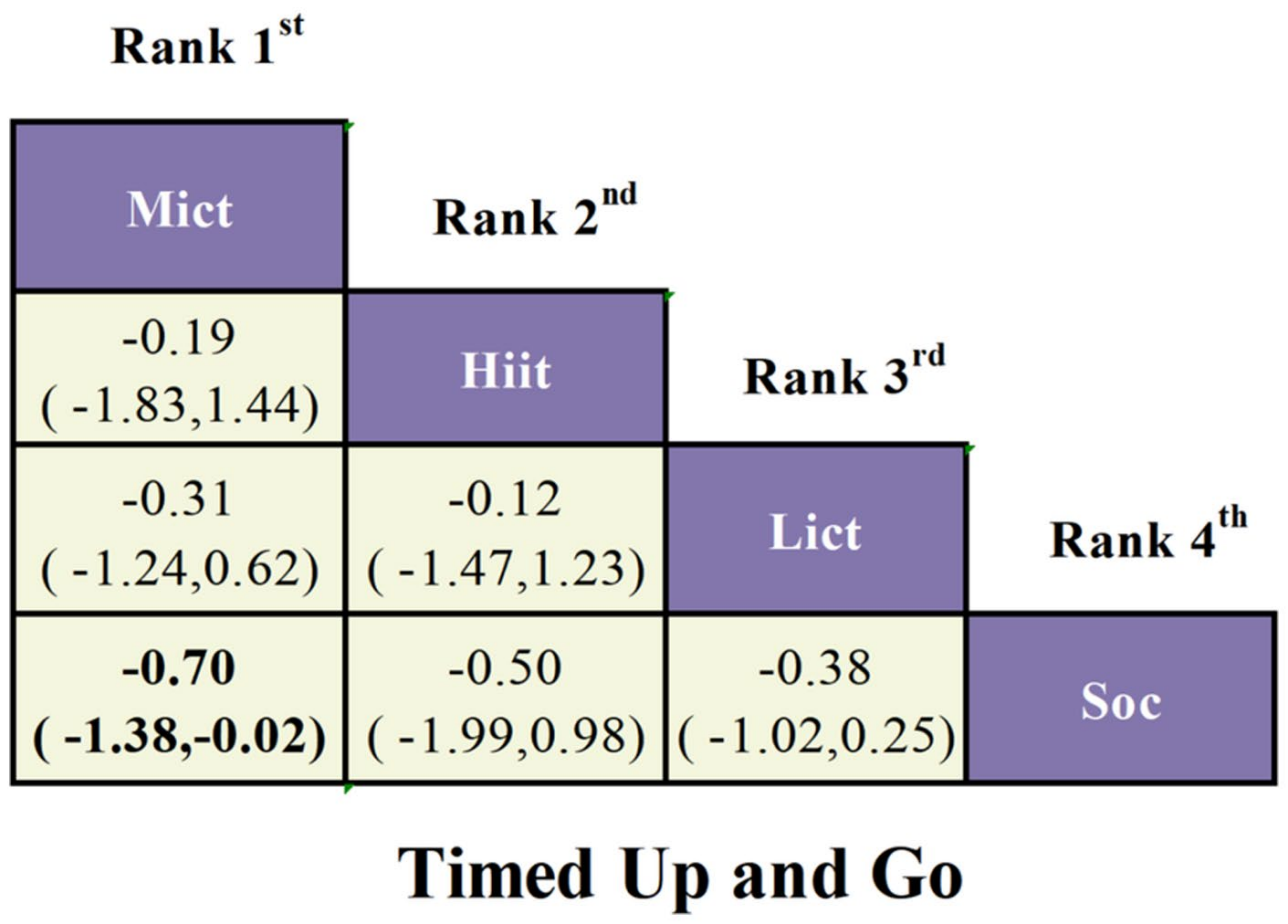
League tables of treatment effects from the network meta-analysis across aerobic training intensities in chronic stroke: TUG. Data are displayed as posterior mean difference (95% CI). Values in bold indicate statistically significant results.

For the TUG, the network geometry did not form any closed loops; therefore, a consistency model was directly applied for analysis. Compared with the Soc group, only the MICT group demonstrated a statistically significant reduction in TUG time (MD = −0.70, 95% CI [−1.38, −0.02]). Although both the HIIT group (MD = −0.50, 95% CI [−1.99, 0.98]) and the LICT group (MD = −0.38, 95% CI [−1.02, 0.25]) showed slight decreases in TUG, these differences did not reach statistical significance (Figure 6).

### 3.3 Rank

**Figure 7:**
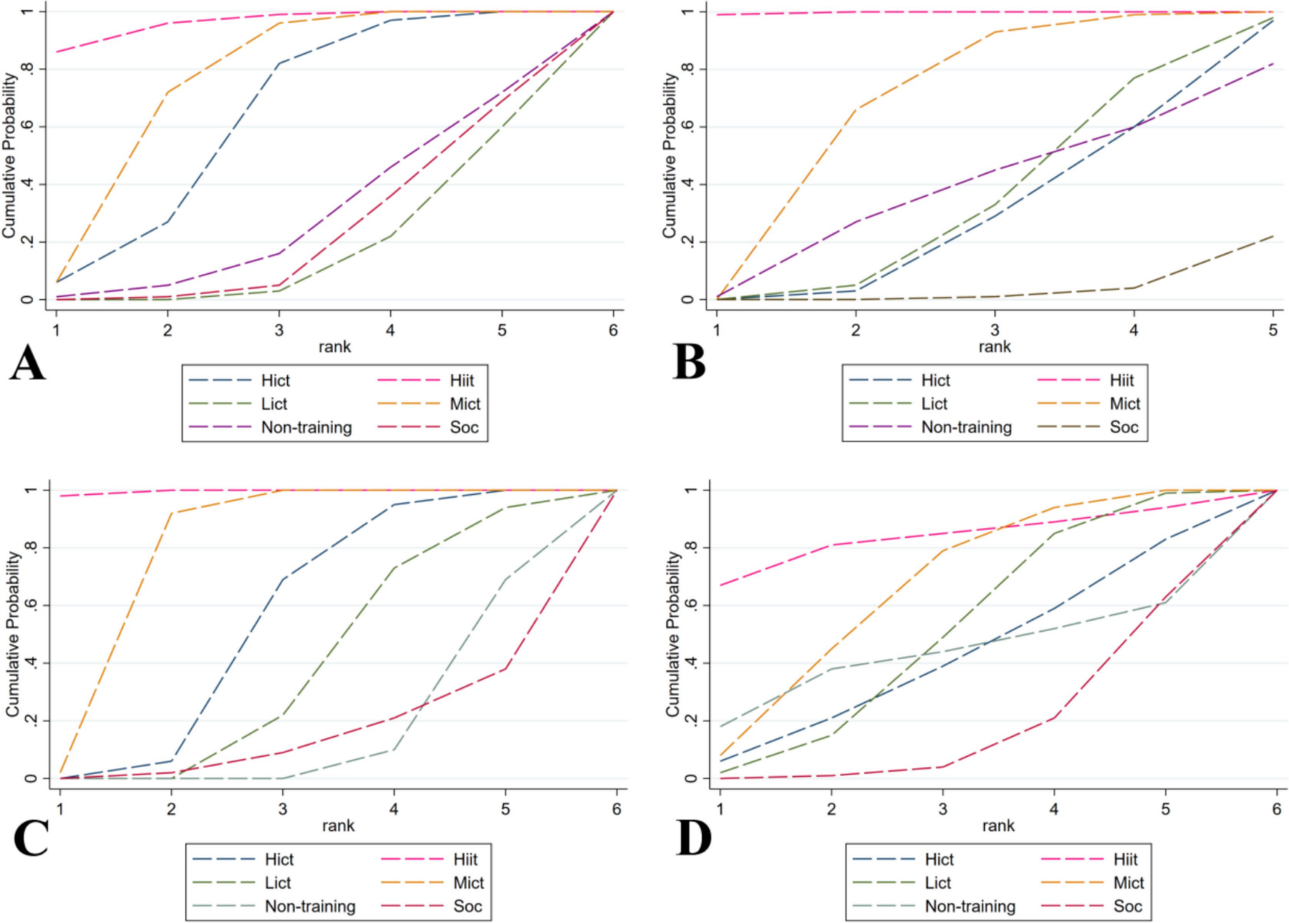
Summary rank plots for 6MWT **(A)** and 10MWT **(B)**. Summary rank plots for VO_2_peak **(C)** and BBS **(D)**.

**Figure 8:**
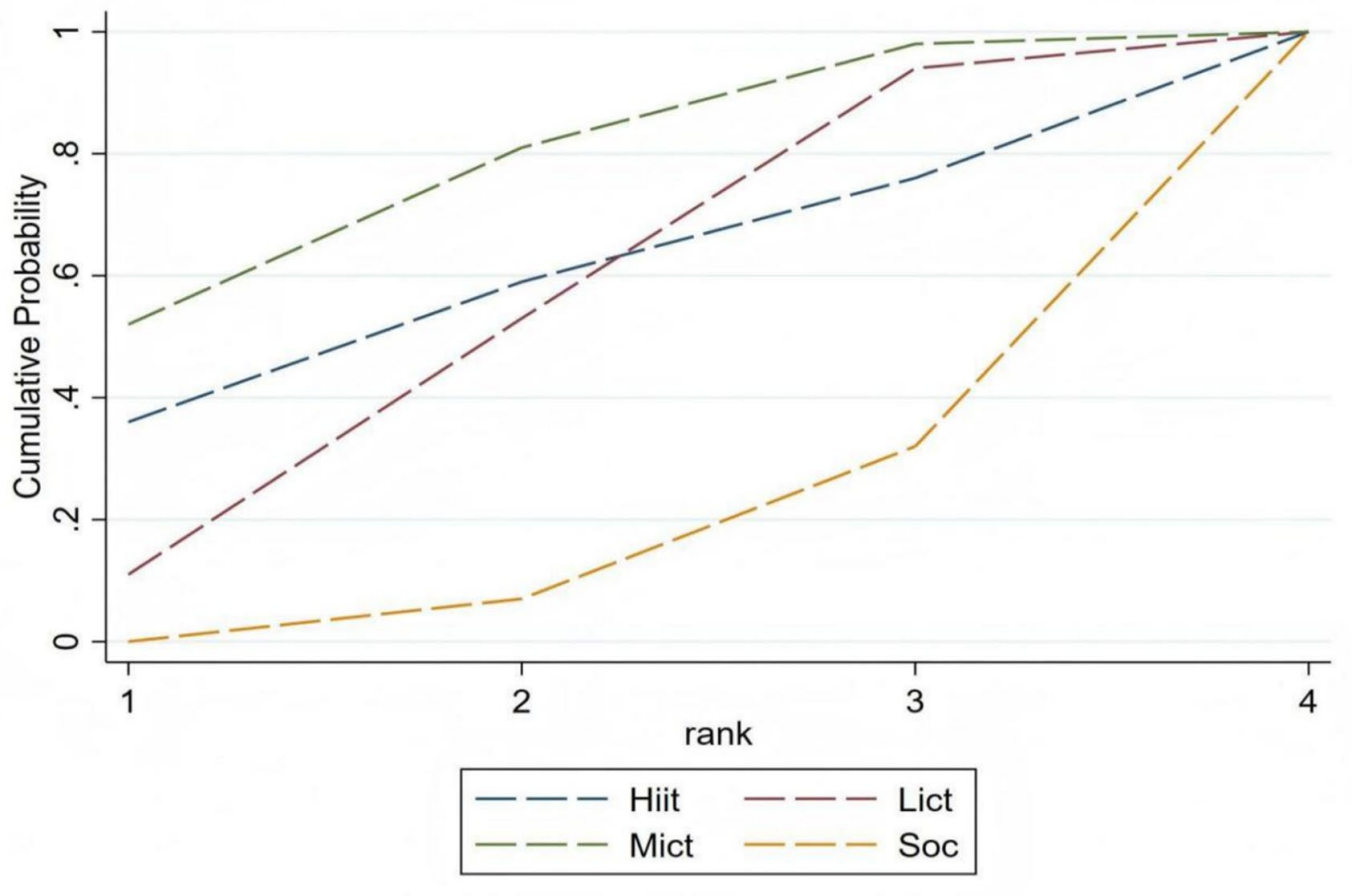
Summary rank plots for TUG. The larger the area under the curve, the more likely the intervention shows the best effect across the outcome measures.

Ranking probabilities derived from the SUCRA are illustrated in Figures 7A–D, 8. Among individuals with chronic stroke, HIIT exhibited the greatest likelihood of being the most effective intervention for enhancing 6MWT performance (SUCRA = 96.2), followed by MICT (SUCRA = 75.2) and HICT (SUCRA = 61.8). Similarly, HIIT ranked highest for improving 10MWT outcomes (SUCRA = 99.8), with MICT occupying the second position (SUCRA = 71.3). For VO₂peak, HIIT (SUCRA = 99.4) again demonstrated the strongest probability of superiority, ahead of MICT (SUCRA = 78.8) and HICT (SUCRA = 54.2). Regarding balance performance assessed by the BBS, HIIT remained the top-ranked intervention (SUCRA = 82.9), followed by MICT (SUCRA = 65.2) and LICT (SUCRA = 49.9). In contrast, for the TUG, MICT (SUCRA = 77.1) emerged as the most effective intervention, surpassing HIIT (SUCRA = 57.2).

### 3.4 Assessment of publication bias, sensitivity, and meta-regression analyses

Potential publication bias was examined for the outcomes, including using funnel plots (see Supplementary Figures 6–10). The distribution of study estimates appeared largely symmetrical, with no conspicuous outliers or wide dispersion, indicating a low likelihood of publication bias in the present network meta-analysis. Across the outcome measures of 6MWT, 10MWT, VO_2_peak, BBS, and TUG the leave-one-out sensitivity analyses revealed no evidence that any single study exerted a decisive influence on the network estimates. In each iteration, one study was sequentially omitted, and a consistency-based random-effects network meta-analysis was re-estimated using the remaining data. Regardless of which studies were excluded, the overall direction of the composite effect of most training interventions compared to SOC remained consistent. Therefore, the study results are generally robust. (see Supplementary Tables 8–12) Additionally, this study conducted a sensitivity analysis by excluding studies with a stroke onset time of 3–6 months and only including studies with a stroke onset time greater than 6 months. The results showed that for outcome measures such as the 10MWT, BBS, and TUG, the statistical significance and direction of the comparisons between interventions did not undergo significant changes, indicating that the results are robust. (see Supplementary Figures 16–18).

On the other hand, for outcome metrics such as VO_2_peak, 6MWT, 10MWT, BBS, and TUG, we conducted a univariate network meta-regression with average age, total intervention duration, baseline gait speed, and baseline VO_2_peak as covariates to test whether these factors may influence the results of this study. The results showed that these individual factors did not have a significant impact on the conclusions of this study. (see Supplementary Tables 13–31).

### 3.5 Assessment of evidence certainty based on the GRADE framework

The certainty of evidence for the primary outcomes, evaluated using the CINeMA framework, spanned from high to very low across all treatment contrasts. For the 6MWT, among 15 pairwise comparisons, 1 comparison (7%) was rated as low certainty, whereas the remaining 14 (93%) were judged to have very low certainty.

For the 10MWT, of the 15 comparisons assessed, 1 (7%) achieved a high rating, 5 (33%) were rated moderate, and 9 (60%) were classified as very low. Regarding VO_2_peak, out of 15 contrasts, 3 (20%) were graded as moderate, 1 (7%) as low, and 11 (73%) as very low certainty.

For the BBS, all 15 evaluations (100%) were assigned very low certainty ratings. Likewise, for the TUG, all 6 comparisons (100%) were graded as very low certainty. A complete summary of the CINeMA evaluations is presented in Supplementary Tables 3–7 and Supplementary Figures 11–15.

## 4 Discussion

### 4.1 Main findings and general interpretation

To the best of our knowledge, this study is the first to systematically compare the effects of different intensities of aerobic exercise on functional capacity and cardiopulmonary function in chronic stroke patients (onset time ≥ 3 months) using network meta-analysis. We included a total of 33 RCTs (*n* = 1,665) to comprehensively assess the effects of different intensities of aerobic exercise on VO_2_peak, walking and mobility abilities (6MWT, 10MWT, TUG), and balance function (BBS). The overall results indicate that most aerobic exercise programs, compared to SOC, lead to positive physiological and clinical changes related to stroke recovery, as evidenced by improvements in VO_2_peak and mobility abilities. Importantly, this study suggests that exercise intensity may be a key modulating factor for intervention effect differences: HIIT and MICT consistently show greater benefits across multiple outcomes, whereas the effects of HICT appear relatively unstable. This finding implies that, in the context of stroke-related neuro-muscular impairment, decreased cardiopulmonary reserve, and fatigue susceptibility, ‘higher intensity’ does not necessarily translate to better outcomes. The feasibility, tolerance, and load-recovery structure of the intervention may also be critical. Compared to sustained high-intensity loads, HIIT alternates between short bouts of high-intensity exercise and recovery periods, and provides sufficient training stimulus at more manageable levels of fatigue. This approach may be more conducive to maintaining training dosage and adherence, ultimately resulting in more stable functional improvements.

From a physiological mechanism perspective, moderate to high-intensity aerobic training may promote recovery through multiple pathways. Firstly, by enhancing skeletal muscle oxidative metabolism capacity (such as improving mitochondrial biogenesis and efficiency), thereby boosting energy supply; secondly, by increasing capillary density and improving muscle perfusion, which facilitates the delivery and utilization of oxygen and nutrients; and thirdly, potentially by influencing processes related to neuroplasticity, improving neuromuscular system efficiency and enhancing motor control and coordination (5, 56, 57). The synergistic effects of these adaptations may, to some extent, explain the improvements in outcomes such as VO_2_peak, walking, and mobility abilities. Based on these findings of this study, clinicians and community exercise instructors may consider HIIT or MICT as preferred options for aerobic exercise prescriptions for chronic stroke, under the premise of risk assessment and monitoring. These interventions should be tailored to the patient’s functional level, comorbid conditions, fatigue responses, and safety thresholds, with individualized progression to enhance both feasibility and clinical benefits.

The results of this study indicate that aerobic exercise at different intensities can improve VO_2_peak in chronic stroke patients, with HIIT, MICT, and HICT potentially being more effective with the effect sizes range from approximately 1.82 to 5.50 mL·kg^−1^·min^−1^. This result is generally consistent with previous evidence on the enhancement of cardiopulmonary fitness through aerobic training after stroke (23, 58). The potential mechanism may be related to the improvement of hemodynamic efficiency and vascular health through moderate to high-intensity training: exercise can increase stroke volume and cardiac output, enhance oxygen delivery and peripheral uptake efficiency, and improve resting heart rate and blood pressure regulation, thereby reducing the cardiovascular system’s workload and enhancing exercise tolerance (59).

Improvement in VO_2_peak holds significant clinical importance. Previous studies have indicated that for each 1 mL·kg^−1^·min^−1^ increase in VO_2_peak, cardiovascular morbidity can be reduced by approximately 10% (60), furthermore, an increase of 3 mL·kg^−1^·min^−1^ can reduce hospitalization rates and the risk of ischemic stroke by 7 and 9%, respectively, in stroke patients (61, 62). As a result, using VO_2_peak as a primary outcome measure has strong clinical interpretability.

In terms of intensity comparison, consistent with previous findings (63), this study also suggests that HIIT may be a superior approach for improving VO_2_peak in stroke patients and shows a statistical advantage over MICT. Although the physiological mechanisms by which HIIT improves VO_2_peak in stroke populations are not yet fully understood, studies in non-stroke populations have shown that HIIT more strongly activates signaling pathways related to mitochondrial biogenesis and further enhances skeletal muscle mitochondrial density and respiratory capacity through repeated high-intensity stimuli (64–67). Stroke-related studies (68, 69) also suggest that HIIT may more readily induce central cardiovascular adaptations (such as improvements in cardiac output-related indicators) compared to MICT, and trigger acute changes in neurotrophic biomarkers. Regardless, the above evidence is still in the developmental stage, and further high-quality RCTs are needed to clarify the central and peripheral adaptation pathways driven by different intensity prescriptions. This is particularly important in understanding the relationship between training intensity and mitochondrial remodeling, endothelial function, and autonomic nervous regulation.

Moreover, ventilatory thresholds, which reflect a physiological turning point in exercise intensity, may be more beneficial for individualized intensity prescription compared to simply measuring VO_2_peak (70). Due to the limited number of eligible studies at present (see Supplementary Table 32), this study did not systematically evaluate this. Future study designs are recommended to include more refined physiological indicators, such as ventilatory thresholds, to enhance prescription accuracy and improve the mechanistic understanding of intensity stratification.

The results of this study are consistent with previous findings (7, 12, 71), indicating that aerobic exercise at different intensities can improve walking ability (6MWT, 10MWT) to varying extents in chronic stroke patients. Restoring gait ability is crucial for stroke patients, as prolonged inactivity leads to physical deconditioning, which not only impairs normal activity levels but also delays overall recovery (72). Since aerobic exercise often involves dynamic repetitive training forms such as treadmill walking or cycling, it can enhance lower limb endurance, gait efficiency, and neuromuscular coordination, thereby promoting improvements in functional walking ability (73), which is reflected in better walking-related outcomes.

Relevant studies indicate that the 6MWT reflects an individual’s walking endurance, influenced by cardiovascular health and neuromotor function, making it one of the key outcome measures for assessing gait function post-stroke (74). Compared to SOC, both HIIT (MD = 63.04, 95% CI [14.38, 111.69]) and MICT (MD = 39.23, 95% CI [4.13, 74.34]) significantly improved the 6MWT distance in chronic stroke patients, achieving the Minimal Clinically Important Difference (MCID) for the 6MWT in stroke patients (approximately 34–44 meters) (75), even so no significant statistical difference was found between HIIT and MICT. The outcome suggests that both HIIT and MICT may be effective approaches for improving endurance-based walking ability. Additionally, LICT showed even less improvement in the 6MWT distance compared to SOC, likely due to its lower intensity. Hence, in clinical practice, the choice of intervention can be based on safety, feasibility, and patient preference.

Gait speed is widely regarded as another important indicator of functional recovery in stroke patients, with improvements often being associated with the ability to independently perform activities of daily living (76). 10MWT, as a standard tool for measuring gait speed, not only reflects the extent of gait improvement but also demonstrates strong clinical predictive value in forecasting independent community walking and daily functional abilities (77). The repercussion of this study indicate that all aerobic exercise programs can improve walking speed, with HIIT showing a significantly greater improvement compared to other programs. This may be due to HIIT’s more effective activation of fast-twitch muscle fibers and enhanced motor unit recruitment (78). MICT also has a good effect on improving the 10MWT speed in patients, while the improvement with HICT and LICT is relatively limited. This may be due to factors such as the patients’ inability to sustain high-intensity exercise for a long period or the lower intensity levels. It is important to note that the 10MWT is sensitive to baseline walking speed, measurement protocols, use of assistive devices, and whether other rehabilitation interventions are combined. Future studies should focus on standardizing the prescription and reporting of assessment protocols to reduce heterogeneity and enhance comparability.

Regarding the TUG, MICT, HIIT, and LICT all lead to some improvement in test performance, but there are no statistically significant differences between the vast majority of interventions. This result may suggest that the mobility ability reflected by the TUG is influenced by multiple factors beyond cardiopulmonary fitness, including sit-to-stand ability, turning control, dynamic balance, and lower limb strength (74). Therefore, a single aerobic stimulus may not produce clear intensity gradient differences.

Regarding the improvement in balance ability with each aerobic exercise program, although all aerobic exercise programs showed a significant increase in BBS scores compared to SOC (approximately 0.95–5.37 points), the overall improvement is likely to fall below the MCID of the BBS (approximately 6 points) (79). This suggests that the direct transfer effects of aerobic exercise on balance may be limited. Considering that balance impairments post-stroke are closely related to muscle strength, joint range of motion, postural control strategies, and sensory integration (80), a relatively singular aerobic training modality may be insufficient to address the key factors influencing balance. Therefore, future studies should focus more on multimodal combined interventions (such as aerobic and resistance, balance control training or task-oriented gait training) for comprehensive improvement in balance ability and fall risk.

### 4.2 Safety of intensity selection and recommendations for individualized prescriptions

In spite of the fact that this study generally support that HIIT may offer better average effects, it is crucial to emphasize safety boundaries when implementing higher-intensity training in stroke populations. Physiological studies suggest that when exercise intensity reaches higher levels, cerebrovascular changes may occur in response to maintain homeostasis (81), and stroke patients may experience impaired cerebrovascular reactivity and autonomic regulation, which can increase the potential risks associated with sustained high-intensity loads. Compared to HICT, HIIT includes recovery periods and allows more flexible combinations of intensity and duration, may enhance tolerance and feasibility while still providing sufficient training stimulus (71, 82). Based on real-world clinical scenarios, MICT also demonstrates good outcomes and is easier to implement. Therefore, aerobic exercise prescriptions should follow an individualized approach, taking into account the patient’s comorbidities, functional level, symptom responses during and after training, and monitoring indicators. A gradual progression strategy should be used, and when necessary, a combination of different intensity protocols should be employed to optimize the risk–benefit ratio.

### 4.3 Study strengths, limitations, and the significance of the chronic phase restriction

Another strength of this study is the inclusion of chronic stroke patients (≥3 months), which helps reduce potential confounding factors related to clinical management, fluctuations in complications, and natural recovery during the acute or subacute phases, thereby allowing for a more reliable observation of the relative effects of exercise interventions. Albeit previous studies suggest that patients with a shorter onset time may respond more significantly to exercise interventions, this may be related to the spontaneous neural connection repair window during the acute phase (83, 84), patients in the chronic phase can still promote functional recovery through intensive training, especially in terms of neural remodeling and functional adaptation (85). Since the pathological state in the chronic phase is more stable (86), the study results are more favorable for assessing the sustainability and real-world effects of the intervention.

At the same time, this study has limitations that should be interpreted with caution: The certainty of evidence was predominantly low or very low across most treatment comparisons, particularly for the BBS and TUG outcomes. The most common driver of downgrading was within-study bias, reflecting the inherent difficulty of blinding participants and intervention providers in exercise-based RCTs. In addition, the limited number of studies for BBS and TUG may have further contributed to the imprecision of these comparisons, as smaller networks tend to produce wider confidence intervals. Future studies should consider innovative blinding strategies to minimize expectancy effects. Larger, multi-center RCTs with adequate allocation concealment and prospectively registered protocols are also needed to improve the precision of estimates and elevate the overall quality of the evidence base.

Second, marked heterogeneity in exercise protocols (intensity parameters, modalities, frequency, and duration) may compromise the transitivity assumed in the NMA. To reduce ambiguity from overlapping intensity ranges, we adopted a conservative classification strategy based on the reported minimum intensity threshold. While this approach avoids overestimating effects, it may have diluted the true differences between intensity categories, especially when the actual training stimulus was near the upper boundary of an assigned zone. Future studies should adopt standardized reporting frameworks such as the FITT principle or the AEROBICS 2019 guidelines and, when possible, employ pure aerobic exercise arms alongside head-to-head comparisons of aerobic exercise alone versus combined interventions to improve comparability.

Third, individual differences across study populations, including baseline functional status and stroke type, may have contributed to the heterogeneity of treatment effects. Although we conducted meta-regression with baseline gait speed and VO_2_peak as covariates, the influence of other unmeasured or inconsistently reported baseline characteristics on the robustness of our findings cannot be ruled out. Additionally, the majority of participants in the included trials had preserved ambulatory capacity, which restricts the extrapolation of our results to non-ambulatory patients or those with more severe motor deficits. Future studies should prospectively collect and report outcomes by key clinical subgroups and extend recruitment to populations with a broader range of functional severities.

## 5 Conclusion

To sum up, aerobic exercise can improve cardiopulmonary fitness, mobility, and balance ability in chronic stroke patients. Considering the stability of the overall effects and clinical feasibility, HIIT and MICT may be more advantageous prescription options, while the unstable effects of HICT suggest that solely pursuing sustained high intensity may not be optimal. Exercise prescriptions should emphasize individualization and safety monitoring, and may consider combining with resistance and balance-specific training to achieve more comprehensive functional benefits.

## Data Availability

The datasets presented in this study can be found in online repositories. The names of the repository/repositories and accession number(s) can be found in the article/Supplementary material.
