# Supplementary material for: Comparative effectiveness of aerobic training intensities in chronic stroke: a network meta-analysis of randomized controlled trials
Source: Front Neurol. 2026 Jul 6;17:1808908. doi: 10.3389/fneur.2026.1808908 (PMC13381741; doi:10.3389/fneur.2026.1808908)
Supplement: Supplementary file 1 [file Table_1.docx]

| **Table of Contents** | | |
| --- | --- | --- |
| Title | Content | page |
| Table S1 | PRISMA NMA Checklist of Items to Include When Reporting a Systematic Review Involving a Network Meta-analysis | 1-4 |
| Table S2 | Literature Search Strategy | 5-7 |
| Table S3 | CINeMA-based evaluation of evidence certainty for the VO_2peak_ outcome network. | 8 |
| Table S4 | CINeMA-based evaluation of evidence certainty for the Six-Minute Walk Test (6MWT) outcome network. | 9 |
| Table S5 | CINeMA-based evaluation of evidence certainty for the 10-Meter Walk Test (10MWT) outcome network. | 10 |
| Table S6 | CINeMA-based evaluation of evidence certainty for the Berg Balance Scale (BBS) outcome network. | 11 |
| Table S7 | CINeMA-based evaluation of evidence certainty for the Time Up and Go (TUG) outcome network. | 12 |
| Table S8 | Sensitivity Analysis of the VO_2peak_ outcome | 13-15 |
| Table S9 | Sensitivity Analysis of the 6MWT outcome | 16-18 |
| Table S10 | Sensitivity Analysis of the 10MWT outcome | 19-20 |
| Table S11 | Sensitivity Analysis of the BBS outcome | 21-22 |
| Table S12 | Sensitivity Analysis of the TUG outcome | 23 |
| Table S13 | Meta-Regression Analysis of the VO_2peak_ Using Age as a Covariate | 24 |
| Table S14 | Meta-Regression Analysis of the VO_2peak_ Using Total intervention duration(min) as a Covariate | 25 |
| Table S15 | Meta-Regression Analysis of the VO_2peak_ Using Baseline gait speed as a Covariate | 26 |
| Table S16 | Meta-Regression Analysis of the VO_2peak_ Using Baseline VO_2peak_ as a Covariate | 27 |
| Table S17 | Meta-Regression Analysis of the 6MWT Using Age as a Covariate | 28 |
| Table S18 | Meta-Regression Analysis of the 6MWT Using Total intervention duration as a Covariate | 29 |
| Table S19 | Meta-Regression Analysis of the 6MWT Using Baseline gait speed as a Covariate | 30 |
| Table S20 | Meta-Regression Analysis of the 6MWT Using Baseline VO_2peak_ as a Covariate | 31 |
| Table S21 | Meta-Regression Analysis of the 10MWT Using Age as a Covariate | 32 |
| Table S22 | Meta-Regression Analysis of the 10MWT Using Total intervention duration as a Covariate | 33 |
| Table S23 | Meta-Regression Analysis of the 10MWT Using Baseline gait speed as a Covariate | 34 |
| Table S24 | Meta-Regression Analysis of the 10MWT Using Baseline VO_2peak_ as a Covariate | 35 |
| Table S25 | Meta-Regression Analysis of the BBS Using Age as a Covariate | 36 |
| Table S26 | Meta-Regression Analysis of the BBS Using Total intervention duration as a Covariate | 37 |
| Table S27 | Meta-Regression Analysis of the BBS Using Baseline gait speed as a Covariate | 38 |
| Table S28 | Meta-Regression Analysis of the BBS Using Baseline VO_2peak_ as a Covariate | 39 |
| Table S29 | Meta-Regression Analysis of the TUG Using Age as a Covariate | 40 |
| Table S30 | Meta-Regression Analysis of the TUG Using Total intervention duration as a Covariate | 41 |
| Table S31 | Meta-Regression Analysis of the TUG Using Baseline gait speed as a Covariate | 42 |
| Table S32 | Baseline Ventilatory Threshold, Fugl-Meyerleg motor score, and National Institutes of Health Stroke Scale (NIHSS) | 43-45 |
| Figure S1 | Risk of bias assessment of included RCTs | 46 |
| Figure S2 | Loop-specific inconsistency test for the VO_2peak_ | 47 |
| Figure S3 | Loop-specific inconsistency test for the 6MWT outcome | 48 |
| Figure S4 | Loop-specific inconsistency test for the 10MWT outcome | 49 |
| Figure S5 | Loop-specific inconsistency test for the BBS outcome | 50 |
| Figure S6 | Comparison-adjusted funnel plot for the VO_2peak_ outcome | 51 |
| Figure S7 | Comparison-adjusted funnel plot for the 6MWT outcome | 52 |
| Figure S8 | Comparison-adjusted funnel plot for the 10MWT outcome | 53 |
| Figure S9 | Comparison-adjusted funnel plot for the BBS outcome | 54 |
| Figure S10 | Comparison-adjusted funnel plot for the TUG outcome | 55 |
| Figure S11 | CINeMA contribution plot of within-study bias (RoB 2.0) for theVO_2peak_ outcome | 56 |
| Figure S12 | CINeMA contribution plot of within-study bias (RoB 2.0) for the 6MWT outcome | 57 |
| Figure S13 | CINeMA contribution plot of within-study bias (RoB 2.0) for the 10MWT outcome | 58 |
| Figure S14 | CINeMA contribution plot of within-study bias (RoB 2.0) for the BBS outcome | 59 |
| Figure S15 | CINeMA contribution plot of within-study bias (RoB 2.0) for the TUG outcome | 60 |
| Figure S16 | League tables of treatment effects from the network meta-analysis across aerobic training intensities in chronic stroke(≥6 months): 10MWT | 61 |
| Figure S17 | League tables of treatment effects from the network meta-analysis across aerobic training intensities in chronic stroke(≥6 months): BBS | 62 |
| Figure S18 | League tables of treatment effects from the network meta-analysis across aerobic training intensities in chronic stroke(≥6 months): TUG | 63 |

Evaluating the comparative effectiveness of aerobic training intensities on cardiopulmonary fitness and functional outcomes in chronic stroke: A systematic review and network meta-analysis of randomized controlled trials.

Supplementary Table 1 PRISMA NMA Checklist of Items to Include When Reporting a Systematic Review Involving a Network Meta-analysis

| **Section/Topic** | **Item #** | **Checklist Item** |
| --- | --- | --- |
| **TITLE** |  |  |
| Title | 1 | Identify the report as a systematic review *incorporating*  *anetwork meta-analysis (or related form of meta-analysis).* |
|  |  |  |
| **ABSTRACT** |  |  |
| Structured summary | 2 | Provide a structured summary including, as applicable:  **Background:** main objectives  **Methods:** data sources; study eligibility criteria, participants, and interventions; study appraisal; and *synthesis methods, such as network meta-analysis.*  **Results:** number of studies and participants identified; summary estimates with corresponding confidence/credible intervals; *treatment rankings may also be discussed. Authors may choose to summarize pairwise comparisons against a chosen treatment included in their analyses for brevity.*  **Conclusions:** limitations; conclusions and implications of findings.  **Other:** systematic review registration number with registry name. |
|  |  |  |
| **INTRODUCTION** |  |  |
| Rationale | 3 | Describe the rationale for the review in the context of what is already known*, including mention of why a network meta-analysis has been conducted.* |
| Objectives | 4 | Provide an explicit statement of questions being addressed, with reference to participants, interventions, comparisons, outcomes, and study design (PICOS). |
|  |  |  |
| **METHODS** |  |  |
| Protocol and registration | 5 | Indicate whether a review protocol exists and if and where it can be accessed (e.g., Web address); and, if available, provide registration information, including registration number. |
| Eligibility criteria | 6 | Specify study characteristics (e.g., PICOS, length of follow-up) and report characteristics (e.g., years considered, language, publication status) used as criteria for eligibility, giving rationale. *Clearly describe eligible treatments included in the treatment network, and note whether any have been clustered or merged into the same node (with justification).* |
| Information sources | 7 | Describe all information sources (e.g., databases with dates of coverage, contact with study authors to identify additional studies) in the search and date last searched. |
| Study selection | 9 | State the process for selecting studies (i.e., screening, eligibility, included in systematic review, and, if applicable, included in the meta-analysis). |

| Data collection process | 10 | Describe method of data extraction from reports (e.g., piloted forms, independently, in duplicate) and any processes for obtaining and confirming data from investigators. |
| --- | --- | --- |
| Geometry of the network | 11 | Describe methods used to explore the geometry of the treatment network under study and potential biases related to it. This should include how the evidence base has been graphically summarized for presentation, and what characteristics were compiled and used to describe the evidence base to readers. |
| Risk of bias within individual studies | 12 | Describe methods used for assessing risk of bias of individual studies (including specification of whether this was done at the study or outcome level), and how this information is to be used in any data synthesis. |
| Summary measures | 13 | State the principal summary measures (e.g., risk ratio, difference in means). *Also describe the use of additional summary measures assessed, such as treatment rankings and surface under the cumulative ranking curve (SUCRA) values, as well as modified approaches used to present summary findings from meta-analyses.* |
| Assessment of Inconsistency | 14 | Describe the statistical methods used to evaluate the agreement of direct and indirect evidence in the treatment network(s) studied. Describe efforts taken to address its presence when found. |
| Risk of bias across studies | 15 | Specify any assessment of risk of bias that may affect the cumulative evidence (e.g., publication bias, selective reporting within studies). |
| Additional analyses | 16 | Describe methods of additional analyses if done, indicating which were pre-specified. This may include, but not be limited to, the following:   - Sensitivity or subgroup analyses; - Meta-regression analyses; - *Alternative formulations of the treatment network; and* - *Use of alternative prior distributions for Bayesian analyses (if applicable).* |
| **RESULTS†** |  |  |
| Study selection | 17 | Give numbers of studies screened, assessed for eligibility, and included in the review, with reasons for exclusions at each stage, ideally with a flow diagram. |
| Presentation of network structure | 18 | Provide a network graph of the included studies to enable visualization of the geometry of the treatment network. |
| Summary of network geometry | 19 | Provide a brief overview of characteristics of the treatment network. This may include commentary on the abundance of trials and randomized patients for the different interventions and pairwise comparisons in the network, gaps of evidence in the treatment network, and potential biases reflected by the network structure. |
| Study characteristics | 20 | For each study, present characteristics for which data were extracted (e.g., study size, PICOS, follow-up period) and provide the citations. |
| Risk of bias within studies | 21 | Present data on risk of bias of each study and, if available, any outcome level assessment. |
| Results of individual studies | 22 | For all outcomes considered (benefits or harms), present, for each study: 1) simple summary data for each intervention group, and 2) effect estimates and confidence intervals. *Modified approaches may be needed to deal with information from larger networks.* |

| Synthesis of results | 23 | Present results of each meta-analysis done, including confidence/credible intervals. *In larger networks, authors may focus on comparisons versus a particular comparator (e.g. placebo or standard care), with full findings presented in an appendix. League tables and forest plots may be considered to summarize pairwise comparisons.* If additional summary measures were explored (such as treatment rankings), these should also be presented. |
| --- | --- | --- |
| Exploration for inconsistency | 24 | Describe results from investigations of inconsistency. This may include such information as measures of model fit to compare consistency and inconsistency models, *P* values from statistical tests, or summary of inconsistency estimates from different parts of the treatment network. |
| Results of additional analyses | 25 | Give results of additional analyses, if done (e.g., sensitivity or subgroup analyses, meta-regression analyses*, alternative network geometries studied, alternative choice of prior distributions for Bayesian analyses,* and so forth). |
|  |  |  |
| **DISCUSSION** |  |  |
| Summary of evidence | 26 | Summarize the main findings, including the strength of evidence for each main outcome; consider their relevance to key groups (e.g., healthcare providers, users, and policy-makers). |
| Limitiation | 27 | Discuss limitations at study and outcome level (e.g., risk of bias), and at review level (e.g., incomplete retrieval of identified research, reporting bias). *Comment on the validity of the assumptions, such as transitivity and consistency. Comment on any concerns regarding network geometry (e.g., avoidance of certain comparisons).* |
| Conclusions | 28 | Provide a general interpretation of the results in the context of other evidence, and implications for future research. |
|  |  |  |
| **FUNDING** |  |  |
| Funding | 29 | Describe sources of funding for the systematic review and other support (e.g., supply of data); role of funders for the systematic review. This should also include information regarding whether funding has been received from manufacturers of treatments in the network and/or whether some of the authors are content experts with professional confLICTs of interest that could affect use of treatments in the network. |
|  |  |  |

PICOS = population, intervention, comparators, outcomes, study design.

* Text in italics indicate S wording specific to reporting of network meta-analyses that has been added to guidance from the PRISMA statement.

† Authors may wish to plan for use of appendices to present all relevant information in full detail for items in this section.

| **Supplementary Table 2.Literature Search Strategy** | |
| --- | --- |
| **Pubmed** | #1 stroke[MeSH Terms] 197554  #2 ((((((((((((((((((stroke[MeSH Terms]) ) OR (Cerebrovascular Accident[Title/Abstract])) OR (Cerebrovascular Accidents[Title/Abstract])) OR (Cerebral Stroke[Title/Abstract])) OR (Cerebral Strokes[Title/Abstract])) OR (Stroke, Cerebral[Title/Abstract])) OR (Cerebrovascular Apoplexy[Title/Abstract])) OR (Apoplexy, Cerebrovascular[Title/Abstract])) OR (Vascular Accident, Brain[Title/Abstract])) OR (Brain Vascular Accident[Title/Abstract])) OR (Vascular Accidents, Brain[Title/Abstract])) OR (Stroke, Cerebrovascular[Title/Abstract])) OR (Strokes,Cerebrovascular[Title/Abstract])) OR (Stroke, Acute[Title/Abstract])) OR (Acute Strokes[Title/Abstract])) OR (Cerebrovascular Accident, Acute[Title/Abstract])) OR (Acute Cerebrovascular Accident[Title/Abstract])) OR (Cerebrovascular Accidents, Acute[Title/Abstract]) 206885  #3 Exercise[MeSH Terms] 282131  #4 (((((((((((((((((Exercise[MeSH Terms])) OR (Exercise, Physical[Title/Abstract])) OR (Physical, Exercise[Title/Abstract])) OR (Physical, Exercises[Title/Abstract])) OR (Exercise, Aerobic[Title/Abstract])) OR (Aerobic Exercises[Title/Abstract])) OR (Exercises, Aerobic[Title/Abstract])) OR (Acute Exercise[Title/Abstract])) OR (Acute Exercises[Title/Abstract])) OR (Exercise, Acute[Title/Abstract])) OR (Exercise Training[Title/Abstract])) OR (Exercise Trainings[Title/Abstract])) OR (Trainings, Exercise[Title/Abstract])) OR (Physical Activity[Title/Abstract])) OR (Activities, Physical[Title/Abstract])) OR (Activity, Physical[Title/Abstract])) OR (Physical Activities[Title/Abstract])) 465828  #5 ((Randomized Controlled Trial[Publication Type]) OR (randomized[Title/Abstract])) OR (placebo[Title/Abstract]) 1172386  #6 #2 AND #4 AND #5 1243 |
| **Cochrane** | #1 MeSH descriptor: [Stroke] explode all trees 18393  #2 ("Cerebrovascular Accident" OR "Cerebrovascular Accidents" OR "Cerebral Stroke" OR "Cerebral Strokes" OR "Stroke, Cerebral" OR "Stroke, Cerebral" OR "Cerebrovascular Apoplexy" OR "Vascular Accident, Brain" OR "Brain Vascular Accident" OR "Brain Vascular Accidents" OR "Cerebrovascular Stroke" OR "Cerebrovascular Strokes" OR "Strokes, Cerebrovascular" OR "CVAs (Cerebrovascular Accident)" OR "Stroke, Acute" OR "Acute Stroke" OR "Cerebrovascular Accident, Acute" OR "Chronic Stroke" OR "Chronic Strokes" OR "Strokes,Chronic" OR "Stroke,Chronic"):ti,ab,kw 26522  #3 #1 OR #2 38673  #4 MeSH descriptor: [Exercise] explode all trees 42079  #5 ("Aerobic Exercise" OR "Aerobic Exercises" OR "Exercises, Aerobic" OR "Acute Exercises" OR "Stroke, Cerebral" OR "Acute Exercise" OR "Exercise Training" OR "Exercises, Acute" OR "Brain Vascular Accident" OR "Brain Vascular Accidents" OR "Exercise Trainings" OR "Trainings, Exercise" OR "Activities, Physical" OR "Physical Activities"):ti,ab,kw 25526  #6 #4 OR #5 60601  #7 ("randomized controlled trial" OR "controlled trial" OR "controlled study" OR "controlled clinical trial" OR "RCT" OR "randomly" OR "randomized"):ti,ab,kw 1548985  #8 #3 AND #6 AND #7 1133 |
| **EMBASE** | #1 'cerebrovascular accident'/exp' 523217  #2 'cerebrovascular accidents':ab,ti OR 'cerebral stroke':ab,ti OR 'cerebral strokes':ab,ti OR  'cerebral stroke':ab,ti OR 'strokes, cerebral':ab,ti OR 'brain vascular accident':ab,ti OR 'brain vascular accidents':ab,ti OR 'cerebrovascular stroke':ab,ti OR 'cerebrovascular strokes':ab,ti OR 'stroke, cerebrovascular':ab,ti OR 'apoplexy':ab,ti OR 'stroke, acute':ab,ti OR 'acute stroke':ab,ti OR 'acute strokes':ab,ti OR 'cerebrovascular accident, acute':ab,ti OR 'chronic stroke':ab,ti OR 'chronic strokes':ab,ti OR 'strokes,chronic':ab,ti OR 'stroke,chronic':ab,ti OR 'acute cerebrovascular accidents':ab,ti 15027  #3 #1 OR #2 538289  #4 'exercise'/exp 548727  #5 'exercise, physical':ab,ti OR 'exercises, physical':ab,ti OR 'physical exercise':ab,ti OR 'physical exercises':ab,ti OR 'exercise, aerobic':ab,ti OR 'aerobic exercise':ab,ti OR 'aerobic exercises':ab,ti OR 'exercises, aerobic':ab,ti OR 'acute exercise':ab,ti OR 'acute exercises':ab,ti OR 'exercise, acute':ab,ti OR 'exercises, acute':ab,ti OR 'exercise training':ab,ti OR 'training, exercise':ab,ti OR 'trainings, exercise':ab,ti OR 'physical activity':ab,ti OR 'activities, physical':ab,ti OR 'activity, physical':ab,ti OR 'physical activities':ab,ti 184729  #6 #4 OR #5 733456  #7 'randomized controlled trial'/exp OR 'placebo':ti,ab 1341169  #8 #3 AND #6 AND #7 2884 |
| **Scopus** | #1 TITLE-ABS-KEY ("cerebrovascular accident" OR "cerebrovascular accidents" OR "cerebral stroke" OR "cerebral strokes" OR "strokes, cerebral" OR "cerebrovascular apoplexy" OR"apoplexy, cerebrovascular" OR "vascular accident, brain" OR "brain vascular accident" OR "brain vascular accidents" OR "vascular accidents, brain"OR "cerebrovascular stroke" OR "cerebrovascular strokes" OR "stroke, cerebrovascular" OR "strokes, cerebrovascular" OR "cva" OR "cvas" OR "stroke, acute" OR "acute stroke" OR "acute strokes" OR "strokes, acute" OR "cerebrovascular accident, acute" OR "acute cerebrovascular accident"OR "acute cerebrovascular accidents" OR "cerebrovascular accidents, acute" OR "stroke" OR "chronic stroke" OR "chronic strokes" OR " strokes,chronic" OR " stroke,chronic") 706731  #2 TITLE-ABS-KEY ("exercise, physical" OR "exercises, physical" OR "physical exercise" OR "physical exercises" OR "exercise, aerobic" OR "aerobic exercise" OR "aerobic exercises" OR "exercises, aerobic" OR "exercise, isometric" OR "exercises, isometric" OR "isometric exercises" OR "isometric exercise" OR "acute exercise" OR "acute exercises" OR "exercise, acute" OR "exercises, acute" OR "exercise trainings" OR "training, exercise" OR "trainings, exercise" OR"physical activity" OR "activities, physical" OR "activity, physical"OR "physical activities" OR "exercise") 1,136,331  #3 TITLE-ABS-KEY ("Randomized" OR "placebo" OR "randomized controlled trial") 1800140  #4 #1 AND #2 AND #3 3230 |
| **Web of science** | #1 Stroke (Topic) OR Cerebrovascular Accident (Topic) OR Cerebrovascular Accidents (Topic) OR Cerebral Stroke (Topic) OR Cerebral Strokes (Topic) OR Stroke, Cerebral (Topic) OR Cerebrovascular Apoplexy (Topic) OR Apoplexy, Cerebrovascular (Topic) OR Vascular Accident, Brain (Topic) OR Brain Vascular Accident (Topic) OR Vascular Accidents, Brain (Topic) OR Stroke, Cerebrovascular (Topic) OR Strokes,Cerebrovascular (Topic) OR Stroke, Acute (Topic) OR Stroke, Chronic (Topic) OR StrokeS, Chronic (Topic) OR Chronic Stroke (Topic) OR Chronic Strokes (Topic) 491837  #2 Exercise (Topic) OR Exercise, Physical (Topic) OR Physical, Exercise (Topic) OR Exercise, Aerobic (Topic) OR Aerobic Exercises (Topic) OR Exercises, Aerobic (Topic) OR Acute Exercise (Topic) OR Acute Exercises (Topic) OR Exercise Training (Topic) OR Exercise Trainings (Topic) OR Trainings, Exercise (Topic) OR Physical Activity (Topic) OR Activities, Physical (Topic) OR Activity, Physical (Topic) OR Physical Activities (Topic) 9226954  #3 Randomized Controlled Trial (Publication Titles) OR randomized (Topic) OR placebo (Topic) 1272101  #4 #1 AND #2 AND #3 3813 |

| **EBSCO_host_** | #1 SU (Stroke) OR TI ((cerebrovascular accident or Strokes,Cerebrovascular) OR (Cerebrovascular Accidents or Stroke, Acute) OR (cerebral stroke or Cerebrovascular Accident, Acute) OR (Stroke, Cerebral or Acute Cerebrovascular Accident) OR (Cerebrovascular Apoplexy or Cerebrovascular Accidents, Acute) OR (Apoplexy, Cerebrovascular) OR (Vascular Accident, Brain) OR (Brain Vascular Accident) OR (Vascular Accidents, Brain) OR (Stroke, Cerebrovascular)) OR AB ((cerebrovascular accident or Strokes,Cerebrovascular) OR (Cerebrovascular Accidents or Stroke, Acute) OR (cerebral stroke or Cerebrovascular Accident, Acute) OR (Stroke, Cerebral or Acute Cerebrovascular Accident) OR (Cerebrovascular Apoplexy or Cerebrovascular Accidents, Acute) OR (Apoplexy, Cerebrovascular) OR (Vascular Accident, Brain) OR (Brain Vascular Accident) OR (Vascular Accidents, Brain) OR (Stroke, Cerebrovascular)) 383011  #2 SU (exercise) OR TI ((Exercise, Physical) or (Physical, Exercise) or (Physical, Exercises) or (Exercise, Aerobic) or (Aerobic Exercises) or (Exercises, Aerobic) or (Acute Exercise) or (Acute Exercises) or (Exercise, Acute) or (Exercise Training) or (Exercise Trainings) or (Trainings, Exercise) or (Physical Activity) or (Activities, Physical) or (Physical Activities)) OR AB ((Exercise, Physical) or (Physical, Exercise) or (Physical, Exercises) or (Exercise, Aerobic) or (Aerobic Exercises) or (Exercises, Aerobic) or (Acute Exercise) or (Acute Exercises) or (Exercise, Acute) or (Exercise Training) or (Exercise Trainings) or (Trainings, Exercise) or (Physical Activity) or (Activities, Physical) or (Physical Activities)) 789366  #3 TI (Randomized Controlled Trial) OR AB (Randomized Controlled Trial) OR TI (randomized) OR AB (randomized) OR TI (placebo) OR AB (placebo) 1558176  #4 #1 AND #2 AND #3 3118 |
| --- | --- |

Supplementary Table 3.CINeMA-based evaluation of evidence certainty for the VO_2peak_ outcome network.

| **Comparison** | **Number of studies** | **Within-study bias** | **Reporting bias** | **Indirectness** | **Imprecision** | **Heterogeneity** | **Incoherence** | **Confidence rating** | **Reason(s) for downgrading** |
| --- | --- | --- | --- | --- | --- | --- | --- | --- | --- |
| HICT:LICT | 1 | Some concerns | Low risk | No concerns | Major concerns | No concerns | No concerns | Very low | ["Within-study bias","Imprecision"] |
| HICT:SOC | 5 | Some concerns | Low risk | No concerns | No concerns | Major concerns | No concerns | Very low | ["Within-study bias","Heterogeneity"] |
| HICT:Non-training | 1 | Some concerns | Low risk | No concerns | Major concerns | No concerns | No concerns | Very low | ["Within-study bias","Imprecision"] |
| HIIT:MICT | 5 | No concerns | Low risk | No concerns | Major concerns | No concerns | No concerns | Low | ["Imprecision"] |
| HIIT:SOC | 1 | Some concerns | Low risk | No concerns | No concerns | No concerns | No concerns | Moderate | ["Within-study bias"] |
| LICT:MICT | 3 | Some concerns | Low risk | No concerns | No concerns | Major concerns | No concerns | Very low | ["Within-study bias","Heterogeneity"] |
| LICT:SOC | 2 | Some concerns | Low risk | No concerns | Major concerns | No concerns | No concerns | Very low | ["Within-study bias","Imprecision"] |
| MICT:SOC | 7 | Some concerns | Low risk | No concerns | No concerns | Major concerns | No concerns | Very low | ["Within-study bias","Heterogeneity"] |
| Non-training:SOC | 1 | Some concerns | Low risk | No concerns | Major concerns | No concerns | No concerns | Very low | ["Within-study bias","Imprecision"] |
| HICT:HIIT | 0 | Some concerns | Low risk | No concerns | No concerns | Major concerns | No concerns | Very low | ["Within-study bias","Heterogeneity"] |
| HICT:MICT | 0 | Some concerns | Low risk | No concerns | Major concerns | No concerns | No concerns | Very low | ["Within-study bias","Imprecision"] |
| HIIT:LICT | 0 | Some concerns | Low risk | No concerns | No concerns | No concerns | No concerns | Moderate | ["Within-study bias"] |
| HIIT:Non-training | 0 | Some concerns | Low risk | No concerns | No concerns | No concerns | No concerns | Moderate | ["Within-study bias"] |
| LICT:Non-training | 0 | Some concerns | Low risk | No concerns | Major concerns | No concerns | No concerns | Very low | ["Within-study bias","Imprecision"] |
| MICT:Non-training | 0 | Some concerns | Low risk | No concerns | No concerns | Major concerns | No concerns | Very low | ["Within-study bias","Heterogeneity"] |
| Within-study bias：The majority of the primary evidence for most comparisons was derived from studies assessed as having “some concerns” regarding risk of bias. | | | | | | | | | |
| Imprecision：The 95% confidence interval was excessively wide and crossed the predefined threshold for clinical significance. | | | | | | | | | |
| Heterogeneity:The prediction interval for certain comparisons markedly crossed the threshold of clinical significance and extended in both directions. | | | | | | | | | |

Supplementary Table 4.CINeMA-based evaluation of evidence certainty for the 6MWT outcome network.

| **Comparison** | **Number of studies** | **Within-study bias** | **Reporting bias** | **Indirectness** | **Imprecision** | **Heterogeneity** | **Incoherence** | **Confidence rating** | **Reason**  **(s) for downgrading** |
| --- | --- | --- | --- | --- | --- | --- | --- | --- | --- |
| HICT:LICT | 2 | Some concerns | Low risk | No concerns | Major concerns | No concerns | No concerns | Very low | ["Within-study bias","Imprecision"] |
| HICT:MICT | 1 | Some concerns | Low risk | No concerns | Major concerns | No concerns | No concerns | Very low | ["Within-study bias","Imprecision"] |
| HICT:Non-training | 1 | Some concerns | Low risk | No concerns | Major concerns | No concerns | No concerns | Very low | ["Within-study bias","Imprecision"] |
| HICT:SOC | 3 | Some concerns | Low risk | No concerns | Major concerns | No concerns | No concerns | Very low | ["Within-study bias","Imprecision"] |
| HIIT:LICT | 1 | Some concerns | Low risk | No concerns | No concerns | Major concerns | No concerns | Very low | ["Within-study bias","Heterogeneity"] |
| HIIT:MICT | 3 | No concerns | Low risk | No concerns | Major concerns | No concerns | No concerns | Low | ["Imprecision"] |
| LICT:MICT | 2 | Some concerns | Low risk | No concerns | No concerns | Major concerns | No concerns | Very low | ["Within-study bias","Heterogeneity"] |
| LICT:Non-training | 1 | Some concerns | Low risk | No concerns | Major concerns | No concerns | No concerns | Very low | ["Within-study bias","Imprecision"] |
| LICT:SOC | 1 | Some concerns | Low risk | No concerns | Major concerns | No concerns | No concerns | Very low | ["Within-study bias","Imprecision"] |
| MICT:Non-training | 2 | Some concerns | Low risk | No concerns | Major concerns | No concerns | No concerns | Very low | ["Within-study bias","Imprecision"] |
| MICT:SOC | 4 | Some concerns | Low risk | No concerns | No concerns | Major concerns | No concerns | Very low | ["Within-study bias","Heterogeneity"] |
| Non-training:SOC | 1 | Some concerns | Low risk | No concerns | Major concerns | No concerns | No concerns | Very low | ["Within-study bias","Imprecision"] |
| HICT:HIIT | 0 | Some concerns | Low risk | No concerns | Major concerns | No concerns | No concerns | Very low | ["Within-study bias","Imprecision"] |
| HIIT:Non-training | 0 | Some concerns | Low risk | No concerns | No concerns | Major concerns | No concerns | Very low | ["Within-study bias","Heterogeneity"] |
| HIIT:SOC | 0 | Some concerns | Low risk | No concerns | No concerns | Major concerns | No concerns | Very low | ["Within-study bias","Heterogeneity"] |
| Within-study bias：The majority of the primary evidence for most comparisons was derived from studies assessed as having “some concerns” regarding risk of bias. | | | | | | | | | |
| Imprecision：The 95% confidence interval was excessively wide and crossed the predefined threshold for clinical significance. | | | | | | | | | |
| Heterogeneity:The prediction interval for certain comparisons markedly crossed the threshold of clinical significance and extended in both directions. | | | | | | | | | |

Supplementary Table 5.CINeMA-based evaluation of evidence certainty for the 10MWT outcome network.

| **Comparison** | **Number of studies** | **Within-study bias** | **Reporting bias** | **Indirectness** | **Imprecision** | **Heterogeneity** | **Incoherence** | **Confidence rating** | **Reason(s) for downgrading** |
| --- | --- | --- | --- | --- | --- | --- | --- | --- | --- |
| HICT:LICT | 1 | Some concerns | Low risk | No concerns | Major concerns | No concerns | No concerns | Very low | ["Within-study bias","Imprecision"] |
| HICT:MICT | 1 | Some concerns | Low risk | No concerns | Major concerns | No concerns | No concerns | Very low | ["Within-study bias","Imprecision"] |
| HICT:SOC | 1 | Some concerns | Low risk | No concerns | Major concerns | No concerns | No concerns | Very low | ["Within-study bias","Imprecision"] |
| HIIT:LICT | 1 | Some concerns | Low risk | No concerns | No concerns | No concerns | No concerns | Moderate | ["Within-study bias"] |
| HIIT:MICT | 4 | No concerns | Low risk | No concerns | No concerns | No concerns | No concerns | High | / |
| LICT:MICT | 1 | Some concerns | Low risk | No concerns | Major concerns | No concerns | No concerns | Very low | ["Within-study bias","Imprecision"] |
| LICT:Non-training | 1 | Some concerns | Low risk | No concerns | Major concerns | No concerns | No concerns | Very low | ["Within-study bias","Imprecision"] |
| LICT:SOC | 1 | Some concerns | Low risk | No concerns | Major concerns | No concerns | No concerns | Very low | ["Within-study bias","Imprecision"] |
| MICT:SOC | 2 | Some concerns | Low risk | No concerns | No concerns | No concerns | No concerns | Moderate | ["Within-study bias"] |
| HICT:HIIT | 0 | Some concerns | Low risk | No concerns | No concerns | No concerns | No concerns | Moderate | ["Within-study bias"] |
| HICT:Non-training | 0 | Some concerns | Low risk | No concerns | Major concerns | No concerns | No concerns | Very low | ["Within-study bias","Imprecision"] |
| HIIT:Non-training | 0 | Some concerns | Low risk | No concerns | No concerns | No concerns | No concerns | Moderate | ["Within-study bias"] |
| HIIT:SOC | 0 | Some concerns | Low risk | No concerns | No concerns | No concerns | No concerns | Moderate | ["Within-study bias"] |
| MICT:Non-training | 0 | Some concerns | Low risk | No concerns | Major concerns | No concerns | No concerns | Very low | ["Within-study bias","Imprecision"] |
| Non-training:SOC | 0 | Some concerns | Low risk | No concerns | Major concerns | No concerns | No concerns | Very low | ["Within-study bias","Imprecision"] |
| Within-study bias：The majority of the primary evidence for most comparisons was derived from studies assessed as having “some concerns” regarding risk of bias. | | | | | | | | | |
| Imprecision：The 95% confidence interval was excessively wide and crossed the predefined threshold for clinical significance. | | | | | | | | | |

Supplementary Table 6.CINeMA-based evaluation of evidence certainty for the **BBS** outcome network.

| **Comparison** | **Number of studies** | **Within-study bias** | **Reporting bias** | **Indirectness** | **Imprecision** | **Heterogeneity** | **Incoherence** | **Confidence rating** | **Reason**  **(s) for downgrading** |
| --- | --- | --- | --- | --- | --- | --- | --- | --- | --- |
| HICT:LICT | 1 | Some concerns | Low risk | No concerns | Major concerns | No concerns | No concerns | Very low | ["Within-study bias","Imprecision"] |
| HICT:SOC | 1 | Some concerns | Low risk | No concerns | Major concerns | No concerns | No concerns | Very low | ["Within-study bias","Imprecision"] |
| HIIT:MICT | 1 | Some concerns | Low risk | No concerns | Major concerns | No concerns | No concerns | Very low | ["Within-study bias","Imprecision"] |
| LICT:MICT | 1 | Some concerns | Low risk | No concerns | Major concerns | No concerns | No concerns | Very low | ["Within-study bias","Imprecision"] |
| LICT:Non-training | 1 | Some concerns | Low risk | No concerns | Major concerns | No concerns | No concerns | Very low | ["Within-study bias","Imprecision"] |
| LICT:SOC | 2 | Some concerns | Low risk | No concerns | Major concerns | No concerns | No concerns | Very low | ["Within-study bias","Imprecision"] |
| MICT:SOC | 5 | Some concerns | Low risk | No concerns | No concerns | Major concerns | No concerns | Very low | ["Within-study bias","Heterogeneity"] |
| HICT:HIIT | 0 | Some concerns | Low risk | No concerns | Major concerns | No concerns | No concerns | Very low | ["Within-study bias","Imprecision"] |
| HICT:MICT | 0 | Some concerns | Low risk | No concerns | Major concerns | No concerns | No concerns | Very low | ["Within-study bias","Imprecision"] |
| HICT:Non-training | 0 | Some concerns | Low risk | No concerns | Major concerns | No concerns | No concerns | Very low | ["Within-study bias","Imprecision"] |
| HIIT:LICT | 0 | Some concerns | Low risk | No concerns | Major concerns | No concerns | No concerns | Very low | ["Within-study bias","Imprecision"] |
| HIIT:Non-training | 0 | Some concerns | Low risk | No concerns | Major concerns | No concerns | No concerns | Very low | ["Within-study bias","Imprecision"] |
| HIIT:SOC | 0 | Some concerns | Low risk | No concerns | Major concerns | No concerns | No concerns | Very low | ["Within-study bias","Imprecision"] |
| MICT:Non-training | 0 | Some concerns | Low risk | No concerns | Major concerns | No concerns | No concerns | Very low | ["Within-study bias","Imprecision"] |
| Non-training:SOC | 0 | Some concerns | Low risk | No concerns | Major concerns | No concerns | No concerns | Very low | ["Within-study bias","Imprecision"] |
| Within-study bias：The majority of the primary evidence for most comparisons was derived from studies assessed as having “some concerns” regarding risk of bias. | | | | | | | | | |
| Imprecision：The 95% confidence interval was excessively wide and crossed the predefined threshold for clinical significance. | | | | | | | | | |

Supplementary Table 7.CINeMA-based evaluation of evidence certainty for the TUG outcome network.

| **Comparison** | **Number of studies** | **Within-study bias** | **Reporting bias** | **Indirectness** | **Imprecision** | **Heterogeneity** | **Incoherence** | **Confidence rating** | **Reason(s) for downgrading** |
| --- | --- | --- | --- | --- | --- | --- | --- | --- | --- |
| HIIT:LICT | 1 | No concerns | Low risk | No concerns | Major concerns | No concerns | Major concerns | Very low | ["Imprecision","Incoherence"] |
| LICT:SOC | 3 | Some concerns | Low risk | No concerns | Major concerns | No concerns | Major concerns | Very low | ["Within-study bias","Imprecision","Incoherence"] |
| MICT:SOC | 3 | Some concerns | Low risk | No concerns | No concerns | Major concerns | Major concerns | Very low | ["Within-study bias","Heterogeneity","Incoherence"] |
| HIIT:MICT | 0 | Some concerns | Low risk | No concerns | Major concerns | No concerns | Major concerns | Very low | ["Within-study bias","Imprecision","Incoherence"] |
| HIIT:SOC | 0 | No concerns | Low risk | No concerns | Major concerns | No concerns | Major concerns | Very low | ["Imprecision","Incoherence"] |
| LICT:MICT | 0 | Some concerns | Low risk | No concerns | Major concerns | No concerns | Major concerns | Very low | ["Within-study bias","Imprecision","Incoherence"] |
| Within-study bias：The majority of the primary evidence for most comparisons was derived from studies assessed as having “some concerns” regarding risk of bias. | | | | | | | | | |
| Imprecision：The 95% confidence interval was excessively wide and crossed the predefined threshold for clinical significance. | | | | | | | | | |
| Heterogeneity:The prediction interval for certain comparisons markedly crossed the threshold of clinical significance and extended in both directions. | | | | | | | | | |
| Incoherence:Due to the presence of only a single source of evidence and the absence of closed loops, inconsistency testing was not applicable, making it impossible to verify the agreement between direct and indirect evidence. | | | | | | | | | |

Supplementary Table 8.Sensitivity Analysis of the VO_2peak_ outcome

| **dropped_id** | **Comparison** | **eff** | **lci** | **uci** | **connected** |
| --- | --- | --- | --- | --- | --- |
| Boyne P （2016） | SOC_HICT | 0.33088853 | -0.4860411 | 1.147818 | 1 |
| Boyne P （2016） | SOC_HIIT | 1.5851793 | 0.4518022 | 2.718556 | 1 |
| Boyne P （2016） | SOC_LICT | -0.17639143 | -1.123919 | 0.771136 | 1 |
| Boyne P （2016） | SOC_MICT | 1.3496684 | 0.6416075 | 2.057729 | 1 |
| Boyne P （2016） | SOC_Non-training | -0.23218512 | -2.042399 | 1.578029 | 1 |
| Hong J (2013) | SOC_HICT | 0.37702364 | -0.3636916 | 1.117739 | 1 |
| Hong J (2013) | SOC_HIIT | 1.5586851 | 0.572757 | 2.544613 | 1 |
| Hong J (2013) | SOC_LICT | 0.10744315 | -0.8166453 | 1.031532 | 1 |
| Hong J (2013) | SOC_MICT | 1.2109889 | 0.5530874 | 1.86889 | 1 |
| Hong J (2013) | SOC_Non-training | -0.20907907 | -1.857958 | 1.4398 | 1 |
| Lapointe T (2023) | SOC_HICT | 0.34679837 | -0.4665566 | 1.160153 | 1 |
| Lapointe T (2023) | SOC_HIIT | 2.1283804 | 0.8502641 | 3.406497 | 1 |
| Lapointe T (2023) | SOC_LICT | -0.0825305 | -1.038712 | 0.8736506 | 1 |
| Lapointe T (2023) | SOC_MICT | 1.5297885 | 0.7630219 | 2.296555 | 1 |
| Lapointe T (2023) | SOC_Non-training | -0.22428052 | -2.026212 | 1.577651 | 1 |
| Munari D (2018) | SOC_HICT | 0.33084266 | -0.4792182 | 1.140904 | 1 |
| Munari D (2018) | SOC_HIIT | 1.5423553 | 0.4169538 | 2.667757 | 1 |
| Munari D (2018) | SOC_LICT | -0.17552095 | -1.115344 | 0.7643022 | 1 |
| Munari D (2018) | SOC_MICT | 1.352057 | 0.649541 | 2.054573 | 1 |
| Munari D (2018) | SOC_Non-training | -0.2321884 | -2.028205 | 1.563828 | 1 |
| Tang A （2014） | SOC_HICT | 0.37434894 | -0.5366046 | 1.285303 | 1 |
| Tang A （2014） | SOC_HIIT | 1.683553 | 0.5950628 | 2.772043 | 1 |
| Tang A （2014） | SOC_LICT | -0.1692828 | -1.132903 | 0.7943378 | 1 |
| Tang A （2014） | SOC_MICT | 1.3459331 | 0.6293871 | 2.062479 | 1 |
| Tang A （2014） | SOC_Non-training | -0.21064793 | -2.053251 | 1.631955 | 1 |
| Globas C （2012） | SOC_HICT | 0.21646677 | -0.6783273 | 1.111261 | 1 |
| Globas C （2012） | SOC_HIIT | 1.671648 | 0.5975105 | 2.745785 | 1 |
| Globas C （2012） | SOC_LICT | -0.20707888 | -1.157419 | 0.743261 | 1 |
| Globas C （2012） | SOC_MICT | 1.3332659 | 0.626157 | 2.040375 | 1 |
| Globas C （2012） | SOC_Non-training | -0.28895363 | -2.106836 | 1.528929 | 1 |
| Hornby TG （2019） | SOC_HICT | 0.40980236 | -0.4731627 | 1.292768 | 1 |
| Hornby TG （2019） | SOC_HIIT | 1.6503912 | 0.5645376 | 2.736245 | 1 |
| Hornby TG （2019） | SOC_LICT | -0.2951108 | -1.357626 | 0.7674048 | 1 |
| Hornby TG （2019） | SOC_MICT | 1.3083979 | 0.5848928 | 2.031903 | 1 |
| Hornby TG （2019） | SOC_Non-training | -0.19303872 | -2.017615 | 1.631537 | 1 |
| Ivey FM （2010） | SOC_HICT | 0.33869785 | -0.4792624 | 1.156658 | 1 |
| Ivey FM （2010） | SOC_HIIT | 1.7617922 | 0.6601707 | 2.863414 | 1 |
| Ivey FM （2010） | SOC_LICT | -0.13074466 | -1.089584 | 0.8280944 | 1 |
| Ivey FM （2010） | SOC_MICT | 1.4370021 | 0.6781268 | 2.195878 | 1 |
| Ivey FM （2010） | SOC_Non-training | -0.22831231 | -2.039958 | 1.583333 | 1 |
| Ivey FM （2007） | SOC_HICT | 0.31634685 | -0.4738916 | 1.106585 | 1 |
| Ivey FM （2007） | SOC_HIIT | 1.5527887 | 0.4876985 | 2.617879 | 1 |
| Ivey FM （2007） | SOC_LICT | -0.25680852 | -1.183694 | 0.6700767 | 1 |
| Ivey FM （2007） | SOC_MICT | 1.1987678 | 0.4671901 | 1.930346 | 1 |
| Ivey FM （2007） | SOC_Non-training | -0.23931717 | -1.993724 | 1.515089 | 1 |
| Lee MJ （2008） | SOC_HICT | 0.26889662 | -0.6297234 | 1.167517 | 1 |
| Lee MJ （2009） | SOC_HIIT | 1.6764241 | 0.5934377 | 2.759411 | 1 |
| Lee MJ （2010） | SOC_LICT | -0.19403266 | -1.15226 | 0.7641948 | 1 |
| Lee MJ （2011） | SOC_MICT | 1.3381168 | 0.6252129 | 2.051021 | 1 |
| Macko RF （2005） | SOC_HICT | 0.35470837 | -0.4558034 | 1.16522 | 1 |
| Macko RF （2005） | SOC_HIIT | 1.628698 | 0.5581883 | 2.699208 | 1 |
| Macko RF （2005） | SOC_LICT | -0.03544391 | -1.039858 | 0.9689705 | 1 |
| Macko RF （2005） | SOC_MICT | 1.2845159 | 0.5713938 | 1.997638 | 1 |
| Macko RF （2005） | SOC_Non-training | -0.22034372 | -2.012753 | 1.572066 | 1 |
| Quaney BM （2009) | SOC_HICT | 0.34334964 | -0.4450313 | 1.131731 | 1 |
| Quaney BM （2009) | SOC_HIIT | 1.8155281 | 0.7517519 | 2.879304 | 1 |
| Quaney BM （2009) | SOC_LICT | -0.09852003 | -1.023593 | 0.8265526 | 1 |
| Quaney BM （2009) | SOC_MICT | 1.5018035 | 0.7698086 | 2.233798 | 1 |
| Quaney BM （2009) | SOC_Non-training | -0.22592075 | -1.976531 | 1.524689 | 1 |
| Serra MC (2019） | SOC_HICT | 0.28678788 | -0.3318621 | 0.9054379 | 1 |
| Serra MC (2019） | SOC_HIIT | 1.3530452 | 0.514856 | 2.191234 | 1 |
| Serra MC (2019） | SOC_LICT | -0.3821952 | -1.110625 | 0.3462344 | 1 |
| Serra MC (2019） | SOC_MICT | 0.98999059 | 0.4248733 | 1.555108 | 1 |
| Serra MC (2019） | SOC_Non-training | -0.25315064 | -1.658348 | 1.152046 | 1 |
| Moore SA （2015） | SOC_HICT | 0.33966655 | -0.4734389 | 1.152772 | 1 |
| Moore SA （2015） | SOC_HIIT | 1.7725644 | 0.6776289 | 2.8675 | 1 |
| Moore SA （2015） | SOC_LICT | -0.12428385 | -1.077386 | 0.8288185 | 1 |
| Moore SA （2015） | SOC_MICT | 1.4498654 | 0.6963673 | 2.203363 | 1 |
| Moore SA （2015） | SOC_Non-training | -0.22781839 | -2.029446 | 1.57381 | 1 |
| Doğan Duran Ü （2022） | SOC_HICT | 0.30692358 | -0.51443 | 1.128277 | 1 |
| Doğan Duran Ü （2022） | SOC_HIIT | 1.644202 | 0.5619454 | 2.726459 | 1 |
| Doğan Duran Ü （2022） | SOC_LICT | -0.31679209 | -1.388517 | 0.7549326 | 1 |
| Doğan Duran Ü （2022） | SOC_MICT | 1.3015849 | 0.5793938 | 2.023776 | 1 |
| Doğan Duran Ü （2022） | SOC_Non-training | -0.2440753 | -2.054652 | 1.566501 | 1 |
| Rimmer JH （2009） | SOC_HICT | 0.28627954 | -0.4849673 | 1.057526 | 1 |
| Rimmer JH （2009） | SOC_HIIT | 1.799161 | 0.7625691 | 2.835753 | 1 |
| Rimmer JH （2009） | SOC_LICT | -0.42792973 | -1.445914 | 0.5900546 | 1 |
| Rimmer JH （2009） | SOC_MICT | 1.4852032 | 0.7729099 | 2.197497 | 1 |
| Rimmer JH （2009） | SOC_Non-training | -0.25414316 | -1.961031 | 1.452745 | 1 |
| Thompson ED （2023） | SOC_HICT | 0.38683437 | -0.5311021 | 1.304771 | 1 |
| Thompson ED （2023） | SOC_HIIT | 1.684425 | 0.5951272 | 2.773723 | 1 |
| Thompson ED （2023） | SOC_LICT | -0.16633757 | -1.130967 | 0.798292 | 1 |
| Thompson ED （2023） | SOC_MICT | 1.3468801 | 0.6297901 | 2.06397 | 1 |
| Thompson ED （2023） | SOC_Non-training | -0.20445355 | -2.049185 | 1.640277 | 1 |
| Linder SM （2024） | SOC_HICT | 0.32592227 | -0.5871714 | 1.239016 | 1 |
| Linder SM （2024） | SOC_HIIT | 1.6811005 | 0.5908794 | 2.771322 | 1 |
| Linder SM （2024） | SOC_LICT | -0.1801658 | -1.145408 | 0.7850767 | 1 |
| Linder SM （2024） | SOC_MICT | 1.3429856 | 0.6252863 | 2.060685 | 1 |
| Linder SM （2024） | SOC_Non-training | -0.23468366 | -2.080256 | 1.610889 | 1 |
| Boyne P （2025） | SOC_HICT | 0.33078516 | -0.4982586 | 1.159829 | 1 |
| Boyne P （2025） | SOC_HIIT | 1.6804584 | 0.5122686 | 2.848648 | 1 |
| Boyne P （2025） | SOC_LICT | -0.17894149 | -1.140102 | 0.7822188 | 1 |
| Boyne P （2025） | SOC_MICT | 1.3435527 | 0.6254852 | 2.06162 | 1 |
| Boyne P （2025） | SOC_Non-training | -0.23226963 | -2.067543 | 1.603004 | 1 |
| Do J (2025) | SOC_HICT | 0.33062678 | -0.4979574 | 1.159211 | 1 |
| Do J (2025) | SOC_HIIT | 1.6968971 | 0.5310282 | 2.862766 | 1 |
| Do J (2025) | SOC_LICT | -0.17979807 | -1.140441 | 0.7808446 | 1 |
| Do J (2025) | SOC_MICT | 1.3419577 | 0.6242763 | 2.059639 | 1 |
| Do J (2025) | SOC_Non-training | -0.23234696 | -2.066669 | 1.601975 | 1 |

Supplementary Table 9.Sensitivity Analysis of the 6MWT outcome

| **Dropped_id** | **comparison** | **eff** | **lci** | **uci** | **connected** |
| --- | --- | --- | --- | --- | --- |
| Boyne P （2016） | SOC_HICT | 0.34111053 | -0.0476351 | 0.7298561 | 1 |
| Boyne P （2016） | SOC_HIIT | 0.98234281 | 0.3125445 | 1.652141 | 1 |
| Boyne P （2016） | SOC_LICT | -0.02200194 | -0.4859409 | 0.441937 | 1 |
| Boyne P （2016） | SOC_MICT | 0.45468985 | 0.0547664 | 0.8546133 | 1 |
| Boyne P （2016） | SOC_Non-training | 0.13210064 | -0.4021654 | 0.6663666 | 1 |
| Boyne P （2023） | SOC_HICT | 0.33742692 | -0.0597578 | 0.7346116 | 1 |
| Boyne P （2023） | SOC_HIIT | 0.73730071 | 0.0279381 | 1.446663 | 1 |
| Boyne P （2023） | SOC_LICT | -0.0399502 | -0.5126216 | 0.4327212 | 1 |
| Boyne P （2023） | SOC_MICT | 0.46513551 | 0.0578704 | 0.8724006 | 1 |
| Boyne P （2023） | SOC_Non-training | 0.13078043 | -0.4137868 | 0.6753476 | 1 |
| Hong J (2013) | SOC_HICT | 0.32059805 | -0.0535671 | 0.6947632 | 1 |
| Hong J (2013) | SOC_HIIT | 0.89898895 | 0.2968443 | 1.501134 | 1 |
| Hong J (2013) | SOC_LICT | -0.1376142 | -0.6174209 | 0.3421925 | 1 |
| Hong J (2013) | SOC_MICT | 0.50963808 | 0.1149089 | 0.9043673 | 1 |
| Hong J (2013) | SOC_Non-training | 0.12002215 | -0.3952648 | 0.6353091 | 1 |
| Munari D (2018) | SOC_HICT | 0.33260977 | -0.0592216 | 0.7244412 | 1 |
| Munari D (2018) | SOC_HIIT | 0.97087655 | 0.3002125 | 1.641541 | 1 |
| Munari D (2018) | SOC_LICT | -0.06690034 | -0.5411239 | 0.4073232 | 1 |
| Munari D (2018) | SOC_MICT | 0.47760301 | 0.0727661 | 0.8824399 | 1 |
| Munari D (2018) | SOC_Non-training | 0.12785936 | -0.4097617 | 0.6654804 | 1 |
| Tang A （2014) | SOC_HICT | 0.40115085 | -0.0408244 | 0.8431261 | 1 |
| Tang A （2014) | SOC_HIIT | 0.89556765 | 0.2574029 | 1.533732 | 1 |
| Tang A （2014) | SOC_LICT | 0.00617279 | -0.4797531 | 0.4920987 | 1 |
| Tang A （2014) | SOC_MICT | 0.48765866 | 0.0712659 | 0.9040514 | 1 |
| Tang A （2014) | SOC_Non-training | 0.16907233 | -0.3885736 | 0.7267183 | 1 |
| Globas C （2012） | SOC_HICT | 0.34141102 | -0.1006543 | 0.7834764 | 1 |
| Globas C （2012） | SOC_HIIT | 0.8668312 | 0.2229105 | 1.510752 | 1 |
| Globas C （2012） | SOC_LICT | -0.02802292 | -0.5176547 | 0.4616088 | 1 |
| Globas C （2012） | SOC_MICT | 0.46170264 | 0.0420145 | 0.8813908 | 1 |
| Globas C （2012） | SOC_Non-training | 0.13340086 | -0.4289733 | 0.695775 | 1 |
| Gordon CD （2013） | SOC_HICT | 0.33439528 | -0.0859284 | 0.7547189 | 1 |
| Gordon CD （2013） | SOC_HIIT | 0.86746376 | 0.222517 | 1.512411 | 1 |
| Gordon CD （2013） | SOC_LICT | -0.02735795 | -0.5104833 | 0.4557674 | 1 |
| Gordon CD （2013） | SOC_MICT | 0.4635016 | 0.0451333 | 0.8818699 | 1 |
| Gordon CD （2013） | SOC_Non-training | 0.14733247 | -0.5037187 | 0.7983837 | 1 |
| Hornby TG （2019） | SOC_HICT | 0.27590649 | -0.0984543 | 0.6502674 | 1 |
| Hornby TG （2019） | SOC_HIIT | 0.92646676 | 0.3324015 | 1.520532 | 1 |
| Hornby TG （2019） | SOC_LICT | 0.10066819 | -0.3673003 | 0.5686367 | 1 |
| Hornby TG （2019） | SOC_MICT | 0.49536543 | 0.1119405 | 0.8787903 | 1 |
| Hornby TG （2019） | SOC_Non-training | 0.14738526 | -0.3596562 | 0.6544267 | 1 |
| Lee MJ （2008） | SOC_HICT | 0.36250855 | -0.0611964 | 0.7862135 | 1 |
| Lee MJ （2008） | SOC_HIIT | 0.91989163 | 0.2520808 | 1.587703 | 1 |
| Lee MJ （2008） | SOC_LICT | 0.00829636 | -0.4962698 | 0.5128625 | 1 |
| Lee MJ （2008） | SOC_MICT | 0.52031003 | 0.0670725 | 0.9735475 | 1 |
| Lee MJ （2008） | SOC_Non-training | 0.16184596 | -0.4945432 | 0.8182351 | 1 |
| Macko RF （2005） | SOC_HICT | 0.36576944 | 0.0997577 | 0.6317812 | 1 |
| Macko RF （2005） | SOC_HIIT | 0.83386982 | 0.3677836 | 1.299956 | 1 |
| Macko RF （2005） | SOC_LICT | 0.12780812 | -0.224123 | 0.4797393 | 1 |
| Macko RF （2005） | SOC_MICT | 0.34413023 | 0.0386773 | 0.6495832 | 1 |
| Macko RF （2005） | SOC_Non-training | 0.13540052 | -0.2477152 | 0.5185162 | 1 |
| Moore SA （2015) | SOC_HICT | 0.33741236 | -0.0737401 | 0.7485648 | 1 |
| Moore SA （2015) | SOC_HIIT | 0.86185668 | 0.1980423 | 1.525671 | 1 |
| Moore SA （2015) | SOC_LICT | -0.03208301 | -0.5267546 | 0.4625886 | 1 |
| Moore SA （2015) | SOC_MICT | 0.45647572 | 0.0007305 | 0.912221 | 1 |
| Moore SA （2015) | SOC_Non-training | 0.12945217 | -0.4350444 | 0.6939487 | 1 |
| Liu-Ambrose T （2015) | SOC_HICT | 0.36600478 | -0.0352391 | 0.7672487 | 1 |
| Liu-Ambrose T （2015) | SOC_HIIT | 0.92633702 | 0.2797945 | 1.57288 | 1 |
| Liu-Ambrose T （2015) | SOC_LICT | 0.01093601 | -0.4714819 | 0.4933539 | 1 |
| Liu-Ambrose T （2015) | SOC_MICT | 0.52255344 | 0.0844072 | 0.9606996 | 1 |
| Liu-Ambrose T （2015) | SOC_Non-training | 0.17036562 | -0.3813629 | 0.7220942 | 1 |
| Yeh TT （2019） | SOC_HICT | 0.27777218 | -0.0906879 | 0.6462322 | 1 |
| Yeh TT （2019） | SOC_HIIT | 0.73456518 | 0.1305654 | 1.338565 | 1 |
| Yeh TT （2019） | SOC_LICT | -0.12982878 | -0.577363 | 0.3177054 | 1 |
| Yeh TT （2019） | SOC_MICT | 0.30920571 | -0.0973619 | 0.7157733 | 1 |
| Yeh TT （2019） | SOC_Non-training | 0.03968957 | -0.470998 | 0.5503771 | 1 |
| Yeh TT （2022） | SOC_HICT | 0.32796413 | -0.0691338 | 0.7250621 | 1 |
| Yeh TT （2022） | SOC_HIIT | 0.8907452 | 0.2584153 | 1.523075 | 1 |
| Yeh TT （2022） | SOC_LICT | -0.03236695 | -0.503144 | 0.4384101 | 1 |
| Yeh TT （2022） | SOC_MICT | 0.48875414 | 0.0755694 | 0.9019389 | 1 |
| Yeh TT （2022） | SOC_Non-training | 0.04305734 | -0.5490917 | 0.6352063 | 1 |
| Doğan Duran Ü （2022) | SOC_HICT | 0.31945218 | -0.0911976 | 0.7301019 | 1 |
| Doğan Duran Ü （2022) | SOC_HIIT | 0.83916272 | 0.1919796 | 1.486346 | 1 |
| Doğan Duran Ü （2022) | SOC_LICT | -0.07782878 | -0.608266 | 0.4526084 | 1 |
| Doğan Duran Ü （2022) | SOC_MICT | 0.43691439 | 0.0125487 | 0.8612801 | 1 |
| Doğan Duran Ü （2022) | SOC_Non-training | 0.1073615 | -0.4523632 | 0.6670862 | 1 |
| Mberti NLA （2017) | SOC_HICT | 0.38722096 | 0.0020335 | 0.7724084 | 1 |
| Mberti NLA （2017) | SOC_HIIT | 0.83076994 | 0.2215863 | 1.439954 | 1 |
| Mberti NLA （2017) | SOC_LICT | -0.12879625 | -0.6040909 | 0.3464984 | 1 |
| Mberti NLA （2017) | SOC_MICT | 0.42923999 | 0.0355196 | 0.8229604 | 1 |
| Mberti NLA （2017) | SOC_Non-training | 0.11752736 | -0.404483 | 0.6395378 | 1 |
| Thompson ED （2023） | SOC_HICT | 0.34480858 | -0.1294102 | 0.8190274 | 1 |
| Thompson ED （2023） | SOC_HIIT | 0.86752814 | 0.2124095 | 1.522647 | 1 |
| Thompson ED （2023） | SOC_LICT | -0.02546666 | -0.5288896 | 0.4779563 | 1 |
| Thompson ED （2023） | SOC_MICT | 0.46382734 | 0.0340564 | 0.8935983 | 1 |
| Thompson ED （2023） | SOC_Non-training | 0.13578274 | -0.4407903 | 0.7123557 | 1 |
| Moncion K （2024） | SOC_HICT | 0.32142834 | -0.1033298 | 0.7461866 | 1 |
| Moncion K （2024） | SOC_HIIT | 0.88173566 | 0.229303 | 1.534168 | 1 |
| Moncion K （2024） | SOC_LICT | -0.02587483 | -0.507322 | 0.4555724 | 1 |
| Moncion K （2024） | SOC_MICT | 0.48006688 | 0.0444771 | 0.9156566 | 1 |
| Moncion K （2024） | SOC_Non-training | 0.13365666 | -0.4215091 | 0.6888224 | 1 |
| Boyne P （2025） | SOC_HICT | 0.3377613 | -0.0627356 | 0.7382582 | 1 |
| Boyne P （2025） | SOC_HIIT | 0.76497444 | 0.0503201 | 1.479629 | 1 |
| Boyne P （2025） | SOC_LICT | -0.03747787 | -0.5136194 | 0.4386637 | 1 |
| Boyne P （2025） | SOC_MICT | 0.46444658 | 0.0543809 | 0.8745123 | 1 |
| Boyne P （2025） | SOC_Non-training | 0.13117936 | -0.4174533 | 0.679812 | 1 |
| Palmcrantz S (2021) | SOC_HICT | 0.34089109 | -0.054143 | 0.7359252 | 1 |
| Palmcrantz S (2021) | SOC_HIIT | 0.8569574 | 0.2285059 | 1.485409 | 1 |
| Palmcrantz S (2021) | SOC_LICT | -0.07892575 | -0.5660728 | 0.4082213 | 1 |
| Palmcrantz S (2021) | SOC_MICT | 0.45711506 | 0.0517669 | 0.8624633 | 1 |
| Palmcrantz S (2021) | SOC_Non-training | 0.21338499 | -0.3703494 | 0.7971194 | 1 |

Supplementary Table 10.Sensitivity Analysis of the 10MWT outcome

| **Dropped_id** | **comparison** | **eff** | **lci** | **uci** | **connected** |
| --- | --- | --- | --- | --- | --- |
| Boyne P （2016） | SOC_HICT | 0.33219497 | -0.038653 | 0.703043 | 1 |
| Boyne P （2016） | SOC_HIIT | 1.3071768 | 0.8415777 | 1.772776 | 1 |
| Boyne P （2016） | SOC_LICT | 0.3599222 | -0.0616138 | 0.7814582 | 1 |
| Boyne P （2016） | SOC_MICT | 0.61426559 | 0.259664 | 0.9688672 | 1 |
| Boyne P （2016） | SOC_Non-training | 0.35989971 | -0.4609913 | 1.180791 | 1 |
| Boyne P （2023） | SOC_HICT | 0.33219589 | -0.0386521 | 0.7030439 | 1 |
| Boyne P （2023） | SOC_HIIT | 1.398796 | 0.9088081 | 1.888784 | 1 |
| Boyne P （2023） | SOC_LICT | 0.36831299 | -0.0534548 | 0.7900807 | 1 |
| Boyne P （2023） | SOC_MICT | 0.61066284 | 0.2560104 | 0.9653153 | 1 |
| Boyne P （2023） | SOC_Non-training | 0.36828997 | -0.45272 | 1.1893 | 1 |
| Munari D (2018) | SOC_HICT | 0.33217288 | -0.0386751 | 0.7030209 | 1 |
| Munari D (2018) | SOC_HIIT | 1.4465169 | 0.975326 | 1.917708 | 1 |
| Munari D (2018) | SOC_LICT | 0.32357363 | -0.1076944 | 0.7548416 | 1 |
| Munari D (2018) | SOC_MICT | 0.62983347 | 0.2730912 | 0.9865757 | 1 |
| Munari D (2018) | SOC_Non-training | 0.32355341 | -0.5023767 | 1.149483 | 1 |
| Globas C （2012） | SOC_HICT | 0.43830808 | -0.1029746 | 0.9795907 | 1 |
| Globas C （2012） | SOC_HIIT | 1.4457428 | 0.9421276 | 1.949358 | 1 |
| Globas C （2012） | SOC_LICT | 0.42557831 | -0.0482938 | 0.8994504 | 1 |
| Globas C （2012） | SOC_MICT | 0.66936936 | 0.2538902 | 1.084849 | 1 |
| Globas C （2012） | SOC_Non-training | 0.42555171 | -0.4233996 | 1.274503 | 1 |
| Macko RF （2005) | SOC_HICT | 0.3322025 | -0.0386455 | 0.7030505 | 1 |
| Macko RF （2005) | SOC_HIIT | 1.3758697 | 0.9144406 | 1.837299 | 1 |
| Macko RF （2005) | SOC_LICT | 0.40502369 | -0.0881145 | 0.8981619 | 1 |
| Macko RF （2005) | SOC_MICT | 0.59490593 | 0.2237246 | 0.9660873 | 1 |
| Macko RF （2005) | SOC_Non-training | 0.40499838 | -0.4548542 | 1.264851 | 1 |
| Moore SA （2015） | SOC_HICT | 0.33749682 | -0.0601558 | 0.7351494 | 1 |
| Moore SA （2015） | SOC_HIIT | 1.396071 | 0.8853629 | 1.906779 | 1 |
| Moore SA （2015） | SOC_LICT | 0.37336759 | -0.0791843 | 0.8259194 | 1 |
| Moore SA （2015） | SOC_MICT | 0.61993066 | 0.1923919 | 1.047469 | 1 |
| Moore SA （2015） | SOC_Non-training | 0.37334426 | -0.4638948 | 1.210583 | 1 |
| Kim J （2017) | SOC_HICT | 0.34804085 | -0.0436485 | 0.7397302 | 1 |
| Kim J （2017) | SOC_HIIT | 1.4072891 | 0.9260557 | 1.888523 | 1 |
| Kim J （2017) | SOC_LICT | 0.40453607 | -0.1106147 | 0.9196869 | 1 |
| Kim J （2017) | SOC_MICT | 0.62929429 | 0.246301 | 1.012288 | 1 |
| Kim J （2017) | SOC_Non-training | 0.40451079 | -0.4681512 | 1.277173 | 1 |
| Kim SJ （2015) | SOC_HICT | 0.27762341 | -0.1115142 | 0.666761 | 1 |
| Kim SJ （2015) | SOC_HIIT | 1.2990502 | 0.8058653 | 1.792235 | 1 |
| Kim SJ （2015) | SOC_LICT | 0.30457336 | -0.1380988 | 0.7472456 | 1 |
| Kim SJ （2015) | SOC_MICT | 0.52027936 | 0.115025 | 0.9255338 | 1 |
| Kim SJ （2015) | SOC_Non-training | 0.30455433 | -0.5273867 | 1.136495 | 1 |
| Mberti NLA （2017） | SOC_HICT | 0.32429555 | -0.0648879 | 0.713479 | 1 |
| Mberti NLA （2017） | SOC_HIIT | 1.3898745 | 0.933762 | 1.845987 | 1 |
| Mberti NLA （2017） | SOC_LICT | 0.38026167 | -0.0838589 | 0.8443823 | 1 |
| Mberti NLA （2017） | SOC_MICT | 0.61251649 | 0.2572921 | 0.9677409 | 1 |
| Mberti NLA （2017） | SOC_Non-training | 0.3802379 | -0.4633098 | 1.223786 | 1 |
| Moncion K （2024) | SOC_HICT | 0.28903071 | -0.1442083 | 0.7222698 | 1 |
| Moncion K （2024) | SOC_HIIT | 1.4200916 | 0.934904 | 1.905279 | 1 |
| Moncion K （2024) | SOC_LICT | 0.375073 | -0.0483064 | 0.7984524 | 1 |
| Moncion K （2024) | SOC_MICT | 0.64602783 | 0.2477757 | 1.04428 | 1 |
| Moncion K （2024) | SOC_Non-training | 0.37504956 | -0.4467895 | 1.196889 | 1 |
| Boyne P （2025） | SOC_HICT | 0.33219589 | -0.0386521 | 0.7030439 | 1 |
| Boyne P （2025） | SOC_HIIT | 1.3276773 | 0.8395246 | 1.81583 | 1 |
| Boyne P （2025） | SOC_LICT | 0.36180039 | -0.0599495 | 0.7835503 | 1 |
| Boyne P （2025） | SOC_MICT | 0.61346066 | 0.2588122 | 0.9681092 | 1 |
| Boyne P （2025） | SOC_Non-training | 0.36177778 | -0.4592231 | 1.182779 | 1 |
| Do J (2025) | SOC_HICT | 0.33219603 | -0.038652 | 0.703044 | 1 |
| Do J (2025) | SOC_HIIT | 1.4808095 | 0.9982949 | 1.963324 | 1 |
| Do J (2025) | SOC_LICT | 0.37582331 | -0.0458722 | 0.7975188 | 1 |
| Do J (2025) | SOC_MICT | 0.60743663 | 0.2528 | 0.9620732 | 1 |
| Do J (2025) | SOC_Non-training | 0.37579983 | -0.4451731 | 1.196773 | 1 |
| Palmcrantz S (2021) | SOC_HICT | 0.3321836 | -0.0386644 | 0.7030316 | 1 |
| Palmcrantz S (2021) | SOC_HIIT | 1.3874586 | 0.9327813 | 1.842136 | 1 |
| Palmcrantz S (2021) | SOC_LICT | 0.3672714 | -0.0541719 | 0.7887146 | 1 |
| Palmcrantz S (2021) | SOC_MICT | 0.61108379 | 0.256507 | 0.9656606 | 1 |

Supplementary Table 11.Sensitivity Analysis of the BBS outcome

| **Dropped_id** | **comparison** | **eff** | **lci** | **uci** | **connected** |
| --- | --- | --- | --- | --- | --- |
| Hong J (2013) | SOC_HICT | 0.23873804 | -0.399817 | 0.8772932 | 1 |
| Hong J (2013) | SOC_HIIT | 0.81961258 | -0.0409171 | 1.680142 | 1 |
| Hong J (2013) | SOC_LICT | 0.40622781 | -0.1286037 | 0.9410594 | 1 |
| Hong J (2013) | SOC_MICT | 0.55436564 | 0.1669888 | 0.9417425 | 1 |
| Hong J (2013) | SOC_Non-training | 0.36535745 | -0.6446462 | 1.375361 | 1 |
| Globas C （2012） | SOC_HICT | 0.2544859 | -0.6700162 | 1.178988 | 1 |
| Globas C （2012） | SOC_HIIT | 0.78412139 | -0.0408887 | 1.609131 | 1 |
| Globas C （2012） | SOC_LICT | 0.4675529 | 0.0161189 | 0.918987 | 1 |
| Globas C （2012） | SOC_MICT | 0.51886925 | 0.1734473 | 0.8642912 | 1 |
| Globas C （2012） | SOC_Non-training | 0.4266785 | -0.5266037 | 1.379961 | 1 |
| Quaney BM （2009) | SOC_HICT | 0.30939386 | -0.1936475 | 0.8124352 | 1 |
| Quaney BM （2009) | SOC_HIIT | 0.87132837 | 0.1946568 | 1.548 | 1 |
| Quaney BM （2009) | SOC_LICT | 0.54887888 | 0.184157 | 0.9136007 | 1 |
| Quaney BM （2009) | SOC_MICT | 0.60606296 | 0.2885638 | 0.9235622 | 1 |
| Quaney BM （2009) | SOC_Non-training | 0.50801124 | -0.2880783 | 1.304101 | 1 |
| Moore SA （2015） | SOC_HICT | 0.26472874 | -0.3272539 | 0.8567114 | 1 |
| Moore SA （2015） | SOC_HIIT | 0.75279213 | -0.0661201 | 1.571704 | 1 |
| Moore SA （2015） | SOC_LICT | 0.45859477 | 0.0449648 | 0.8722248 | 1 |
| Moore SA （2015） | SOC_MICT | 0.48753538 | 0.1159478 | 0.8591229 | 1 |
| Moore SA （2015） | SOC_Non-training | 0.41772282 | -0.5027484 | 1.338194 | 1 |
| Liu-Ambrose T （2015） | SOC_HICT | 0.30215948 | -0.2103487 | 0.8146677 | 1 |
| Liu-Ambrose T （2015） | SOC_HIIT | 0.85820586 | 0.1630309 | 1.553381 | 1 |
| Liu-Ambrose T （2015） | SOC_LICT | 0.53421099 | 0.1817943 | 0.8866277 | 1 |
| Liu-Ambrose T （2015） | SOC_MICT | 0.59294205 | 0.2722999 | 0.9135842 | 1 |
| Liu-Ambrose T （2015） | SOC_Non-training | 0.49334272 | -0.3118324 | 1.298518 | 1 |
| Doğan Duran Ü （2022） | SOC_HICT | 0.32025299 | -0.2232919 | 0.8637978 | 1 |
| Doğan Duran Ü （2022） | SOC_HIIT | 0.81279136 | 0.0887408 | 1.536842 | 1 |
| Doğan Duran Ü （2022） | SOC_LICT | 0.57074138 | 0.1711795 | 0.9703033 | 1 |
| Doğan Duran Ü （2022） | SOC_MICT | 0.54752955 | 0.2380473 | 0.8570118 | 1 |
| Doğan Duran Ü （2022） | SOC_Non-training | 0.52986726 | -0.3255564 | 1.385291 | 1 |
| Mustafaoğlu R （2018） | SOC_HICT | 0.25495455 | -0.2392429 | 0.7491521 | 1 |
| Mustafaoğlu R （2019） | SOC_HIIT | 0.68828246 | 0.0251669 | 1.351398 | 1 |
| Mustafaoğlu R （2020） | SOC_LICT | 0.43865901 | 0.1135112 | 0.7638068 | 1 |
| Mustafaoğlu R （2021） | SOC_MICT | 0.42300822 | 0.1279521 | 0.7180643 | 1 |
| Mustafaoğlu R （2022） | SOC_Non-training | 0.39779858 | -0.3781019 | 1.173699 | 1 |
| Kim SJ （2015） | SOC_HICT | 0.25458246 | -0.2397089 | 0.7488738 | 1 |
| Kim SJ （2015） | SOC_HIIT | 0.68704834 | 0.0231632 | 1.350933 | 1 |
| Kim SJ （2015） | SOC_LICT | 0.43790562 | 0.1121734 | 0.7636379 | 1 |
| Kim SJ （2015） | SOC_MICT | 0.42177405 | 0.1249924 | 0.7185557 | 1 |
| Kim SJ （2015） | SOC_Non-training | 0.39704524 | -0.3791003 | 1.173191 | 1 |
| Mberti NLA （2017） | SOC_HICT | 0.28359987 | -0.5166193 | 1.083819 | 1 |
| Mberti NLA （2017） | SOC_HIIT | 0.78412806 | -0.0409042 | 1.60916 | 1 |
| Mberti NLA （2017） | SOC_LICT | 0.46756971 | 0.0161153 | 0.9190241 | 1 |
| Mberti NLA （2017） | SOC_MICT | 0.51887593 | 0.1734444 | 0.8643075 | 1 |
| Mberti NLA （2017） | SOC_Non-training | 0.42669531 | -0.5266122 | 1.380003 | 1 |
| Do J (2025) | SOC_HICT | 0.27640327 | -0.2596469 | 0.8124534 | 1 |
| Do J (2025) | SOC_HIIT | 0.4821166 | 0.1182147 | 0.8460185 | 1 |
| Do J (2025) | SOC_LICT | 0.51418971 | 0.2108487 | 0.8175307 | 1 |
| Do J (2025) | SOC_MICT | 0.44124906 | -0.3964142 | 1.278912 | 1 |
| Wu C (2025) | SOC_HICT | 0.24718291 | -0.3749611 | 0.8693269 | 1 |
| Wu C (2025) | SOC_HIIT | 0.77096247 | -0.0601261 | 1.602051 | 1 |
| Wu C (2025) | SOC_LICT | 0.42323026 | -0.0777505 | 0.924211 | 1 |
| Wu C (2025) | SOC_MICT | 0.50570979 | 0.1530852 | 0.8583344 | 1 |
| Wu C (2025) | SOC_Non-training | 0.38235957 | -0.5979304 | 1.36265 | 1 |
| Palmcrantz S (2021) | SOC_HICT | 0.27640734 | -0.2596381 | 0.8124528 | 1 |
| Palmcrantz S (2021) | SOC_HIIT | 0.77944363 | 0.0599882 | 1.498899 | 1 |
| Palmcrantz S (2021) | SOC_LICT | 0.48212482 | 0.1182257 | 0.846024 | 1 |
| Palmcrantz S (2021) | SOC_MICT | 0.51417972 | 0.2108466 | 0.8175129 | 1 |

Supplementary Table 12.Sensitivity Analysis of the TUG outcome

| **Dropped_id** | **comparison** | **eff** | **lci** | **uci** | **connected** |
| --- | --- | --- | --- | --- | --- |
| Mustafaoğlu R （2018） | SOC_HIIT | -0.48352827 | -1.821984 | 0.8549275 | 1 |
| Mustafaoğlu R （2019） | SOC_LICT | -0.36344406 | -0.9161497 | 0.1892616 | 1 |
| Mustafaoğlu R （2020） | SOC_MICT | -0.38158843 | -1.087577 | 0.3244 | 1 |
| Kim SJ （2015） | SOC_HIIT | -0.52164798 | -2.196975 | 1.153679 | 1 |
| Kim SJ （2015） | SOC_LICT | -0.40161722 | -1.145845 | 0.3426106 | 1 |
| Kim SJ （2015） | SOC_MICT | -0.67411835 | -1.632209 | 0.2839727 | 1 |
| Au-Yeung SSY （2009） | SOC_HIIT | -0.74785243 | -2.401663 | 0.9059582 | 1 |
| Au-Yeung SSY （2009） | SOC_LICT | -0.6278636 | -1.496805 | 0.2410776 | 1 |
| Au-Yeung SSY （2009） | SOC_MICT | -0.70442145 | -1.424796 | 0.0159528 | 1 |
| Wu C (2025) | SOC_HIIT | -0.56695317 | -2.352613 | 1.218707 | 1 |
| Wu C (2025) | SOC_LICT | -0.44694053 | -1.375818 | 0.4819369 | 1 |
| Wu C (2025) | SOC_MICT | -0.71068054 | -1.506631 | 0.0852698 | 1 |
| Yeh TT （2022） | SOC_HIIT | -0.47112851 | -1.753354 | 0.8110974 | 1 |
| Yeh TT （2022） | SOC_LICT | -0.35103577 | -0.8732257 | 0.1711541 | 1 |
| Yeh TT （2022） | SOC_MICT | -1.0766895 | -1.782424 | -0.3709549 | 1 |
| Munari D (2018) | SOC_HIIT | -0.38662495 | -1.029259 | 0.2560088 | 1 |
| Munari D (2018) | SOC_LICT | -0.70077494 | -1.385338 | -0.0162115 | 1 |
| Kim J （2017） | SOC_HIIT | -0.27515682 | -1.733083 | 1.182769 | 1 |
| Kim J （2017） | SOC_LICT | -0.15503696 | -0.8408598 | 0.5307859 | 1 |
| Kim J （2017） | SOC_MICT | -0.6953328 | -1.337044 | -0.0536214 | 1 |

Supplementary Table 13.Meta-Regression Analysis of the VO_2peak_ Using Age as a Covariate

|  |  | **Coefficient** | **Std. err.** | **z** | **P>z** | **lower confidence interval** | **upper confidence interval** |
| --- | --- | --- | --- | --- | --- | --- | --- |
|  |  |  |  |  |  |  |  |
| HICT VS HIIT |  |  |  |  |  |  |  |
|  | cov1 | -0.6941309 | 0.405502 | -1.71 | 0.087 | -1.4889 | 0.1006384 |
|  | _cons | 47.27828 | 25.47642 | 1.86 | 0.063 | -2.65458 | 97.21114 |
|  |  |  |  |  |  |  |  |
| HICT VS LICT |  |  |  |  |  |  |  |
|  | cov1 | -0.1795013 | 0.381197 | -0.47 | 0.638 | -0.9266336 | 0.567631 |
|  | _cons | 10.52309 | 22.94667 | 0.46 | 0.647 | -34.45155 | 55.49772 |
|  |  |  |  |  |  |  |  |
| HICT VS MICT |  |  |  |  |  |  |  |
|  | cov1 | -0.3631691 | 0.3127127 | -1.16 | 0.245 | -0.9760748 | 0.2497365 |
|  | _cons | 24.24788 | 19.42372 | 1.25 | 0.212 | -13.82191 | 62.31767 |
|  |  |  |  |  |  |  |  |
| HICT VS Non-training |  |  |  |  |  |  |  |
|  | _cons | -2.587251 | 1.94966 | -1.33 | 0.184 | -6.408514 | 1.234013 |
|  |  |  |  |  |  |  |  |
| HICT VS SOC |  |  |  |  |  |  |  |
|  | cov1 | -0.2256684 | 0.2734506 | -0.83 | 0.409 | -0.7616218 | 0.3102849 |
|  | _cons | 12.22332 | 17.10757 | 0.71 | 0.475 | -21.30689 | 45.75354 |

Supplementary Table 14.Meta-Regression Analysis of the VO_2peak_ Using Total intervention duration(min) as a Covariate

|  |  | **Coefficient** | **Std. err.** | **z** | **P>z** | **lower confidence interval** | **upper confidence interval** |
| --- | --- | --- | --- | --- | --- | --- | --- |
|  |  |  |  |  |  |  |  |
| HICT VS HIIT |  |  |  |  |  |  |  |
|  | cov2 | 0.0007847 | 0.0024006 | 0.33 | 0.744 | -0.0039205 | 0.0054899 |
|  | _cons | 1.679563 | 4.003208 | 0.42 | 0.675 | -6.166579 | 9.525706 |
|  |  |  |  |  |  |  |  |
| HICT VS LICT |  |  |  |  |  |  |  |
|  | cov2 | -0.0000531 | 0.0014404 | -0.04 | 0.971 | -0.0028762 | 0.00277 |
|  | _cons | -0.6295539 | 2.796862 | -0.23 | 0.822 | -6.111302 | 4.852195 |
|  |  |  |  |  |  |  |  |
| HICT VS MICT |  |  |  |  |  |  |  |
|  | cov2 | 0.0009342 | 0.0012833 | 0.73 | 0.467 | -0.0015811 | 0.0034494 |
|  | _cons | -0.6904585 | 2.94904 | -0.23 | 0.815 | -6.470471 | 5.089554 |
|  |  |  |  |  |  |  |  |
| HICT VS Non-training |  |  |  |  |  |  |  |
|  | _cons | -2.679574 | 2.109311 | -1.27 | 0.204 | -6.813748 | 1.454599 |
|  |  |  |  |  |  |  |  |
| HICT VS SOC |  |  |  |  |  |  |  |
|  | cov2 | 0.0002867 | 0.0008564 | 0.33 | 0.738 | -0.0013919 | 0.0019652 |
|  | _cons | -2.409584 | 1.680197 | -1.43 | 0.152 | -5.702709 | 0.8835417 |

Supplementary Table 15.Meta-Regression Analysis of the VO_2peak_ Using Baseline gait speed as a Covariate

|  |  | **Coefficient** | **Std. err.** | **z** | **P>z** | **lower confidence interval** | **upper confidence interval** |
| --- | --- | --- | --- | --- | --- | --- | --- |
|  |  |  |  |  |  |  |  |
| HICT VS HIIT |  |  |  |  |  |  |  |
|  | cov3 | -16.66109 | 15.03544 | -1.11 | 0.268 | -46.13 | 12.80782 |
|  | _cons | 16.28171 | 10.55267 | 1.54 | 0.123 | -4.401149 | 36.96457 |
|  |  |  |  |  |  |  |  |
| HICT VS LICT |  |  |  |  |  |  |  |
|  | cov3 | -11.2944 | 8.097689 | -1.39 | 0.163 | -27.16557 | 4.576783 |
|  | _cons | 5.173086 | 5.27523 | 0.98 | 0.327 | -5.166175 | 15.51235 |
|  |  |  |  |  |  |  |  |
| HICT VS MICT |  |  |  |  |  |  |  |
|  | cov3 | -27.38459 | 12.42132 | -2.2 | 0.271 | -51.72993 | -3.039248 |
|  | _cons | 21.21005 | 9.12038 | 2.33 | 0.145 | 3.33443 | 39.08566 |
|  |  |  |  |  |  |  |  |
| HICT VS Non-training |  |  |  |  |  |  |  |
|  | _cons | -2.045593 | 1.606008 | -1.27 | 0.203 | -5.193312 | 1.102125 |
|  |  |  |  |  |  |  |  |
| HICT VS SOC |  |  |  |  |  |  |  |
|  | cov3 | -27.06666 | 11.93826 | -2.27 | 0.202 | -50.46522 | -3.668108 |
|  | _cons | 17.82351 | 8.614048 | 2.07 | 0.167 | 0.9402899 | 34.70674 |

Supplementary Table 16.Meta-Regression Analysis of the VO_2peak_ Using Baseline VO_2peak_ as a Covariate

|  |  | Coefficient | Std. err. | z | P>z | lower confidence interval | upper confidence interval |
| --- | --- | --- | --- | --- | --- | --- | --- |
|  |  |  |  |  |  |  |  |
| HICT VS HIIT |  |  |  |  |  |  |  |
|  | cov4 | -0.0955892 | 0.6757789 | -0.14 | 0.888 | -1.420091 | 1.228913 |
|  | _cons | 4.406652 | 11.13117 | 0.4 | 0.692 | -17.41005 | 26.22335 |
|  |  |  |  |  |  |  |  |
| HICT VS LICT |  |  |  |  |  |  |  |
|  | cov4 | -0.0483374 | 0.7236755 | -0.07 | 0.947 | -1.466715 | 1.370041 |
|  | _cons | -0.1171234 | 10.90207 | -0.01 | 0.991 | -21.48478 | 21.25054 |
|  |  |  |  |  |  |  |  |
| HICT VS MICT |  |  |  |  |  |  |  |
|  | cov4 | -0.2866237 | 0.5795497 | -0.49 | 0.621 | -1.42252 | 0.8492729 |
|  | _cons | 5.770173 | 9.158488 | 0.63 | 0.529 | -12.18013 | 23.72048 |
|  |  |  |  |  |  |  |  |
| HICT VS Non-training |  |  |  |  |  |  |  |
|  | _cons | -2.195712 | 2.016348 | -1.09 | 0.276 | -6.147681 | 1.756258 |
|  |  |  |  |  |  |  |  |
| HICT VS SOC |  |  |  |  |  |  |  |
|  | cov4 | -0.5701317 | 0.4868772 | -1.17 | 0.242 | -1.524393 | 0.38413 |
|  | _cons | 6.81027 | 7.939011 | 0.86 | 0.391 | -8.749906 | 22.37044 |

Supplementary Table 17.Meta-Regression Analysis of the 6MWT Using Age as a Covariate

|  |  | Coefficient | Std. err. | z | P>z | lower confidence interval | upper confidence interval |
| --- | --- | --- | --- | --- | --- | --- | --- |
|  |  |  |  |  |  |  |  |
| HICT VS HIIT |  |  |  |  |  |  |  |
|  | cov1 | 14.94009 | 9.763801 | 1.53 | 0.126 | -4.196604 | 34.07679 |
|  | _cons | -886.0662 | 602.1144 | -1.47 | 0.141 | -2066.189 | 294.0563 |
|  |  |  |  |  |  |  |  |
| HICT VS LICT |  |  |  |  |  |  |  |
|  | cov1 | 0.0400919 | 5.075142 | 0.01 | 0.994 | -9.907004 | 9.987188 |
|  | _cons | -46.66588 | 310.4959 | -0.15 | 0.881 | -655.2266 | 561.8949 |
|  |  |  |  |  |  |  |  |
| HICT VS MICT |  |  |  |  |  |  |  |
|  | cov1 | 6.834201 | 5.331492 | 1.28 | 0.2 | -3.615331 | 17.28373 |
|  | _cons | -414.9088 | 336.2307 | -1.23 | 0.217 | -1073.909 | 244.0912 |
|  |  |  |  |  |  |  |  |
| HICT VS Non-training |  |  |  |  |  |  |  |
|  | cov1 | -1.288162 | 12.25825 | -0.11 | 0.916 | -25.31389 | 22.73756 |
|  | _cons | 45.02847 | 762.6796 | 0.06 | 0.953 | -1449.796 | 1539.853 |
|  |  |  |  |  |  |  |  |
| HICT VS SOC |  |  |  |  |  |  |  |
|  | cov1 | 4.756996 | 5.257015 | 0.9 | 0.366 | -5.546564 | 15.06056 |
|  | _cons | -328.9572 | 332.4059 | -0.99 | 0.322 | -980.4609 | 322.5465 |

Supplementary Table 18.Meta-Regression Analysis of the 6MWT Using Total intervention duration(min) as a Covariate

|  |  | Coefficient | Std. err. | z | P>z | lower confidence interval | upper confidence interval |
| --- | --- | --- | --- | --- | --- | --- | --- |
|  |  |  |  |  |  |  |  |
| HICT VS HIIT |  |  |  |  |  |  |  |
|  | cov2 | 0.0341913 | 0.034499 | 0.99 | 0.322 | -0.0334255 | 0.101808 |
|  | _cons | -22.94243 | 52.01751 | -0.44 | 0.659 | -124.8949 | 79.01001 |
|  |  |  |  |  |  |  |  |
| HICT VS LICT |  |  |  |  |  |  |  |
|  | cov2 | -0.0310479 | 0.0251232 | -1.24 | 0.217 | -0.0802886 | 0.0181927 |
|  | _cons | 19.67031 | 38.47276 | 0.51 | 0.609 | -55.73492 | 95.07554 |
|  |  |  |  |  |  |  |  |
| HICT VS MICT |  |  |  |  |  |  |  |
|  | cov2 | 0.0093666 | 0.0207121 | 0.45 | 0.651 | -0.0312283 | 0.0499615 |
|  | _cons | -17.13426 | 35.98501 | -0.48 | 0.634 | -87.66358 | 53.39507 |
|  |  |  |  |  |  |  |  |
| HICT VS Non-training |  |  |  |  |  |  |  |
|  | cov2 | 0.0263274 | 0.0482088 | 0.55 | 0.585 | -0.0681601 | 0.1208148 |
|  | _cons | -55.5081 | 62.75217 | -0.88 | 0.376 | -178.5001 | 67.48389 |
|  |  |  |  |  |  |  |  |
| HICT VS SOC |  |  |  |  |  |  |  |
|  | cov2 | 0.0015652 | 0.0162291 | 0.1 | 0.923 | -0.0302432 | 0.0333736 |
|  | _cons | -30.37102 | 32.47853 | -0.94 | 0.35 | -94.02777 | 33.28573 |

Supplementary Table 19.Meta-Regression Analysis of the 6MWT Using Baseline gait speed as a Covariate

|  |  | **Coefficient** | **Std. err.** | **z** | **P>z** | **lower confidence interval** | **upper confidence interval** |
| --- | --- | --- | --- | --- | --- | --- | --- |
|  |  |  |  |  |  |  |  |
| HICT VS HIIT |  |  |  |  |  |  |  |
|  | cov3 | -258.5913 | 211.14 | -1.22 | 0.221 | -672.4181 | 155.2355 |
|  | _cons | 230.8085 | 139.9406 | 1.65 | 0.099 | -43.47 | 505.087 |
|  |  |  |  |  |  |  |  |
| HICT VS LICT |  |  |  |  |  |  |  |
|  | cov3 | 59.23451 | 87.46672 | 0.68 | 0.498 | -112.1971 | 230.6661 |
|  | _cons | -80.29096 | 51.42372 | -1.56 | 0.118 | -181.0796 | 20.49768 |
|  |  |  |  |  |  |  |  |
| HICT VS MICT |  |  |  |  |  |  |  |
|  | cov3 | 303.4495 | 109.0301 | 2.78 | 0.062 | 89.75446 | 517.1446 |
|  | _cons | -164.9069 | 64.64958 | -2.55 | 0.232 | -291.6177 | -38.19605 |
|  |  |  |  |  |  |  |  |
| HICT VS Non-training |  |  |  |  |  |  |  |
|  | cov3 | 204.4234 | 170.1392 | 1.2 | 0.23 | -129.0433 | 537.8901 |
|  | _cons | -134.555 | 77.76236 | -1.73 | 0.084 | -286.9664 | 17.85645 |
|  |  |  |  |  |  |  |  |
| HICT VS SOC |  |  |  |  |  |  |  |
|  | cov3 | 254.9132 | 141.5115 | 1.8 | 0.072 | -22.44423 | 532.2707 |
|  | _cons | -191.7652 | 97.87673 | -1.96 | 0.051 | -383.6 | 0.0696945 |

Supplementary Table 20.Meta-Regression Analysis of the 6MWT Using Baseline VO_2peak_ as a Covariate

|  |  | **Coefficient** | **Std. err.** | **z** | **P>z** | **lower confidence interval** | **upper confidence interval** |
| --- | --- | --- | --- | --- | --- | --- | --- |
|  |  |  |  |  |  |  |  |
| HICT VS HIIT |  |  |  |  |  |  |  |
|  | cov4 | -5.768435 | 17.8181 | -0.32 | 0.746 | -40.69127 | 29.1544 |
|  | _cons | 126.1568 | 295.2537 | 0.43 | 0.669 | -452.5297 | 704.8434 |
|  |  |  |  |  |  |  |  |
| HICT VS LICT |  |  |  |  |  |  |  |
|  | cov4 | -1.882788 | 14.85605 | -0.13 | 0.899 | -31.00012 | 27.23454 |
|  | _cons | -25.71921 | 235.6464 | -0.11 | 0.913 | -487.5776 | 436.1392 |
|  |  |  |  |  |  |  |  |
| HICT VS MICT |  |  |  |  |  |  |  |
|  | cov4 | 5.105802 | 15.96295 | 0.32 | 0.749 | -26.181 | 36.3926 |
|  | _cons | -67.93443 | 263.0546 | -0.26 | 0.796 | -583.5119 | 447.643 |
|  |  |  |  |  |  |  |  |
| HICT VS Non-training |  |  |  |  |  |  |  |
|  | _cons | -8.589809 | 81.57008 | -0.11 | 0.916 | -168.4642 | 151.2846 |
|  |  |  |  |  |  |  |  |
| HICT VS SOC |  |  |  |  |  |  |  |
|  | cov4 | -10.85545 | 17.15905 | -0.63 | 0.527 | -44.48656 | 22.77567 |
|  | _cons | 155.1089 | 303.9363 | 0.51 | 0.61 | -440.5953 | 750.8131 |

Supplementary Table 21.Meta-Regression Analysis of the 10MWT Using Age as a Covariate

|  |  | **Coefficient** | **Std. err.** | **z** | **P>z** | **lower confidence interval** | **upper confidence interval** |
| --- | --- | --- | --- | --- | --- | --- | --- |
|  |  |  |  |  |  |  |  |
| HICT VS HIIT |  |  |  |  |  |  |  |
|  | cov1 | -0.062393 | 0.1616409 | -0.39 | 0.699 | -0.3792034 | 0.2544174 |
|  | _cons | 4.707851 | 10.38191 | 0.45 | 0.65 | -15.64032 | 25.05602 |
|  |  |  |  |  |  |  |  |
| HICT VS LICT |  |  |  |  |  |  |  |
|  | cov1 | 0.0428709 | 0.1065801 | 0.4 | 0.688 | -0.1660223 | 0.2517642 |
|  | _cons | -2.8323 | 7.102053 | -0.4 | 0.69 | -16.75207 | 11.08747 |
|  |  |  |  |  |  |  |  |
| HICT VS MICT |  |  |  |  |  |  |  |
|  | cov1 | 0.0454017 | 0.1236156 | 0.37 | 0.713 | -0.1968803 | 0.2876837 |
|  | _cons | -2.731427 | 8.149008 | -0.34 | 0.737 | -18.70319 | 13.24033 |
|  |  |  |  |  |  |  |  |
| HICT VS Non-training |  |  |  |  |  |  |  |
|  | _cons | -0.2171589 | 0.7423399 | -0.29 | 0.77 | -1.672118 | 1.237801 |
|  |  |  |  |  |  |  |  |
| HICT VS SOC |  |  |  |  |  |  |  |
|  | cov1 | 0.0418391 | 0.1028918 | 0.41 | 0.684 | -0.1598251 | 0.2435034 |
|  | _cons | -3.149309 | 6.92954 | -0.45 | 0.649 | -16.73096 | 10.43234 |

Supplementary Table 22.Meta-Regression Analysis of the 10MWT Using Total intervention duration(min) as a Covariate

|  |  | **Coefficient** | **Std. err.** | **z** | **P>z** | **lower confidence interval** | **upper confidence interval** |
| --- | --- | --- | --- | --- | --- | --- | --- |
|  |  |  |  |  |  |  |  |
| HICT VS HIIT |  |  |  |  |  |  |  |
|  | cov2 | 0.0000782 | 0.0005795 | 0.13 | 0.893 | -0.0010576 | 0.0012139 |
|  | _cons | 1.073742 | 0.6497835 | 1.65 | 0.098 | -0.1998098 | 2.347295 |
|  |  |  |  |  |  |  |  |
| HICT VS LICT |  |  |  |  |  |  |  |
|  | cov2 | 0.0002382 | 0.0004628 | 0.51 | 0.607 | -0.0006688 | 0.0011452 |
|  | _cons | -0.1683267 | 0.4635343 | -0.36 | 0.717 | -1.076837 | 0.7401837 |
|  |  |  |  |  |  |  |  |
| HICT VS MICT |  |  |  |  |  |  |  |
|  | cov2 | 0.0002382 | 0.000465 | 0.51 | 0.608 | -0.0006731 | 0.0011496 |
|  | _cons | 0.0922598 | 0.4439999 | 0.21 | 0.835 | -0.7779639 | 0.9624836 |
|  |  |  |  |  |  |  |  |
| HICT VS Non-training |  |  |  |  |  |  |  |
|  | _cons | 0.088905 | 0.4514185 | 0.2 | 0.844 | -0.795859 | 0.9736691 |
|  |  |  |  |  |  |  |  |
| HICT VS SOC |  |  |  |  |  |  |  |
|  | cov2 | 0.0003106 | 0.0005102 | 0.61 | 0.543 | -0.0006893 | 0.0013106 |
|  | _cons | -0.6946842 | 0.614326 | -1.13 | 0.258 | -1.898741 | 0.5093726 |

Supplementary Table 23.Meta-Regression Analysis of the 10MWT Using Baseline gait speed as a Covariate

|  |  | **Coefficient** | **Std. err.** | **z** | **P>z** | **lower confidence interval** | **upper confidence interval** |
| --- | --- | --- | --- | --- | --- | --- | --- |
|  |  |  |  |  |  |  |  |
| HICT VS HIIT |  |  |  |  |  |  |  |
|  | cov3 | 0.8910861 | 1.55216 | 0.57 | 0.566 | -2.151092 | 3.933264 |
|  | _cons | 0.4710063 | 1.037713 | 0.45 | 0.65 | -1.562874 | 2.504886 |
|  |  |  |  |  |  |  |  |
| HICT VS LICT |  |  |  |  |  |  |  |
|  | cov3 | 0.5842794 | 1.125585 | 0.52 | 0.604 | -1.621827 | 2.790386 |
|  | _cons | -0.4539025 | 0.9378138 | -0.48 | 0.628 | -2.291984 | 1.384179 |
|  |  |  |  |  |  |  |  |
| HICT VS MICT |  |  |  |  |  |  |  |
|  | cov3 | 0.115766 | 0.9215818 | 0.13 | 0.9 | -1.690501 | 1.922033 |
|  | _cons | 0.1735688 | 0.6672455 | 0.26 | 0.795 | -1.134208 | 1.481346 |
|  |  |  |  |  |  |  |  |
| HICT VS Non-training |  |  |  |  |  |  |  |
|  | _cons | -0.2786013 | 0.7117653 | -0.39 | 0.695 | -1.673636 | 1.116433 |
|  |  |  |  |  |  |  |  |
| HICT VS SOC |  |  |  |  |  |  |  |
|  | cov3 | 0.4581817 | 0.9855042 | 0.46 | 0.642 | -1.473371 | 2.389734 |
|  | _cons | -0.7368065 | 0.8170555 | -0.9 | 0.367 | -2.338206 | 0.8645928 |

Supplementary Table 24.Meta-Regression Analysis of the 10MWT Using Baseline VO_2peak_ as a Covariate

|  |  | **Coefficient** | **Std. err.** | **z** | **P>z** | **lower confidence interval** | **upper confidence interval** |
| --- | --- | --- | --- | --- | --- | --- | --- |
|  |  |  |  |  |  |  |  |
| HICT VS HIIT |  |  |  |  |  |  |  |
|  | cov4 | -0.0537034 | 1.756733 | -0.03 | 0.976 | -3.496837 | 3.38943 |
|  | _cons | 2.118403 | 30.21457 | 0.07 | 0.944 | -57.10106 | 61.33786 |
|  |  |  |  |  |  |  |  |
| HICT VS LICT |  |  |  |  |  |  |  |
|  | cov4 | 0.0337653 | 1.753879 | 0.02 | 0.985 | -3.403775 | 3.471306 |
|  | _cons | -0.279345 | 30.16141 | -0.01 | 0.993 | -59.39463 | 58.83594 |
|  |  |  |  |  |  |  |  |
| HICT VS MICT |  |  |  |  |  |  |  |
|  | cov4 | -0.1280579 | 1.759236 | -0.07 | 0.942 | -3.576098 | 3.319982 |
|  | _cons | 2.42784 | 30.25707 | 0.08 | 0.936 | -56.87493 | 61.73061 |
|  |  |  |  |  |  |  |  |
| HICT VS Non-training |  |  |  |  |  |  |  |
|  | _cons | -0.269193 | 29.63574 | -0.01 | 0.993 | -58.35417 | 57.81579 |
|  |  |  |  |  |  |  |  |
| HICT VS SOC |  |  |  |  |  |  |  |
|  | cov4 | 0.1000334 | 0.6646897 | 0.15 | 0.88 | -1.202735 | 1.402801 |
|  | _cons | -2.268966 | 13.42549 | -0.17 | 0.866 | -28.58245 | 24.04452 |

Supplementary Table 25 .Meta-Regression Analysis of the BBS Using Age as a Covariate

|  |  | **Coefficient** | **Std. err.** | **z** | **P>z** | **lower confidence interval** | **upper confidence interval** |
| --- | --- | --- | --- | --- | --- | --- | --- |
|  |  |  |  |  |  |  |  |
| HICT VS HIIT |  |  |  |  |  |  |  |
|  | _cons | 3.920586 | 9.807187 | 0.4 | 0.689 | -15.30115 | 23.14232 |
|  |  |  |  |  |  |  |  |
| HICT VS LICT |  |  |  |  |  |  |  |
|  | cov1 | 0.0121384 | 1.335442 | 0.01 | 0.993 | -2.605279 | 2.629556 |
|  | _cons | 0.155124 | 90.58044 | 0 | 0.999 | -177.3793 | 177.6895 |
|  |  |  |  |  |  |  |  |
| HICT VS MICT |  |  |  |  |  |  |  |
|  | cov1 | -0.2635158 | 1.305941 | -0.2 | 0.84 | -2.823114 | 2.296082 |
|  | _cons | 16.79325 | 88.73411 | 0.19 | 0.85 | -157.1224 | 190.7089 |
|  |  |  |  |  |  |  |  |
| HICT VS Non-training |  |  |  |  |  |  |  |
|  | _cons | 0.4055261 | 10.34083 | 0.04 | 0.969 | -19.86212 | 20.67317 |
|  |  |  |  |  |  |  |  |
| HICT VS SOC |  |  |  |  |  |  |  |
|  | cov1 | -0.1534284 | 1.29644 | -0.12 | 0.906 | -2.694404 | 2.387547 |
|  | _cons | 7.879148 | 88.25806 | 0.09 | 0.929 | -165.1035 | 180.8618 |

Supplementary Table 26.Meta-Regression Analysis of the BBS Using Total intervention duration(min) as a Covariate

|  |  | **Coefficient** | **Std. err.** | **z** | **P>z** | **lower confidence interval** | **upper confidence interval** |
| --- | --- | --- | --- | --- | --- | --- | --- |
|  |  |  |  |  |  |  |  |
| HICT VS HIIT |  |  |  |  |  |  |  |
|  | _cons | 5.365691 | 4.801657 | 1.12 | 0.264 | -4.045384 | 14.77677 |
|  |  |  |  |  |  |  |  |
| HICT VS LICT |  |  |  |  |  |  |  |
|  | cov2 | -0.0022224 | 0.0045471 | -0.49 | 0.625 | -0.0111344 | 0.0066897 |
|  | _cons | 2.051417 | 3.866041 | 0.53 | 0.596 | -5.525884 | 9.628717 |
|  |  |  |  |  |  |  |  |
| HICT VS MICT |  |  |  |  |  |  |  |
|  | cov2 | -0.0031321 | 0.0046152 | -0.68 | 0.497 | -0.0121776 | 0.0059135 |
|  | _cons | 4.260947 | 4.573905 | 0.93 | 0.352 | -4.703742 | 13.22564 |
|  |  |  |  |  |  |  |  |
| HICT VS Non-training |  |  |  |  |  |  |  |
|  | _cons | -0.8387219 | 5.16956 | -0.16 | 0.871 | -10.97087 | 9.293429 |
|  |  |  |  |  |  |  |  |
| HICT VS SOC |  |  |  |  |  |  |  |
|  | cov2 | -0.0027497 | 0.0045385 | -0.61 | 0.545 | -0.011645 | 0.0061455 |
|  | _cons | 1.279797 | 4.262322 | 0.3 | 0.764 | -7.074201 | 9.633794 |

Supplementary Table 27.Meta-Regression Analysis of the BBS Using Baseline gait speed as a Covariate

|  |  | **Coefficient** | **Std. err.** | **z** | **P>z** | **lower confidence interval** | **upper confidence interval** |
| --- | --- | --- | --- | --- | --- | --- | --- |
|  |  |  |  |  |  |  |  |
| HICT VS HIIT |  |  |  |  |  |  |  |
|  | _cons | 4.359541 | 26.26603 | 0.17 | 0.868 | -47.12092 | 55.84001 |
|  |  |  |  |  |  |  |  |
| HICT VS LICT |  |  |  |  |  |  |  |
|  | cov3 | 1.121955 | 542.1244 | 0 | 0.998 | -1061.422 | 1063.666 |
|  | _cons | -0.0995157 | 531.268 | 0 | 1 | -1041.366 | 1041.167 |
|  |  |  |  |  |  |  |  |
| HICT VS MICT |  |  |  |  |  |  |  |
|  | cov3 | -0.7476555 | 57.78465 | -0.01 | 0.99 | -114.0035 | 112.5082 |
|  | _cons | 1.336127 | 51.71786 | 0.03 | 0.979 | -100.029 | 102.7013 |
|  |  |  |  |  |  |  |  |
| HICT VS Non-training |  |  |  |  |  |  |  |
|  | _cons | -0.2529293 | 368.637 | 0 | 0.999 | -722.7681 | 722.2623 |
|  |  |  |  |  |  |  |  |
| HICT VS SOC |  |  |  |  |  |  |  |
|  | cov3 | -2.231517 | 57.73686 | -0.04 | 0.969 | -115.3937 | 110.9307 |
|  | _cons | -0.6899863 | 51.71692 | -0.01 | 0.989 | -102.0533 | 100.6733 |

Supplementary Table 28.Meta-Regression Analysis of the BBS Using Baseline VO_2peak_ as a Covariate

|  |  | **Coefficient** | **Std. err.** | **z** | **P>z** | **lower confidence interval** | **upper confidence interval** |
| --- | --- | --- | --- | --- | --- | --- | --- |
|  |  |  |  |  |  |  |  |
| HICT VS HIIT |  |  |  |  |  |  |  |
|  | _cons | 6.672065 | 57.87033 | 0.12 | 0.908 | -106.7517 | 120.0958 |
|  |  |  |  |  |  |  |  |
| HICT VS LICT |  |  |  |  |  |  |  |
|  | cov4 | -1.021666 | 14.4769 | -0.07 | 0.944 | -29.39587 | 27.35254 |
|  | _cons | 18.15407 | 293.6455 | 0.06 | 0.951 | -557.3805 | 593.6887 |
|  |  |  |  |  |  |  |  |
| HICT VS MICT |  |  |  |  |  |  |  |
|  | cov4 | -0.4051312 | 14.48929 | -0.03 | 0.978 | -28.80362 | 27.99336 |
|  | _cons | 9.915875 | 293.7652 | 0.03 | 0.973 | -565.8532 | 585.685 |
|  |  |  |  |  |  |  |  |
| HICT VS SOC |  |  |  |  |  |  |  |
|  | cov4 | -0.9791968 | 14.49527 | -0.07 | 0.946 | -29.38941 | 27.43101 |
|  | _cons | 17.24879 | 294.0224 | 0.06 | 0.953 | -559.0245 | 593.5221 |

Supplementary Table 29 .Meta-Regression Analysis of the TUG Using Age as a Covariate

|  |  | **Coefficient** | **Std. err.** | **z** | **P>z** | **lower confidence interval** | **upper confidence interval** |
| --- | --- | --- | --- | --- | --- | --- | --- |
|  |  |  |  |  |  |  |  |
| HIIT VS LICT |  |  |  |  |  |  |  |
|  | cov1 | 0.5636087 | 9.944705 | 0.06 | 0.955 | -18.92765 | 20.05487 |
|  | _cons | -34.1302 | 611.1753 | -0.06 | 0.955 | -1232.012 | 1163.751 |
|  |  |  |  |  |  |  |  |
| HIIT VS MICT |  |  |  |  |  |  |  |
|  | cov1 | 0.0182016 | 9.971536 | 0 | 0.999 | -19.52565 | 19.56205 |
|  | _cons | -3.518577 | 612.3059 | -0.01 | 0.995 | -1203.616 | 1196.579 |
|  |  |  |  |  |  |  |  |
| HIIT VS SOC |  |  |  |  |  |  |  |
|  | cov1 | -0.1851073 | 9.950971 | -0.02 | 0.985 | -19.68865 | 19.31844 |
|  | _cons | 12.72763 | 611.2529 | 0.02 | 0.983 | -1185.306 | 1210.761 |

Supplementary Table 30.Meta-Regression Analysis of the TUG Using Total intervention duration(min) as a Covariate

|  |  | **Coefficient** | **Std. err.** | **z** | **P>z** | **lower confidence interval** | **upper confidence interval** |
| --- | --- | --- | --- | --- | --- | --- | --- |
|  |  |  |  |  |  |  |  |
| HIIT VS LICT |  |  |  |  |  |  |  |
|  | cov2 | 0.0021408 | 0.0475384 | 0.05 | 0.964 | -0.0910327 | 0.0953143 |
|  | _cons | -3.709625 | 93.84141 | -0.04 | 0.968 | -187.6354 | 180.2162 |
|  |  |  |  |  |  |  |  |
| HIIT VS MICT |  |  |  |  |  |  |  |
|  | cov2 | 0.0036722 | 0.0484529 | 0.08 | 0.94 | -0.0912938 | 0.0986381 |
|  | _cons | -4.251804 | 93.92837 | -0.05 | 0.964 | -188.348 | 179.8444 |
|  |  |  |  |  |  |  |  |
| HIIT VS SOC |  |  |  |  |  |  |  |
|  | cov2 | 0.0010241 | 0.0475508 | 0.02 | 0.983 | -0.0921738 | 0.0942219 |
|  | _cons | 2.02958 | 93.71469 | 0.02 | 0.983 | -181.6478 | 185.707 |

Supplementary Table 31.Meta-Regression Analysis of the TUG Using Baseline gait speed as a Covariate

|  |  | **Coefficient** | **Std. err.** | **z** | **P>z** | **lower confidence interval** | **upper confidence interval** |
| --- | --- | --- | --- | --- | --- | --- | --- |
|  |  |  |  |  |  |  |  |
| HIIT VS LICT |  |  |  |  |  |  |  |
|  | cov3 | 20.80913 | 919.0784 | 0.02 | 0.982 | -1780.551 | 1822.17 |
|  | _cons | -17.57395 | 799.6278 | -0.02 | 0.982 | -1584.816 | 1549.668 |
|  |  |  |  |  |  |  |  |
| HIIT VS MICT |  |  |  |  |  |  |  |
|  | cov3 | -4.564861 | 948.8771 | 0 | 0.996 | -1864.33 | 1855.2 |
|  | _cons | -0.1610887 | 793.3361 | 0 | 1 | -1555.071 | 1554.749 |
|  |  |  |  |  |  |  |  |
| HIIT VS SOC |  |  |  |  |  |  |  |
|  | cov3 | 1.463568 | 948.855 | 0 | 0.999 | -1858.258 | 1861.185 |
|  | _cons | 2.021848 | 793.3131 | 0 | 0.998 | -1552.843 | 1556.887 |

Supplementary Table 32 Baseline Ventilatory Threshold, Fugl-Meyerleg motor score, and National Institutes of Health Stroke Scale (NIHSS)

| **First author**  **(year)** | **Ventilatory Threshold (VT)** | | **Fugl-Meyerleg motor score** | | **NIHSS** | |
| --- | --- | --- | --- | --- | --- | --- |
|  | **Intervention** | **Control** | **Intervention** | **Control** | **Intervention** | **Control** |
| Boyne P  （2016） | 10.3±3.6 | 13.9±2.2 | 24.2±4.8 | 23.2±7.3 | Na | Na |
| Boyne P (2023) | 12.1±3.9 | 11.6±3.9 | 23.8±5.1 | 22.8±5.1 | Na | Na |
| Hong J  (2013) | Na | Na | Na | Na | Na | Na |
| Lapointe T  (2023) | Na | Na | Na | Na | Na | Na |
| Munari D  (2018) | Na | Na | Na | Na | Na | Na |
| Tang A  （2014） | Na | Na | Na | Na | Na | Na |
| Globas C  （2012） | Na | Na | Na | Na | 4.4±2.6 | 4.4±2.6 |
| Gordon CD  （2013） | Na | Na | Na | Na | Na | Na |
| Hornby TG  （2019） | Na | Na | 23±4.6 | 23±4.6 | Na | Na |
| Ivey FM  （2010） | Na | Na | Na | Na | Na | Na |
| Ivey FM  （2007） | Na | Na | Na | Na | Na | Na |
| Lee MJ  （2008） | Na | Na | Na | Na | Na | Na |

| Macko RF  （2005） | Na | Na | Na | Na | Na | Na |
| --- | --- | --- | --- | --- | --- | --- |
| Quaney BM  (2009) | Na | Na | Na | Na | Na | Na |
| Serra MC  (2019） | Na | Na | Na | Na | Na | Na |
| Moore SA  (2015) | Na | Na | Na | Na | 3±3.2 | 3±3.2 |
| Kim J  (2017) | Na | Na | Na | Na | Na | Na |
| Liu-Ambrose T  （2015） | Na | Na | Na | Na | Na | Na |
| Yeh TT  （2019） | Na | Na | Na | Na | 4.73±1.35 | 4.07±0.8 |
| Yeh TT  （2022） | Na | Na | Na | Na | 4.5±3.3 | 4.6±3 |
| Doğan Duran Ü  （2022） | Na | Na | Na | Na | Na | Na |
| Mustafaoğlu R  （2018） | Na | Na | Na | Na | Na | Na |
| Kim SJ  （2015） | Na | Na | Na | Na | Na | Na |
| Au-Yeung SSY  （2009） | Na | Na | Na | Na | Na | Na |
| Mberti NLA  （2017） | Na | Na | Na | Na | Na | Na |
| Rimmer JH  （2009） | Na | Na | Na | Na | Na | Na |

| Thompson ED  （2023） | 9.63±0.3 | 9.37±0.26 | Na | Na | Na | Na |
| --- | --- | --- | --- | --- | --- | --- |
| Moncion K  （2024） | Na | Na | Na | Na | 2±3.3 | 2±2.7 |
| Linder SM  （2024） | 10.5±3.4 | 10.5±3.4 | Na | Na | Na | Na |
| Boyne P  （2025） | 12.1±3.9 | 11.6±3.9 | 23.8±5.1 | 22.8±5.1 | Na | Na |
| Do J  (2025) | Na | Na | 23.72 ± 5.79 | 20.29 ± 6.53 | Na | Na |
| Wu C  (2025) | Na | Na | 28.20±3.09 | 27.10±2.77 | Na | Na |
| Palmcrantz S  (2021) | Na | Na | 15.44±5.76 | 17.29±4.83 | 7±1.2 | 8±3.1 |


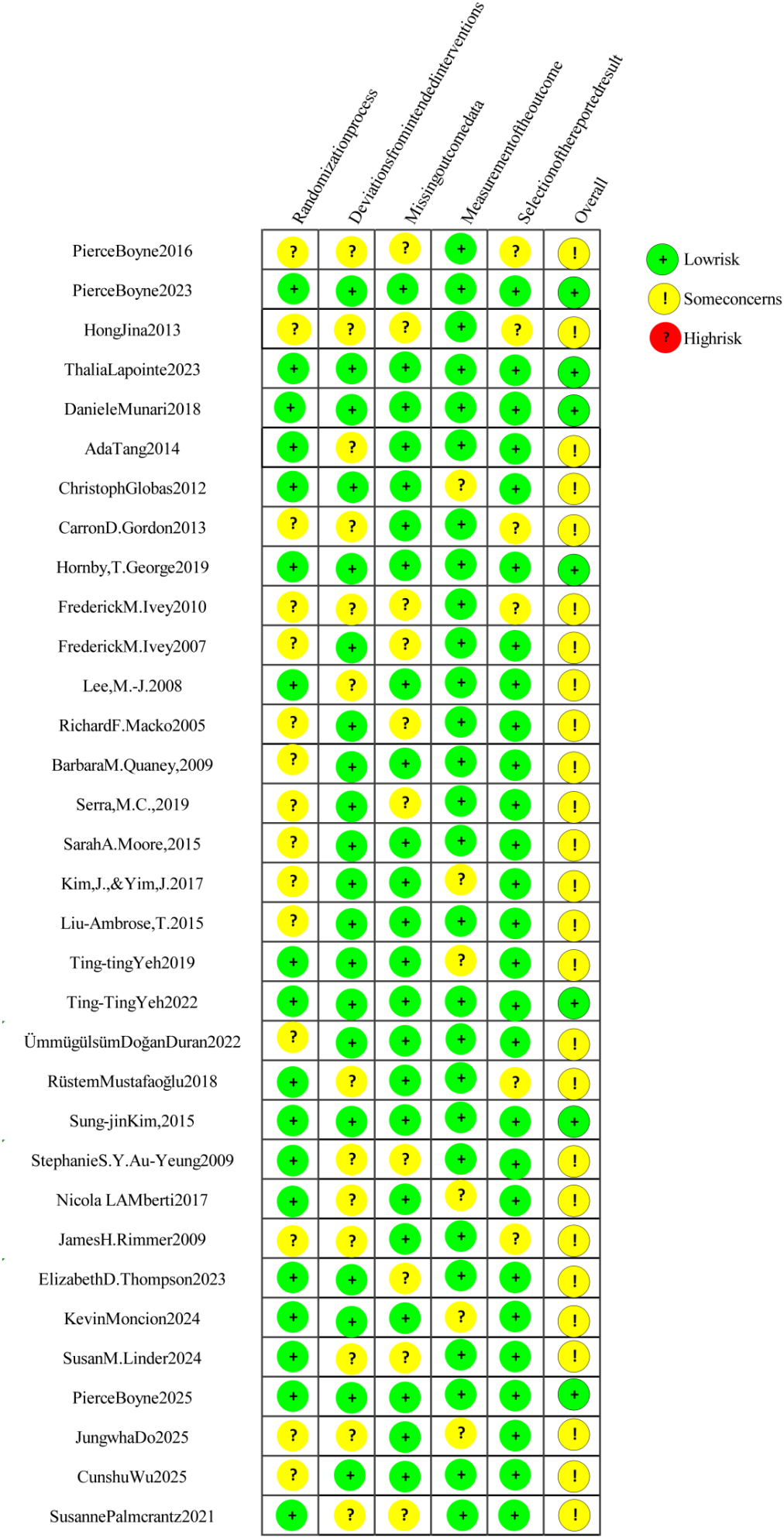


Supplementary Figure 1.Risk of bias assessment of included RCTs


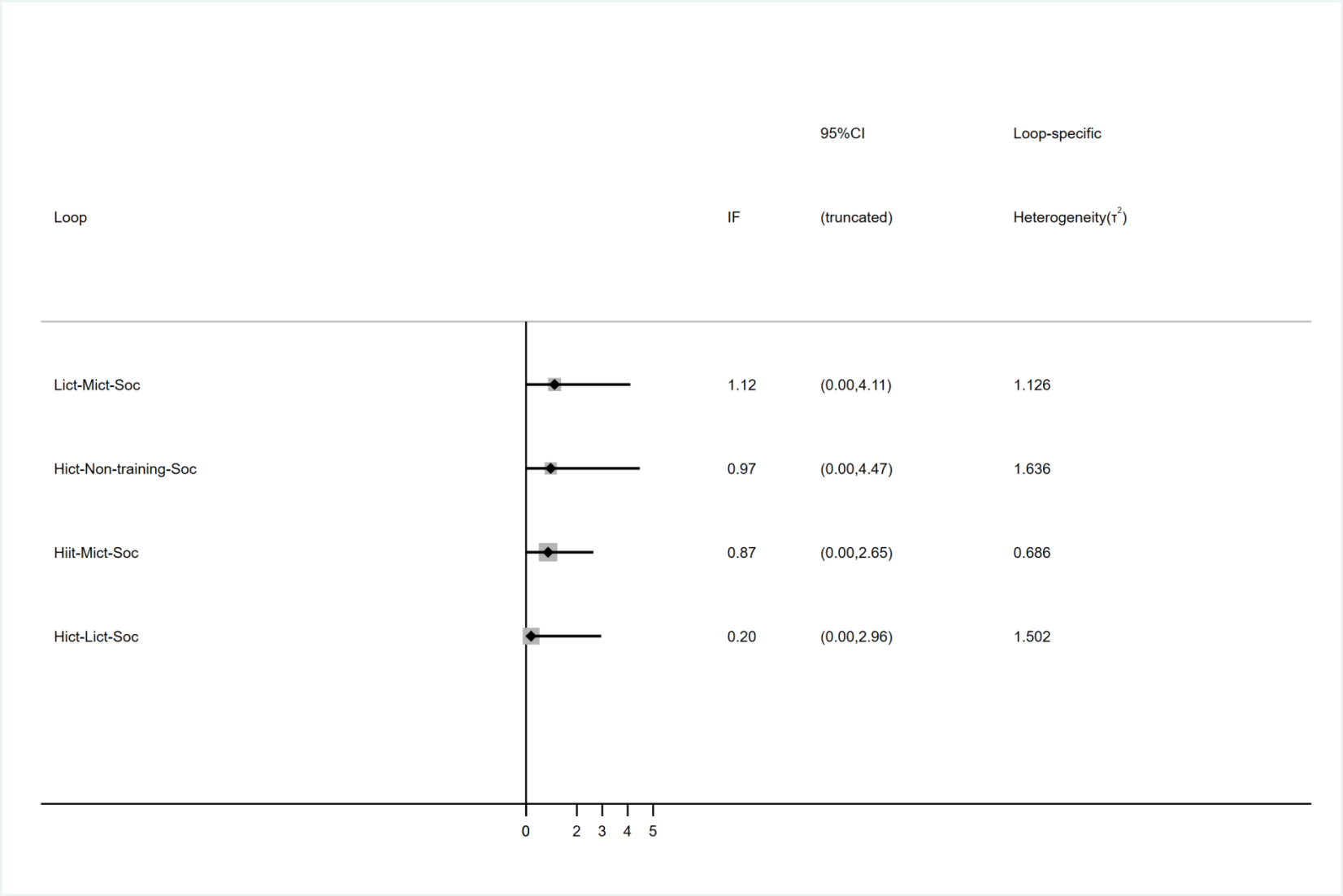


Supplementary Figure 2.Loop-specific inconsistency test for the VO_2peak_


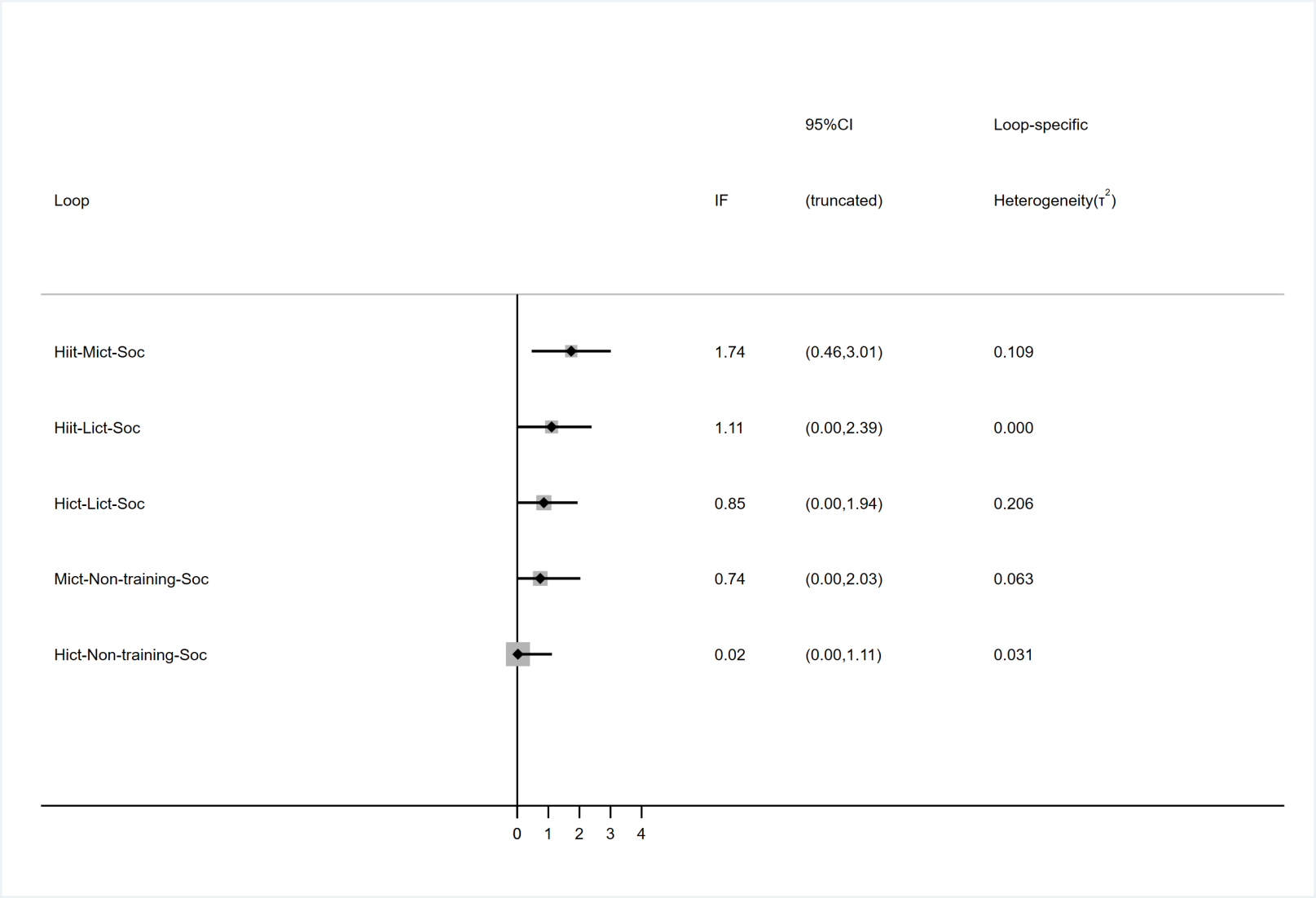


Supplementary Figure 3.Loop-specific inconsistency test for the 6MWT


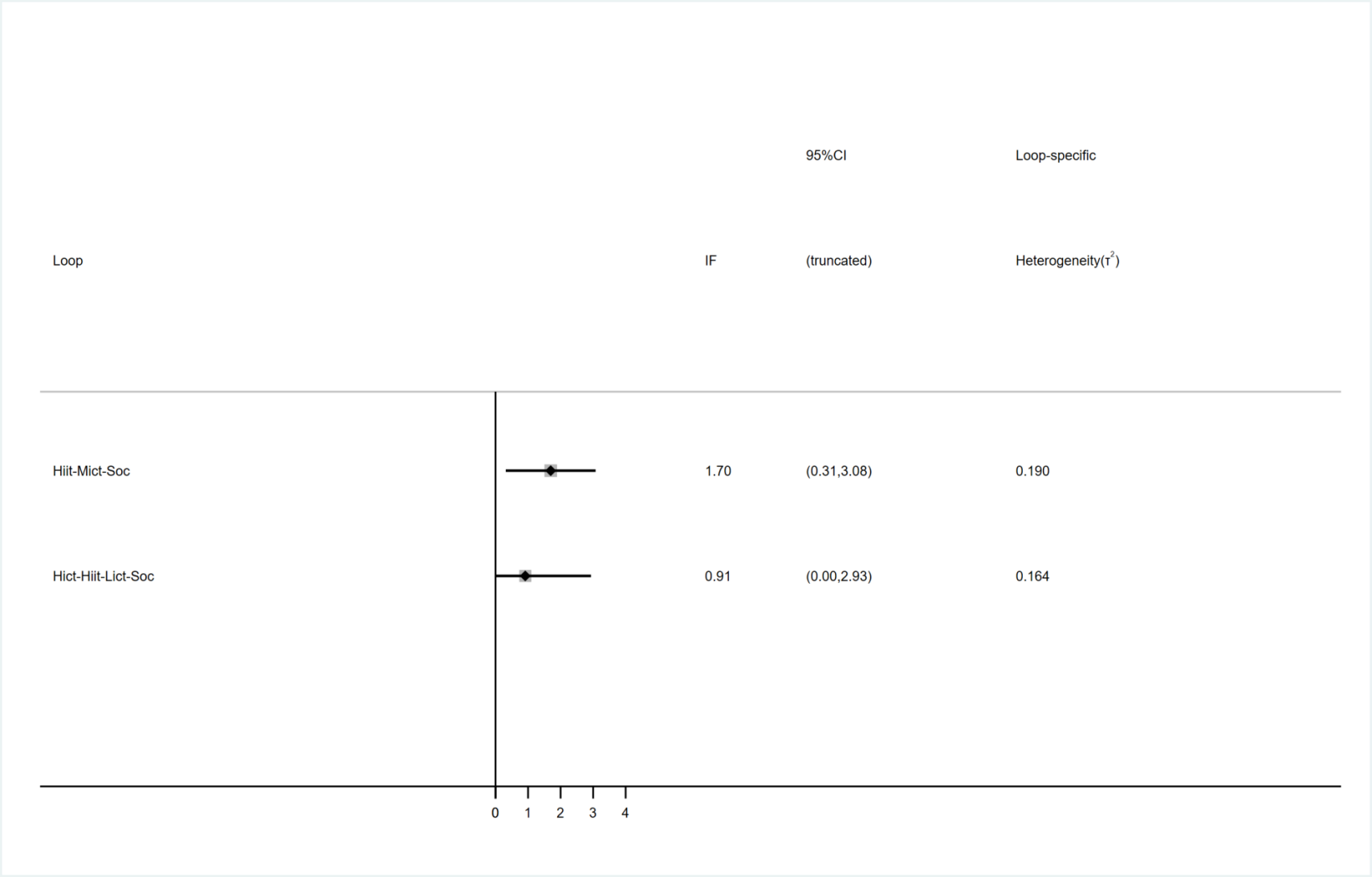


Supplementary Figure 4.Loop-specific inconsistency test for the 10MWT


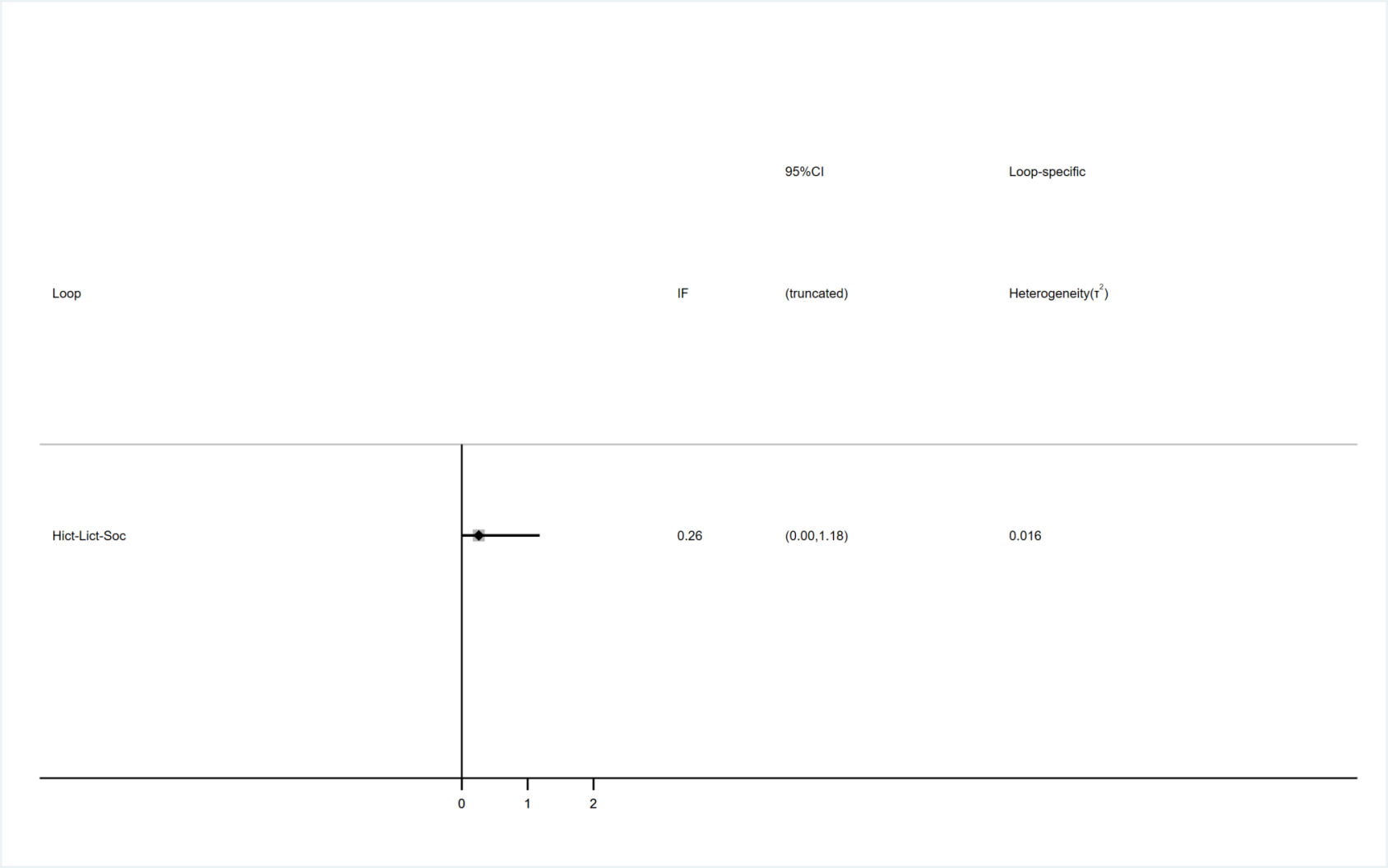


Supplementary Figure 5.Loop-specific inconsistency test for the BBS


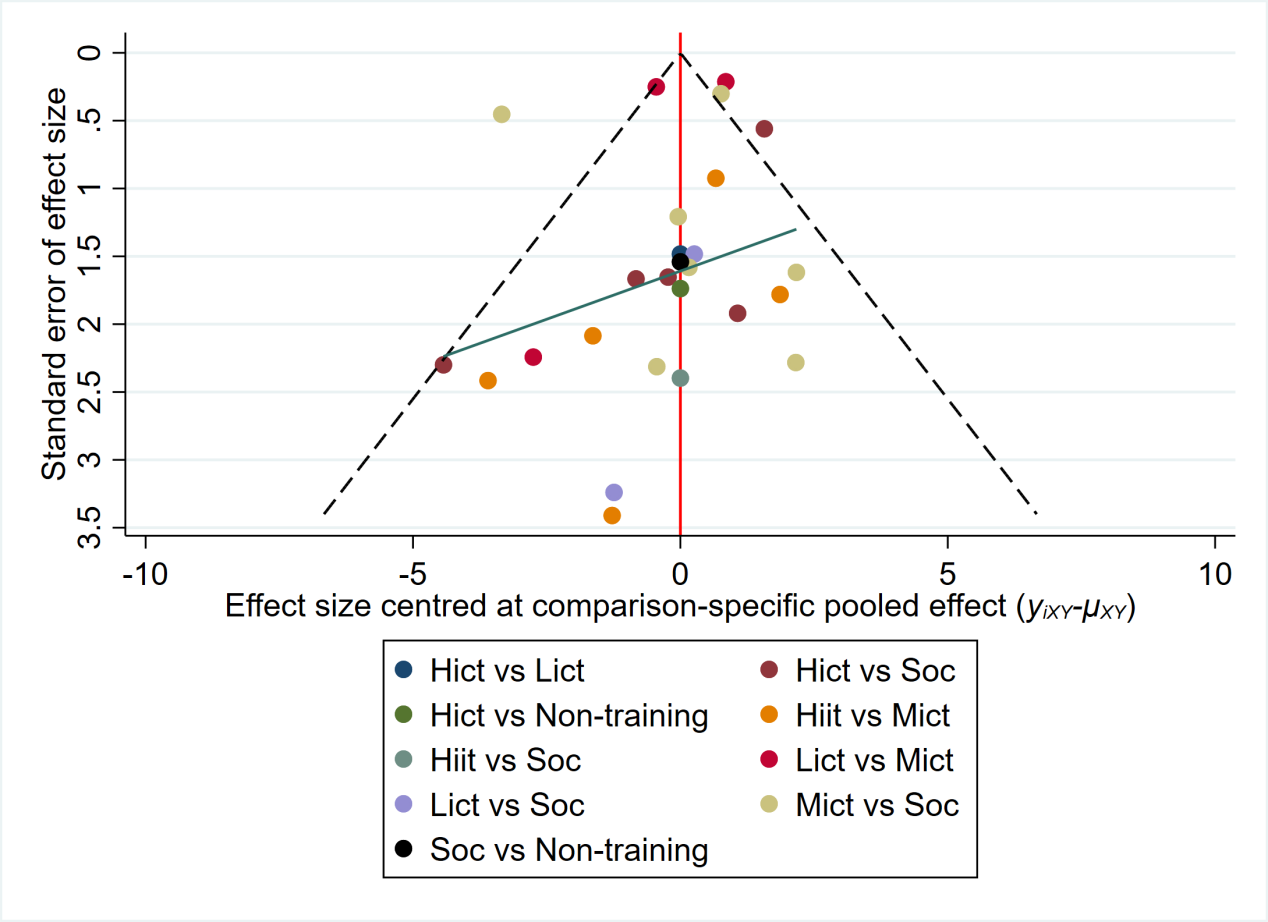


Supplementary Figure 6.Comparison-adjusted funnel plot for the VO_2peak_ outcome


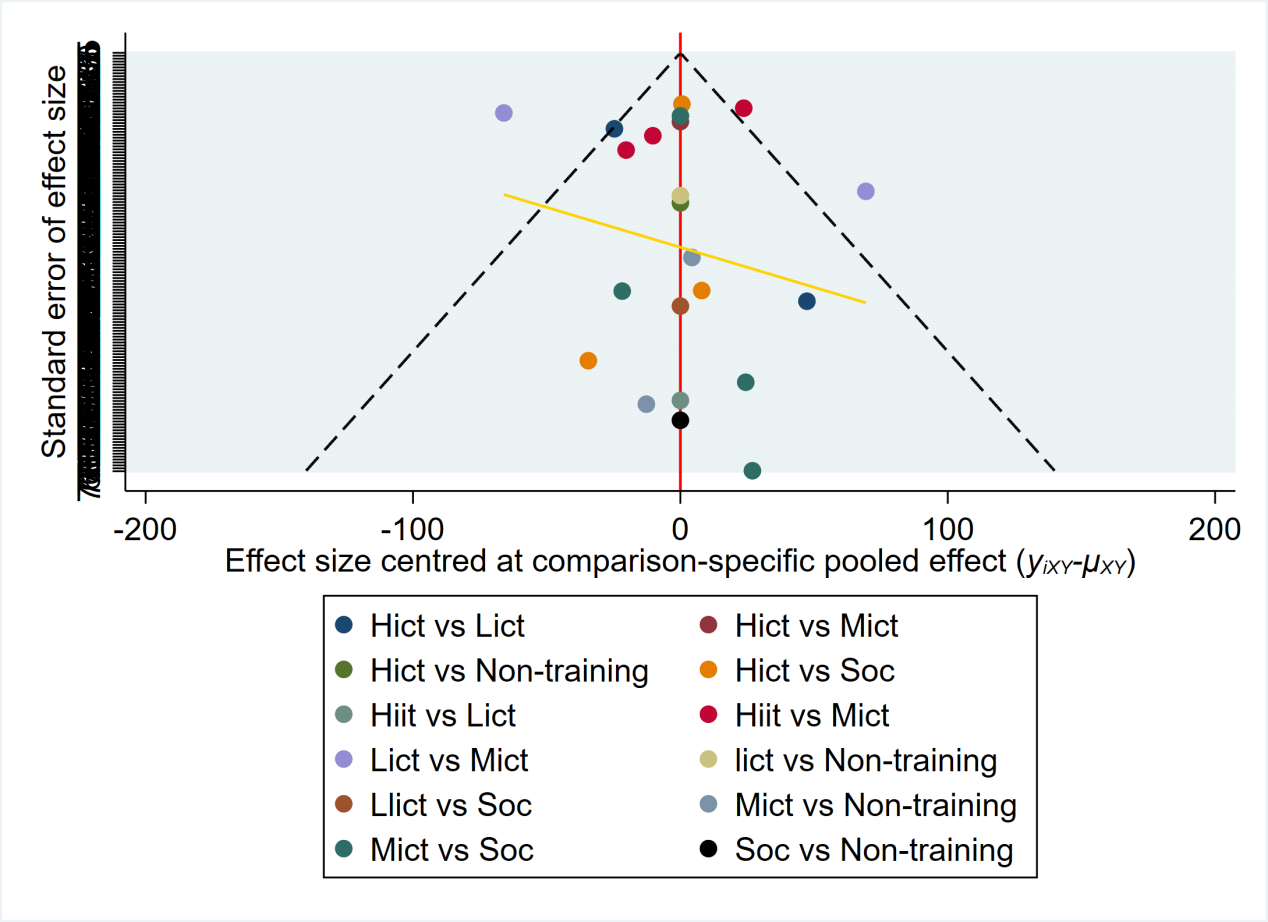


Supplementary Figure 7.Comparison-adjusted funnel plot for the 6MWT outcome


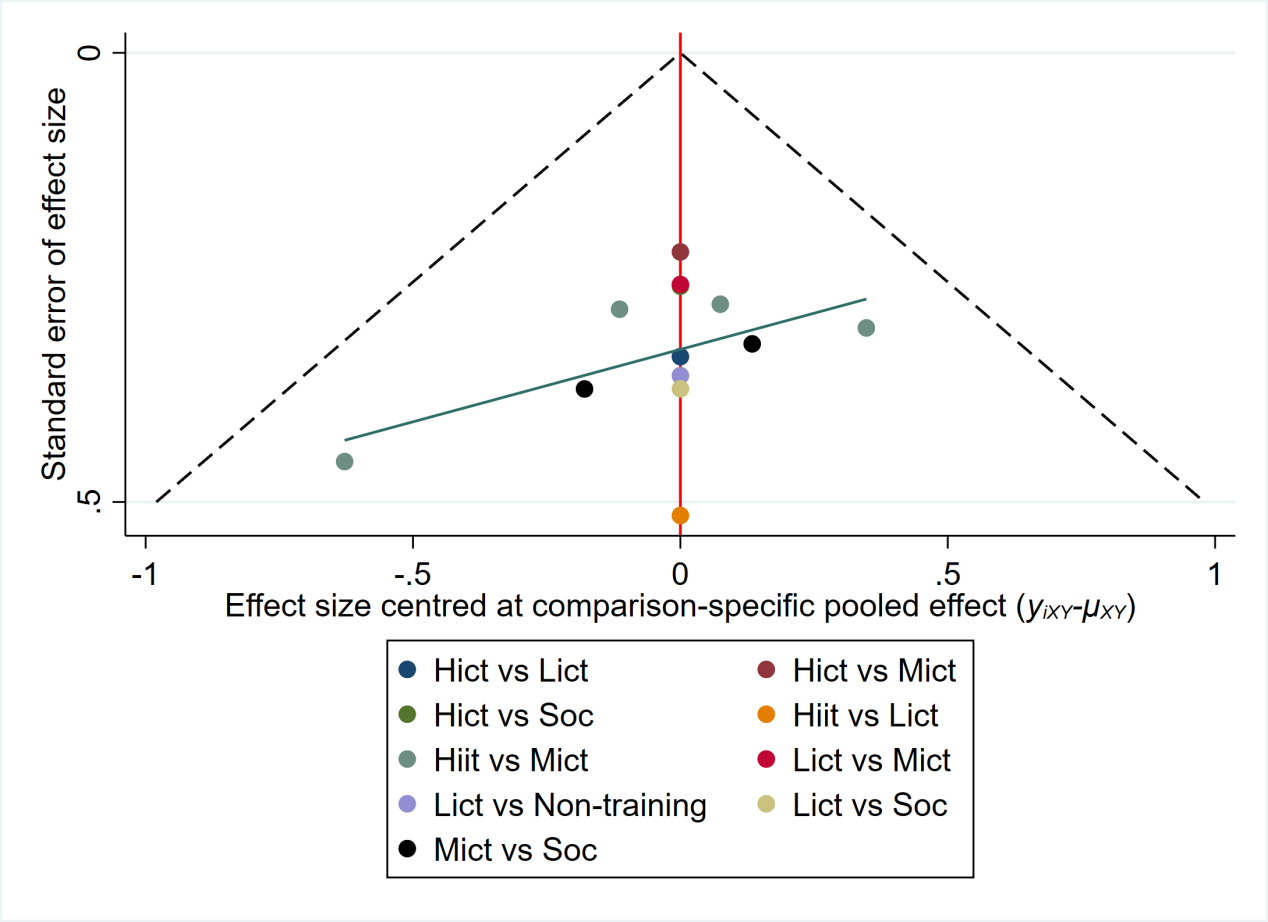


Supplementary Figure 8.Comparison-adjusted funnel plot for the 10MWT outcome


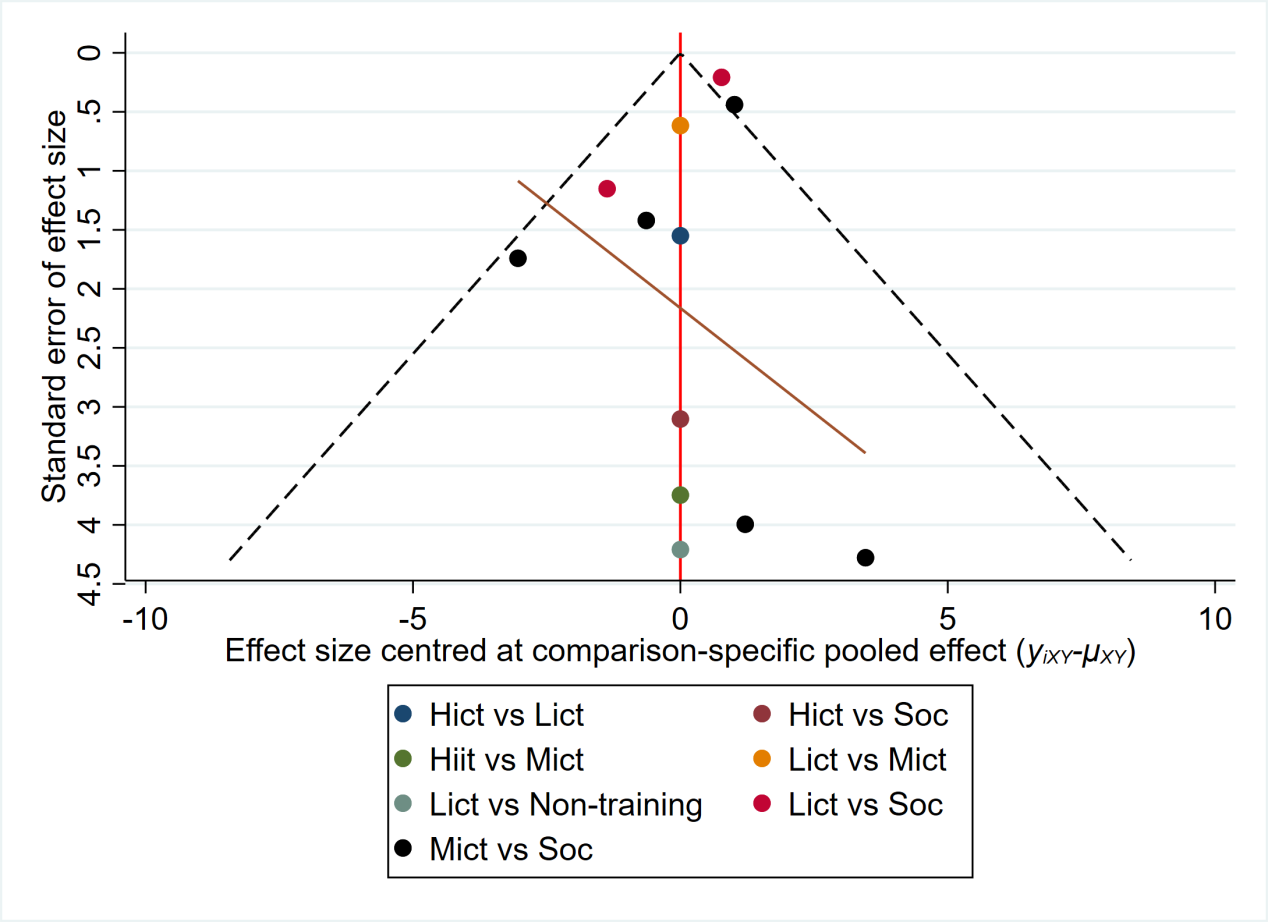


Supplementary Figure 9.Comparison-adjusted funnel plot for the BBS outcome


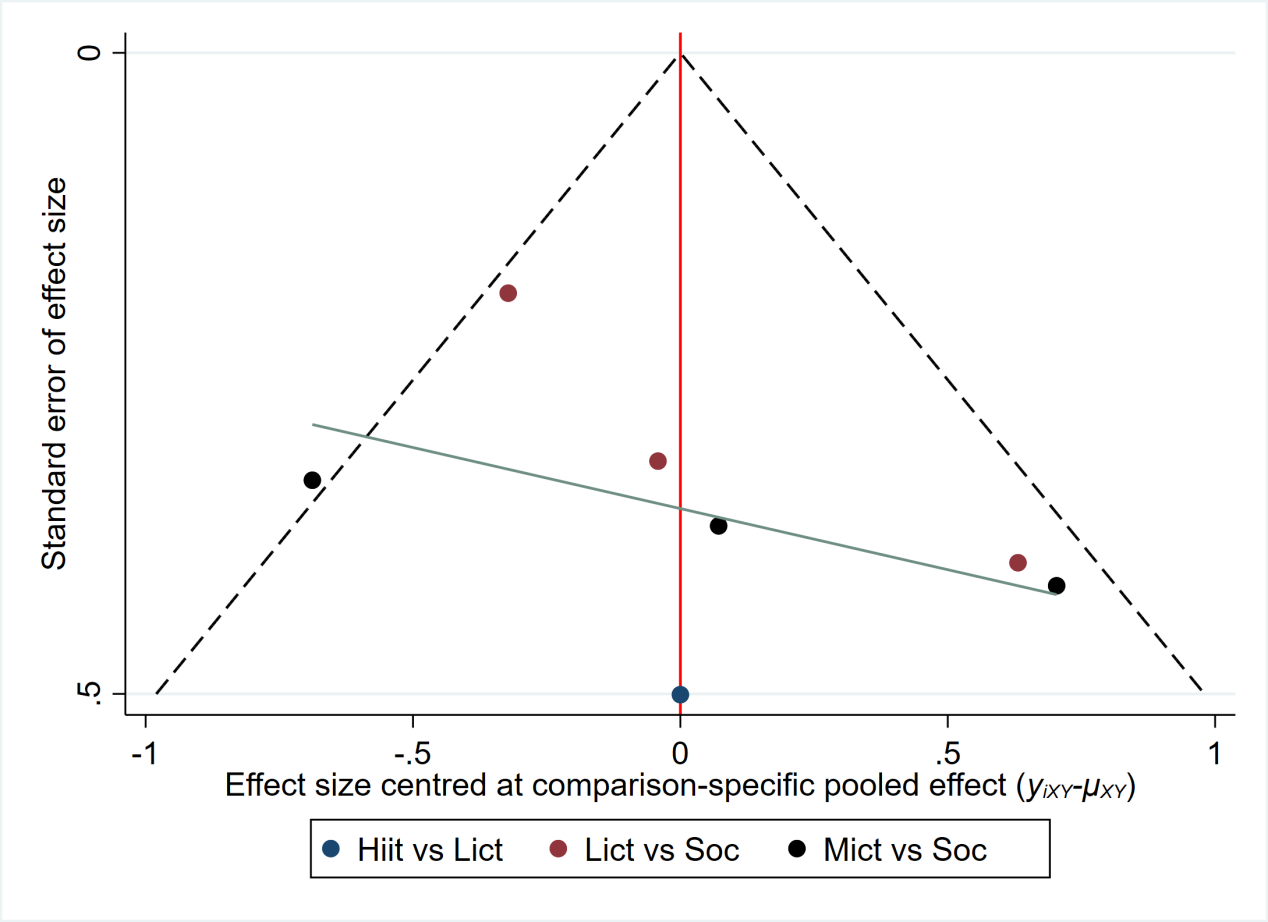


Supplementary Figure 10.Comparison-adjusted funnel plot for the TUG outcome


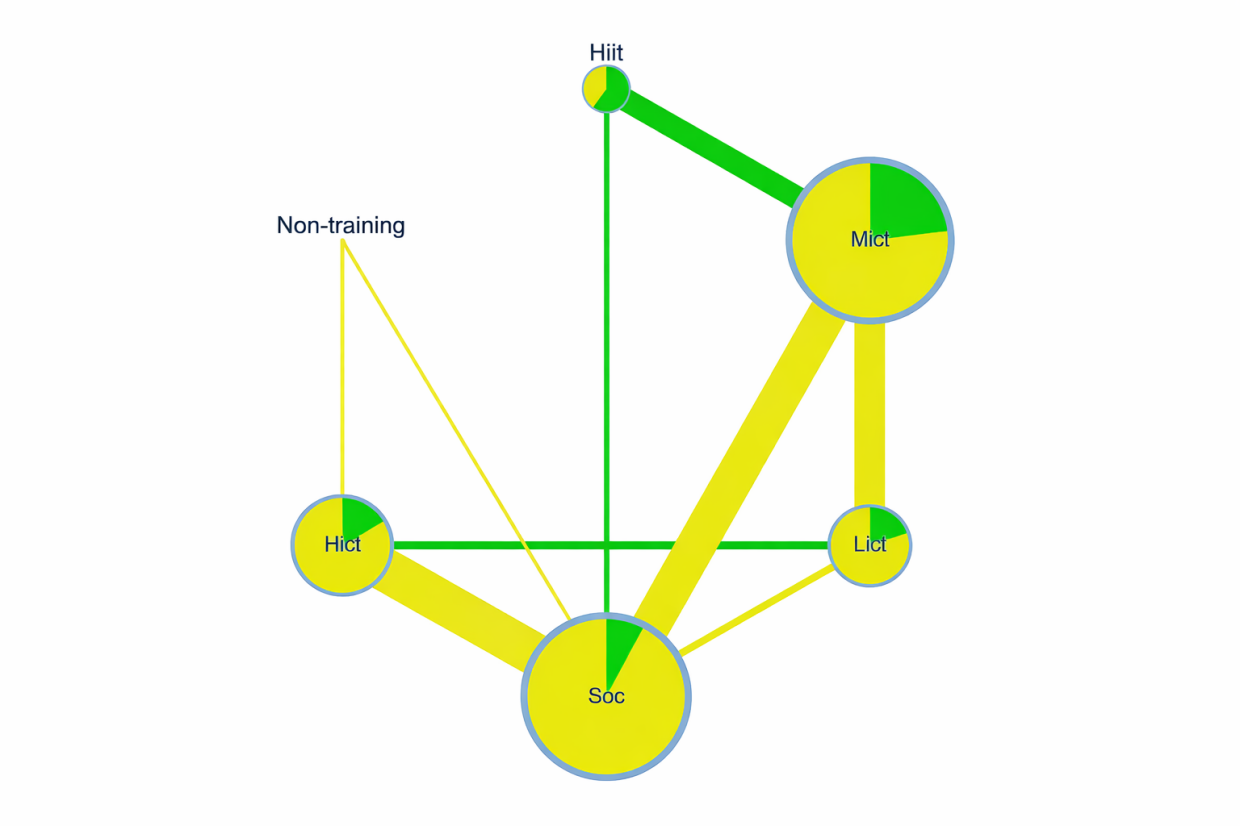


Supplementary Figure 11.CINeMA contribution plot of within-study bias (RoB 2.0) for the VO_2peak_ outcome


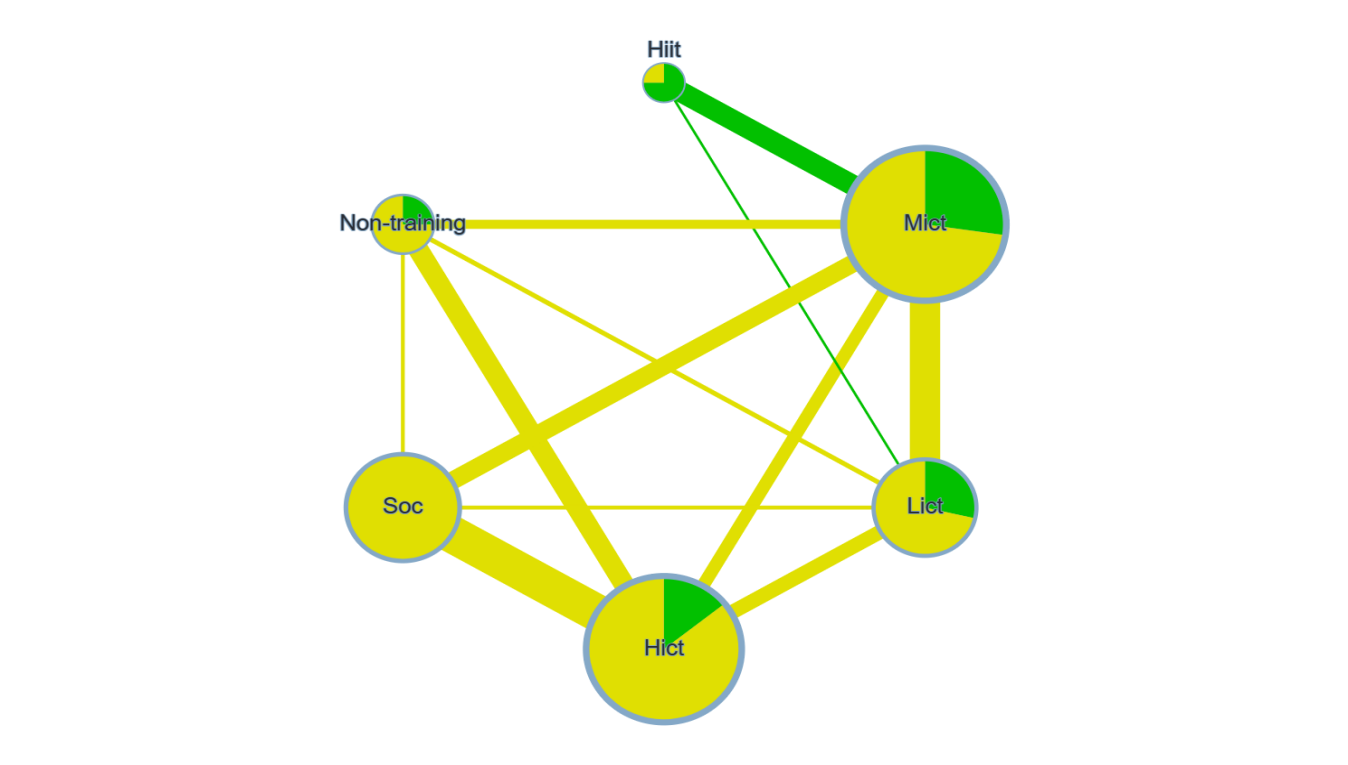


Supplementary Figure 12.CINeMA contribution plot of within-study bias (RoB 2.0) for the 6MWT outcome


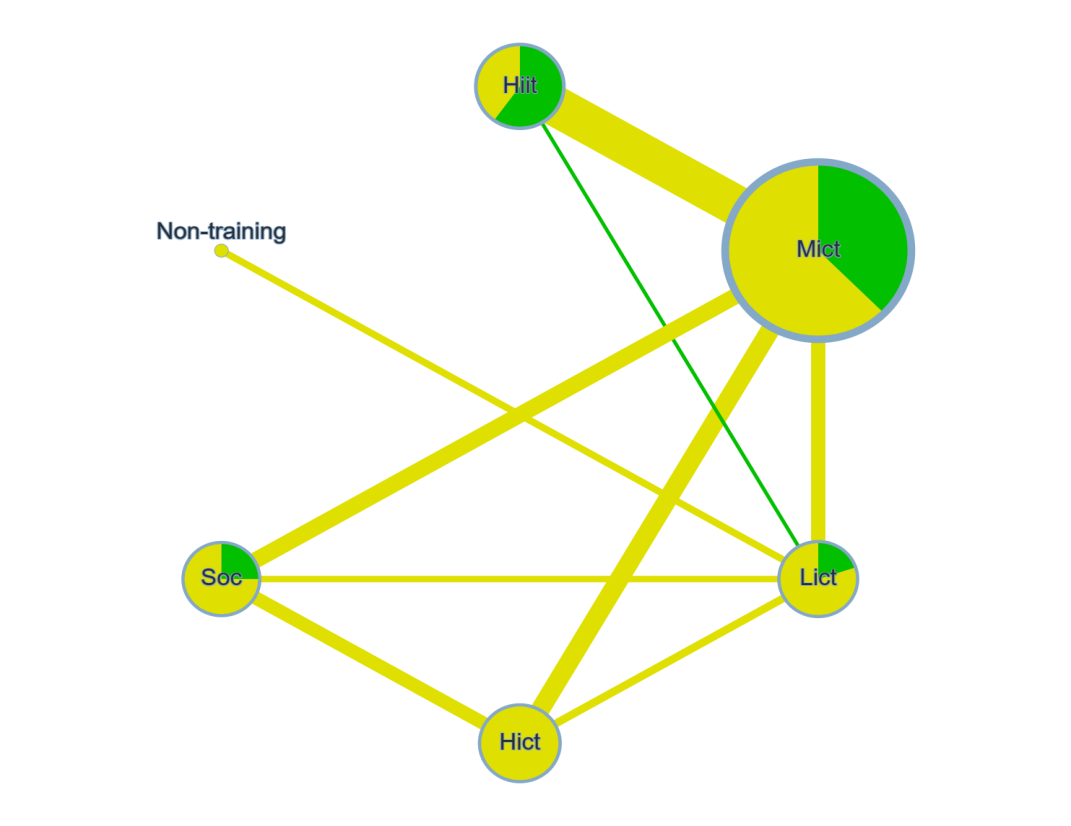


Supplementary Figure 13.CINeMA contribution plot of within-study bias (RoB 2.0) for the 10MWT outcome


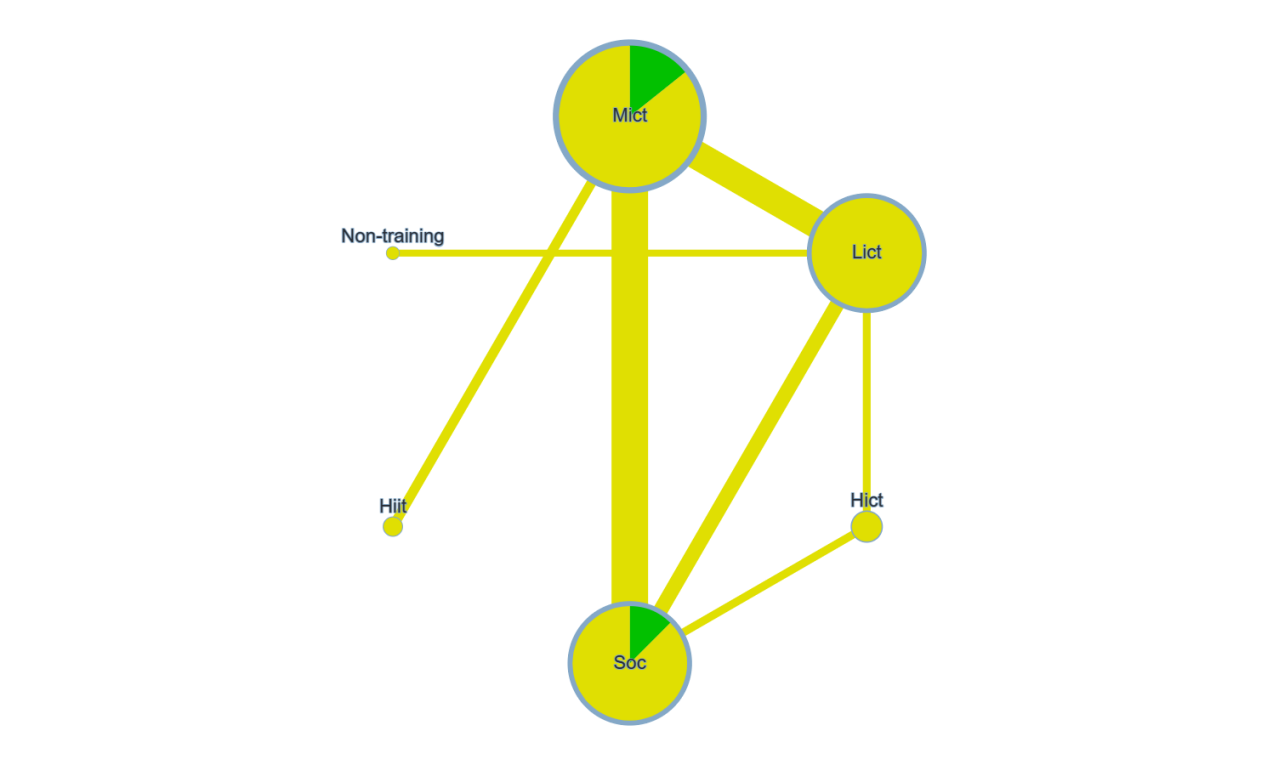


Supplementary Figure 14.CINeMA contribution plot of within-study bias (RoB 2.0) for the BBS outcome

**
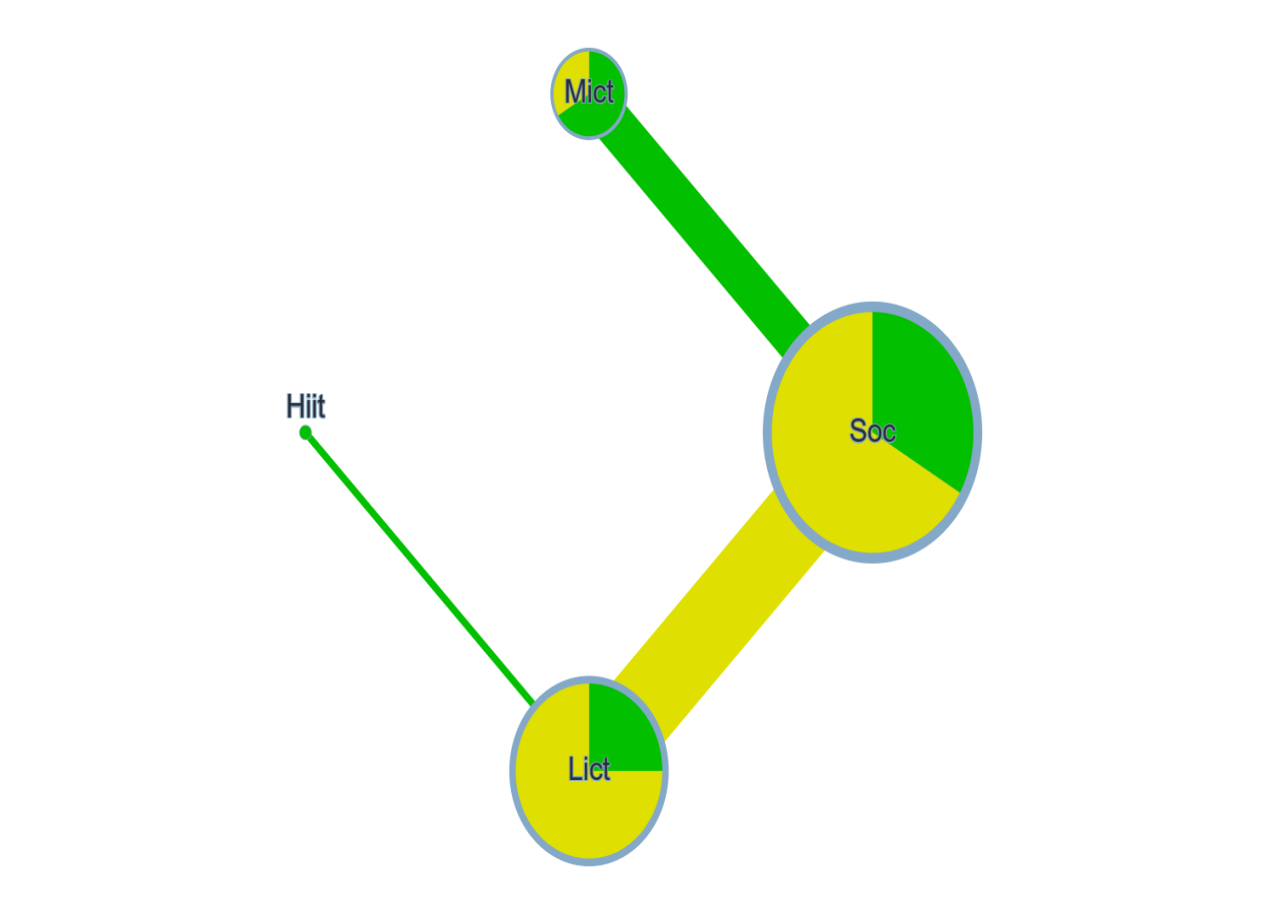
**

Supplementary Figure 15.CINeMA contribution plot of within-study bias (RoB 2.0) for the TUG outcome


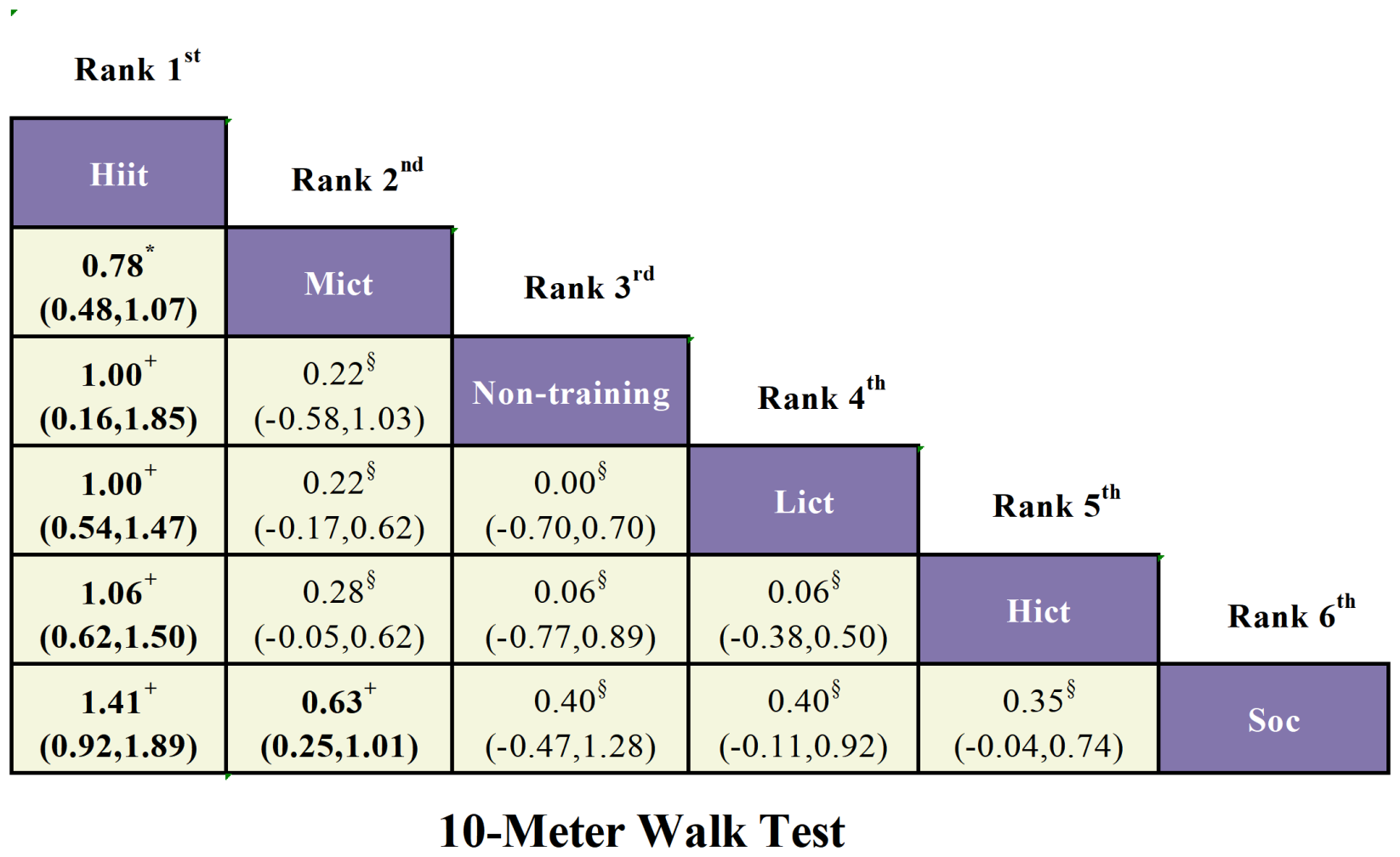


Supplementary Figure 16 League tables of treatment effects from the network meta-analysis across aerobic training intensities in chronic stroke(≥6 months): 10MWT


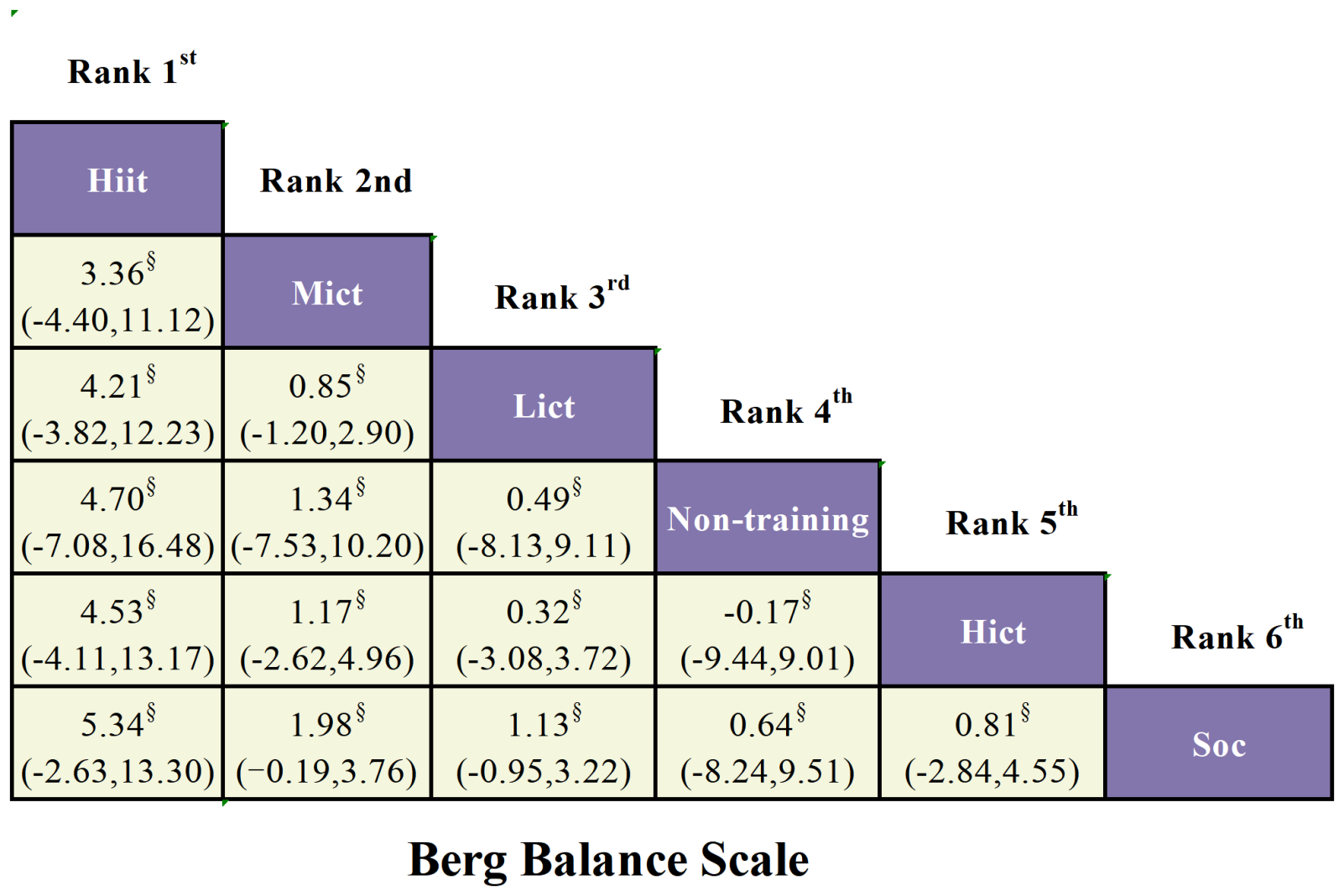


Supplementary Figure 17 League tables of treatment effects from the network meta-analysis across aerobic training intensities in chronic stroke(≥6 months): BBS


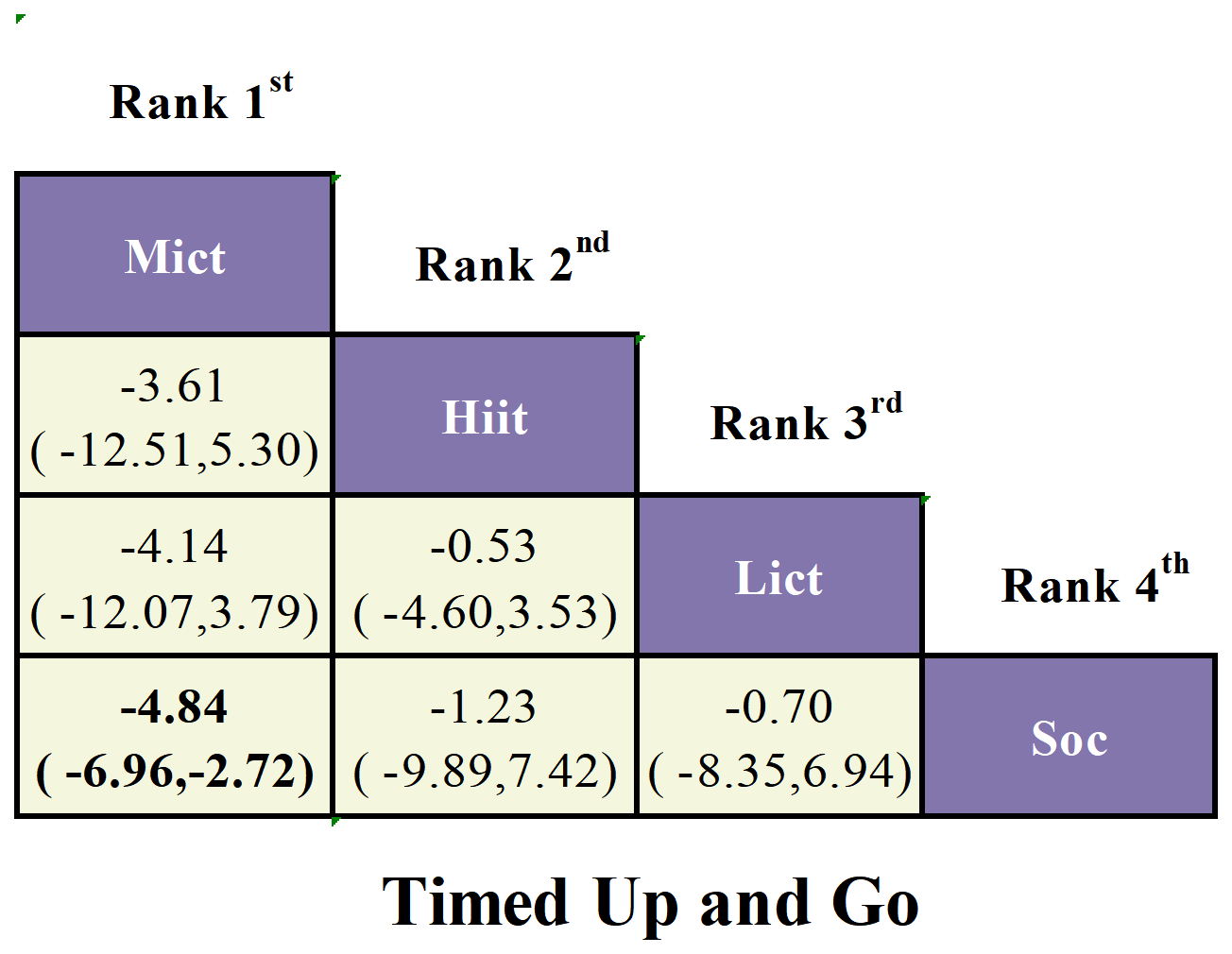


Supplementary Figure 18 League tables of treatment effects from the network meta-analysis across aerobic training intensities in chronic stroke(≥6 months): TUG
